# Molecular Hybridization of Clinically Relevant P2X7 Antagonists

**DOI:** 10.1002/cmdc.70371

**Published:** 2026-07-08

**Authors:** Nicholas B. Lynch, Charleigh T. A. Agius, André D. J. McKenzie, Jessica O’Driscoll, Angus Tolmie, Taylor R. Garrett, Eryn Werry, Michael Kassiou

**Affiliations:** ^1^ School of Chemistry Faculty of Science The University of Sydney Sydney New South Wales Australia; ^2^ Central Clinical School Faculty of Medicine and Health The University of Sydney Sydney New South Wales Australia

**Keywords:** central nervous system, neurodegenerative disease, P2X receptor, P2X7, P2X7R antagonist

## Abstract

The P2X7 receptor plays a pivotal role in the development of neurodegenerative disease states, yet no therapies targeting this receptor have reached the market to date. This work utilized a hybridization approach to combine clinically relevant antagonists AZD‐9056 and JNJ‐54175446 to discover new P2X7 antagonists. Here, we report the synthesis and in vitro and in silico evaluation of a library of 22 compounds to explore new chemical space around these clinically validated molecules. This study emphasizes the value of molecular hybridization in drug discovery and provides the basis for future P2X7 receptor antagonist development.

## Graphical abstract

We employed a molecular hybridization strategy, combining clinically validated antagonists AZD‐9056 and JNJ‐54175446, to design and synthesize a library of novel adamantane‐containing P2X7R antagonists. Pharmacological and in silico analyses indicate that these adamantyl hybrids adopt a distinct, shallow binding mode within the P2X7R allosteric pocket, differing from the deep binding mode of the parent compound JNJ‐54175446.

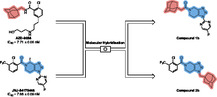

## 1 Introduction

The P2X7 receptor (P2X7R) is a ligand‐gated ion channel that maintains homeostatic ion flux throughout the central nervous system (CNS) [1]. Beyond this basal physiological role, the P2X7R also plays a key role as a danger sensing receptor, providing licensing for NLRP3 inflammasome assembly and signaling [2]. Expression of the P2X7R is near ubiquitous throughout the body, located on a diverse array of cell types, and notably upregulated on immune cells in inflammatory conditions [3].

P2X7R signaling is mediated by variations of extracellular adenosine triphosphate (eATP) concentrations. Under physiological conditions, eATP concentrations remain in the nanomolar range (10–100 nM), wherein the P2X7R plays a role in mediating healthy ion flux [4]. When stressed by pathogenic challenge or cellular insult, cells secrete increased amounts of ATP to induce an immune response mediated by P2X7R activation. At pathological concentrations of eATP (0.1–25 mM), the P2X7 receptor undergoes a conformational transition forming a large unselective pore, permeable to compounds in the hundreds of Daltons range [5, 6]. In a normal physiological system, eATP concentrations do not reach the required levels to induce pathological pore formation. If concentrations of eATP are returned to basal levels within 10–15 min, cellular homeostasis can be restored [7]. Otherwise, prolonged exposure to pathogenic amounts of eATP induces pore formation and subsequent cellular death [5, 6], sustaining pathological states and further inducing P2X7R activation [8].

Generally, under pathogenic insult or cellular injury, acute inflammation is necessary to eliminate the initial trigger and promote cellular repair [9]. In a healthy immune system, this acute inflammation is resolved quickly and homeostasis is restored [9]. In chronic inflammatory pathologies, P2X7R receptor‐mediated pore formation causes cell death often disproportionate with the severity of the initial trigger. This chronic inflammatory pathology is common in neurodegenerative disease such as Alzheimer’s disease [10, 11], Parkinson’s disease [12, 13], and amyotrophic lateral sclerosis [14, 15]. Studies have suggested that P2X7R inhibition exerts a neuroprotective effect in animal models of these neurodegenerative diseases [16, 18]. Furthermore, P2X7R knock‐out mice show a significantly attenuated inflammatory response compared to controls [19]. Collectively, these findings underscore the critical need to develop potent and selective P2X7R antagonists to advance both mechanistic understanding and therapeutic interventions in neurodegenerative disease.

**FIGURE 1:**
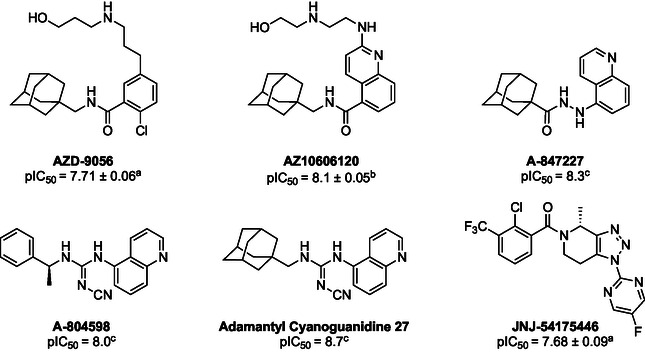
Structures of adamantane‐containing P2X7R antagonists and both A‐804598 and JNJ‐54175446. ^a^pIC_50_ measured in human THP‐1 cells using YO‐PRO‐1 dye uptake functional assay. ^b^pIC_50_ measured using two‐electrode voltage clamp. ^c^pIC_50_ measured in human THP‐1 cells using IL‐1β release assay.

Among the plethora of P2X7R antagonists that have been disclosed to date, adamantane‐containing compounds remain consistently over‐reported in the literature. Work from AstraZeneca disclosed compounds **AZD‐9056** and **AZ10606120** (Figure 1), as highly potent and selective P2X7R antagonists.

**AZD‐9056** progressed into multiple phase II clinical trials for rheumatoid arthritis [20], Crohn’s disease [21], and hidradenitis suppurativa [22]. Although it demonstrated favorable safety, tolerability, and pharmacokinetic properties, it failed to achieve clinical endpoints for these indications. Nevertheless, its robust safety profile and well‐characterized pharmacology have established it as a widely used in vitro tool compound for evaluating P2X7R antagonism [23, 24]. Concurrently, Abbott Pharmaceuticals disclosed adamantane‐containing hydrazide **A‐847227** (Figure 1) with good potency albeit with poor metabolic stability [25]. Subsequent SAR studies from Abbott concluded that the adamantane was necessary to achieve potent antagonism and could not be replaced. Similarly, work from O’Brien‐Brown et al. showed that hybridization of adamantane onto the core scaffold of **A‐804598** (Figure 1) improved the scaffold’s potency nearly fivefold [26]. Again, the **Adamantyl Cyanoguanidine 27** (Figure 1) ultimately suffered from high rates of clearance in vitro and poor metabolic stability.

In parallel, Janssen Pharmaceuticals have developed a broad portfolio of structurally distinct P2X7R antagonists, advancing several compounds into clinical development [27, 28], disclosing extensive patent coverage and continuing to publish novel chemotypes [29, 31]. Among these, **JNJ‐54175446** (Figure 1) progressed to Phase II clinical trials for major depressive disorder and demonstrated an acceptable safety profile but failed to meet clinical endpoints [32]. Importantly, **JNJ‐54175446** demonstrated CNS penetration, acceptable metabolic stability, and high plasma concentrations of free drug in human studies [32].

The clinical failure of **AZD‐9056** and **JNJ‐54175446** may reflect broader challenges within P2X7R drug discovery rather than deficiencies in the compounds preclinical profile. Poor clinical translation has become increasingly recognized in the field, driving ongoing efforts to develop more predictive disease models [33, 34]. Their lack of efficacy may therefore stem from indication selection and trial design rather than each drug’s pharmacokinetic insufficiency [32]. Nevertheless, the progression of both compounds into clinical development demonstrated favorable safety, tolerability, and drug‐like properties, validating these chemotypes as suitable templates for continued development.

**FIGURE 2:**
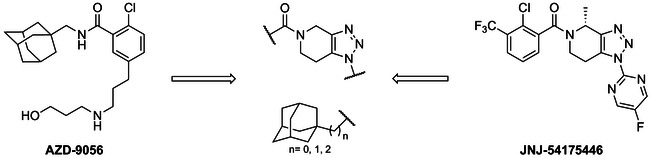
Cinical candidates AZD‐9056 (left) and JNJ‐54175446 (right), the parent scaffolds selected for molecular hybrisation.

In this study, we applied an exploratory hybridization approach by combining the adamantane motif over‐represented in literature with a simplified analog of the core scaffold of **JNJ‐54175446** (Figure 2). Given the advanced and preclinically validated molecules used as starting points, we aimed to create a library of compounds biased toward favorable drug‐like and ADMET properties. We hypothesized that the incorporation of the adamantane motif would maintain the established hydrophobic interactions important for P2X7R antagonism, while the JNJ‐derived heterocyclic core could provide improved physicochemical properties and additional binding interactions. Removal of the core’s stereocenter increased synthetic tractability whilst only moderately decreasing analog potency. To validate this approach, we then synthesized a variety of nonadamantane analogs to determine whether simple carbocycles can retain antagonistic activity in place of adamantane. Through this strategy, we sought to access novel P2X7R antagonist scaffolds while retaining the favorable drug‐like characteristics associated with the **JNJ‐54175446** scaffold.

## 2 Results and Discussion

### 2.1 Molecular Hybrid Design

**FIGURE 3:**
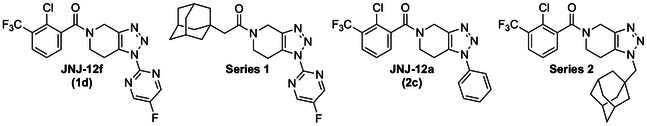
Proposed molecular hybrids with methylene linker spacers indicated.

Given the exploratory nature of this study, we opted to use a simplified scaffold of **JNJ‐54175446**, omitting the chiral methyl group, to improve the synthetic accessibility of these analogs. With the aim of keeping the triazolopyridine core intact, we opted to make substitutions varying through the amide (**Series 1**, Figure 3) and 1‐position of the triazolopyridine (**Series 2**, Figure 3).

To enable valid comparisons across scaffolds, we also resynthesized benchmark compounds **JNJ‐12f** (**1d**) and **JNJ‐12a** (**2c**) originally disclosed by Savall et al. [29], for direct comparison within each series. To increase the likelihood of retaining potent P2X7R antagonism within this scaffold, we designed five analogs incorporating variations in methylene linker spacing.

### 2.2 In Silico Evaluation of Proposed Molecular Hybrids

Having designed a small library of adamantane‐containing molecular hybrids, we evaluated their predicted potency using induced‐fit docking in Maestro 14.5 [35, 37] within the Schrodinger suite. The proposed compounds were subsequently screened with ADMETLab3.0 [38] to identify any predicted ADMET liabilities. To validate our in silico approach, we additionally evaluated the benchmarks compounds **JNJ‐12f** (**1d**) and **JNJ‐12a** (**2c**) originally disclosed by Savall et al. [29], alongside both **AZD‐9056** and **JNJ‐54175446** (Table 1).

**TABLE 1:** In silico predictions of GlideScores and preclinical markers of bioavailability calculated using Maestro 14.5 [35, 37] and ADMETLab 3.0 [38], respectively.

Calculated properties	GlideScore^a^	LogD7.4^b^	F50%^c^	Plasma protein binding, %^d^	Fraction unbound, %^e^	CL_plasma_, mL/min/kg^f^
Recommended values	N/A	<1 and >3	0–0.3 (excellent) 0.3–0.7 (medium) 0.7–1.0 (poor)	≤0% (excellent) ≥90% (poor)	≥5% (excellent) ≤5% (poor)	0–5 (excellent) 5–15 (medium) ≥15 (poor)
**AZD‐9056**	−10.314	3.45	0.21	61.7	31.0	0.0003
**JNJ‐54175446**	−10.210	2.48	0.06	97.4	2.3	0.0006
**1a**	−10.172	3.11	0.002	93.8	6.6	0.01
**1b**	−9.431	3.27	0.04	81.9	17.4	0.69
**1c**	−9.873	3.59	0.03	63.2	36.1	0.06
**1d** ^g^	−10.037	2.39	0.02	96.7	2.59	0.003
**2a**	−10.778	4.07	0.002	92.9	5.7	0.1
**2b**	−10.597	3.68	0.07	93.9	5.5	0.8
**2c** g	−10.166	2.89	0.11	98.3	1.3	0.5

a Calculated GlideScore via induced‐fit docking in the human P2X7 crystal structure (PDB ID: 9E3O) using Maestro 14.5 [35, 37] in the Schrodinger suite.

b Calculated logarithm of the *n*‐octanol/water distribution coefficient at pH = 7.4.

c Classifier of a drug having bioavailability ≥50%.

d Calculated plasma protein binding at equilibrium (%).

e Calculated fraction of free drug unbound at equilibrium (%).

f Calculated intrinsic clearance of drug (mL/min/kg).

g Compounds **1o** and **2g** have previously been reported by Savall et al. [29].

In silico evaluation suggested that the proposed molecular hybrids, particularly **1b**, possess broadly comparable predicted pharmacokinetics to parent antagonists **AZD‐9056** and **JNJ‐54175446** (Table 1). Both **Series 1** and **2** generally showed improved predicted absorption, distribution, and systemic clearance relative to their literature comparators **1d** (**JNJ‐12f**) and **2c** (**JNJ‐12a**), respectively. Similarly, our calculated GlideScores indicate that these analogs may have comparable binding affinity to both reference compounds. Collectively, these predicted parameters indicate the molecular hybrids may bind to the P2X7R and display a pharmacokinetic profile similar to that of the parent antagonists. However, the values presented in Table 1 are computationally derived predictions and should be interpreted carefully as they may not reflect experimentally validated results.

### 2.3 Synthesis of Series 1

**SCHEME 1:**
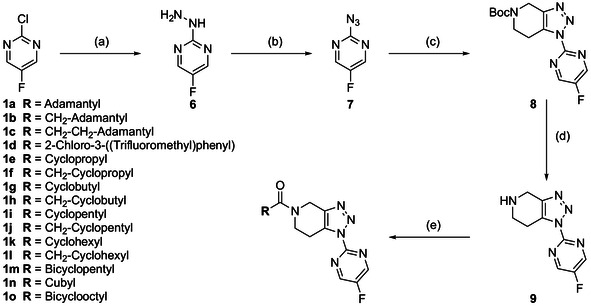
Synthesis of p‐fluoropyrimidyl‐triazolopyridines: (a) N_2_H_4_·H_2_O, EtOH, 80°C, 16 h, 78%; (b) NaNO_2_, AcOH/H_2_O (3:1, v/v), r.t., 3 h, 87%; (c) (i) *tert*‐butyl 4‐oxopiperidine‐1‐carboxylate, pyrrolidine, PhMe, 110°C, 16 h; (ii) NaHCO_3_, *m*‐CPBA, CH_2_Cl_2_, 0°C, 2 h, 64%; (d) 4 M HCl·1,4‐dioxane, r.t., 2 h, 96%. (e) Corresponding carboxylic acid, HATU, Et_3_N, CH_2_Cl_2_, r.t., 0.25–16 h or (i) corresponding carboxylic acid, (COCl)_2_, CH_2_Cl_2_, DMF, r.t., 1 h. (ii) Et_3_N, CH_2_Cl_2_, r.t., 0.25–16 h, 21%–70%.

Syntheses aiming to explore substitutions at the pendant amide began with the construction of the triazolopyridine core, diverging via coupling with the corresponding carboxylic acid (Scheme 1). Compound **1d**, previously described by Savall et al. [29], was synthesized as a reference standard against which the potency of novel antagonists could be assessed.

Initially, 2‐chloro‐4‐fluoropyridine was converted to the corresponding hydrazide (**6**) and subsequently diazotized to yield azide (**7**) (Scheme 1). Stability testing of azide **7** had been previously reported and thoroughly examined before handling quantities greater than 1 g [31]. A 2 + 3 cycloaddition of azide **7** with *tert*‐butyl 4‐oxopiperidine‐1‐carboxylate followed by a cope elimination yielded the *N*‐boc‐protected amine (**8**). The free amine (**9**) was afforded by acidic hydrolysis of the *N*‐boc‐protected amine and consequently coupled with the corresponding carboxylic acids to yield products **1a–1o** in moderate yields (21%–70%) (Scheme 1).

### 2.4 Synthesis of Series 2

**SCHEME 2:**
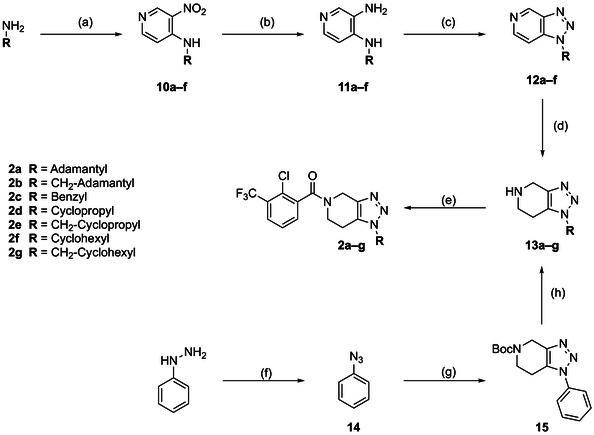
Synthesis of 2‐chloro‐3‐((trifluoromethyl)phenyl)‐triazolopyridines: (a) corresponding amine, Et_3_N, DMF, r.t., 0.5 h, 75%–93%; (b) 5% Pd/C, H_2_, MeOH, r.t., 16 h, 85%–98%; (c) (i) conc. HCl, r.t., 0.25 h; (ii) NaNO_2_, H_2_O, 0°C to r.t., 2 h, 81%–93%; (d) H‐Cube Pro, 5% Rh/C, MeOH/AcOH, 10 bar, 40°C, 1 mL/min, product recycling, 2 h, 45%–60%; (e) 2‐chloro‐3‐(trifluoromethyl)benzoic acid, HATU, Et_3_N, CH_2_Cl_2_, r.t., 73%–95%; (f) ^*t*^BuONO, AcOH, MeCN, r.t., 0.5 h; (g) (i) *tert*‐butyl 4‐oxopiperidine‐1‐carboxylate, pyrrolidine, PhMe, 110°C, 16 h; (ii) NaHCO_3_, *m*‐CPBA, CH_2_Cl_2_, 0°C, 2 h, 71%; (h) 4 M HCl·1,4‐dioxane, r.t., 2 h, 92%.

Syntheses aiming to explore substitutions in place of the fluoropyrimidine could not be accessed in a divergent manner as seen in Scheme 1. As such, a stepwise synthesis of the core followed by coupling with 2‐chloro‐3‐(trifluoromethyl)benzoic acid was attempted (Scheme 2). Given that adamantane has proven a bioisostere of benzyl groups in the P2X7R drug design space [26, 39, 40], benzyl containing compound **2c** was also synthesized. First reported by Savall et al. [29], this compound served as a reference standard against which the potency of novel antagonists were evaluated.

Initially, the corresponding amines were coupled with 4‐chloro‐3‐nitropyridine via nucleophilic substitutions in excellent yields (**10a–f**, 75%–93%) (Scheme 2). Compounds (**10a–f**) were then reduced via transition metal‐catalyzed hydrogenation, forming di‐amines (**11a–f**) and subsequently diazotized to form the triazole core (**12a–f**) (Scheme 2). The dearomatization of pyridines (**12**) to tetrahydropyridines (**13**) was achieved via high‐pressure hydrogen transition metal catalysis using a H‐Cube pro. The free amines (**13a–g**) were then coupled with 2‐chloro‐3‐(trifluoromethyl)benzoic acid via standard amide coupling conditions to afford compounds **2a–2g** in good yields (73%–95%) (Scheme 2).

### 2.5 Pharmacological Evaluation of Hybrids

Gratifyingly, hybrids from both series retained modest potency, with compound **1b** showing potency comparable to reference compound **1d** (Table 2) in a dye uptake assay. **Series 1** was better tolerated than **Series 2**, indicating that this scaffold can better accommodate steric bulk introduced via the amide rather than through the triazole. Interestingly, compound **1b** appears to have the optimal methylene linker spacing within this scaffold with both further extension (**1c**) and removal (**1a**), resulting in decreased potency. Work from both Wilkinson et al. [41] and Oken et al. [42], has shown that the P2X7R can comfortably accommodate polycycles larger than adamantane. There is no direct correlation between polycyclic volume and antagonist potency at the P2X7R, indicating that polycyclic shape is also necessary to effect potent antagonism [42]. Having established that our adamantane‐containing hybrids retained potency within this scaffold, we next sought to determine whether this activity was unique to adamantane or extended across alternative carbocyclic scaffolds. We synthesized a variety of smaller carbocycles and polycycles to determine the minimum steric bulk required to replicate the potency seen in the adamantyl analogs. Both SAR studies and patent disclosures from Janssen Pharmaceuticals either do not explore or have omitted simple carbocyclic substitutions in these positions.

**TABLE 2:** IC_50_ values of molecular hybrids against hP2X7R using the YO‐PRO‐1 dye uptake functional assay in human THP‐1 cells.

Series 1 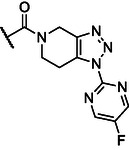				Series 2 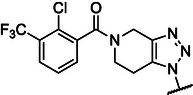				
Compound		R	pIC_50_ a (dye uptake)	Compound		R		pIC_50_ a (dye uptake)
**1a**	(*n* = 0)	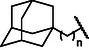	6.27 ± 0.09	**2a**	(*n* = 0)		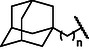	5.53 ± 0.04
**1b**	(*n* = 1)		7.04 ± 0.03	**2b**	(*n* = 1)			5.33 ± 0.15
**1c**	(*n* = 2)		6.66 ± 0.03					
**1d**		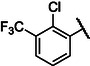	7.70 ± 0.08	**2c**			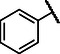	7.46 ± 0.04
**JNJ‐54175446**		—	7.68 ± 0.09	**AZD‐9056**			**—**	7.71 ± 0.06

a pIC_50_ values are the mean of three experiments (*n* ≥ 3), with the uncertainty reported as the standard error of the mean (SEM).

Carbocyclic substitutions in **Series 1**, both directly attached to the core and linked via a methylene spacer (**1e**–**1k**), showed no appreciable activity (Table 3). As substituent size increased, seen in analog **1l**, some potency was restored, although still markedly lower than the adamantyl‐containing analogs, **1a**–**1c**. Interestingly, smaller polycyclic moieties such as cubane, bicyclopentane, and bicyclooctane (**1m–1o**) proved functionally inactive (Table 3). These trends regarding smaller polycycles reaffirm findings from Wilkinson et al., in the benzamide scaffold, although the triazolopyridine scaffold appears much less accommodating to these alterations in steric bulk [43]. Substitutions from Series 2 were tolerated well comparative to Series 1. While compounds **2d – 2g** are weakly active, the trend in potency clearly indicates that an increase in steric bulk decreases the compound’s potency. However, this trend directly contradicts the potency seen in the adamantyl analogs. The potency of compounds **2a** and **2b** (pIC_50_ = 5.5 and 5.4, respectively) is comparable to compounds **2e** and **2f** (pIC_50_ = 5.3 and 5.4, respectively) despite the extremely substantial difference in size. To probe this further, we calculated the Van der Waals surface area (vdWSA) of each carbocyclic substitution, looking at the relationship between size and potency of these substitutions made (Table 4)

**TABLE 3:** IC_50_ values of molecular hybrids using the YO‐PRO‐1 dye uptake functional assay in human THP‐1 cells.

Series 1 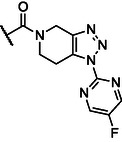				**Series** 2 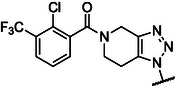			
Compound		R	pIC_50_ ± SEM^a^ (dye uptake)	Compound		R	pIC_50_ ± SEM^a^ (dye uptake)
**1e**	(*n* = 0)	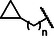	>5	**2d**	(*n* = 0)	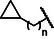	5.91 ± 0.03
**1f**	(*n* = 1)		>5	**2e**	(*n* = 1)		5.32 ± 0.04
**1g**	(*n* = 0)	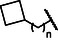	>5	**2f**	(*n* = 0)	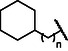	5.30 ± 0.03
**1h**	(*n* = 1)		>5	**2g**	(*n* = 1)		>5
**1i**	(*n* = 0)	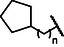	>5				
**1j**	(*n* = 1)		>5				
**1k**	(*n* = 0)	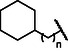	>5				
**1l**	(*n* = 1)		5.55 ± 0.05				
**1m**	—	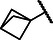	>5				
**1n**	—	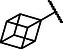	>5				
**1o**	—	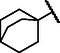	>5				

a pIC_50_ values are the mean of three experiments (*n* ≥ 3), with the uncertainty reported as the standard error of the mean (SEM).

**TABLE 4:** Van der Waals surface area (vdWSA) of substituents introduced in **Series 2.**

Compound	**pIC**_**50**_ **± SEM** a	**Van der Waals surface area (vdWSA)** b	Compound	**pIC**_**50**_ **± SEM** a **(dye uptake)**	**Van der Waals surface area (vdWSA)** ^**b**^
**2a**	5.53 ± 0.04	270.8	**2b**	5.33 ± 0.15	298.9
**2d**	5.91 ± 0.03	124.5	**2e**	5.32 ± 0.04	156.0
**2f**	5.30 ± 0.03	207.8	**2g**	>5	238.6
**2c**	7.46 ± 0.04	163.9			

a pIC_50_ values are the mean of three experiments (n ≥ 3), with the uncertainty reported as the standard error of the mean (SEM).

b vdWSA calculated using the 3D Molecular Surface area tool in MarvinSketch 24.1.2. Volumes calculated using R Group attachment in place of the core as denoted in Tables 2 and 3.

Intuitively, the increase in size from **2d** (vdWSA = 124.5) to **2f** (vdWSA = 238.6) correlates to a decrease in potency. Similarly, the benzyl group of **2c** can participate in favorable π–π stacking, π‐cation, or other aromatic interactions which may explain the increase in potency relative to the nonaromatic counterpart **2d**. However, the adamantyl analogs **2a** and **2b** are significantly larger than compound **2g** and yet they still maintain activity, comparable to compounds **2e** and **2f**. These results, in combination with the results seen in Table 2, suggest that the adamantyl‐containing compounds may display privileged binding behavior within this scaffold. To investigate these findings further, we conducted both a pharmacological evaluation of the mode of antagonism and an in silico analysis of their potential binding modes.

### 2.6 Compound 1b is a Non‐Competitive P2X7R Antagonist

**FIGURE 4:**
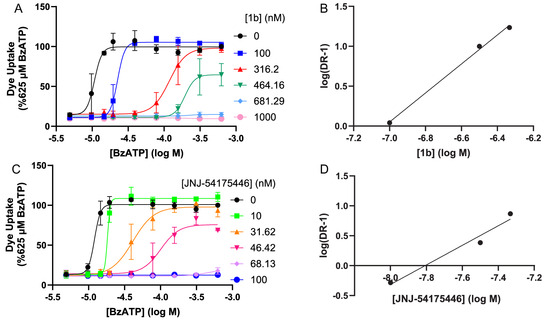
Schild analysis of and **JNJ‐54175446**. HEK‐293 hP2X7R cells were preincubated with either **1b** or **JNJ‐54175446** for 1 h at 28°C and then exposed to varying concentrations of BzATP and YO‐PRO 1. The average EC_50_ values for all runs were used to calculate the dose‐ratio (DR; the ratio of EC_50_ in the presence of antagonist, compared to the EC_50_ in the absence of antagonist). Agonist dose–response curves were presented as mean ± SEM (*n* ≥ 3), in the presence of **1b** (A) and **JNJ‐54175446** (C). Corresponding Schild plot was conducted to determine binding of **1b** (B) and **JNJ‐54175446** (D).

Before making further inferences on the binding mode of these antagonists, we first sought to confirm that both compound **1b** and **JNJ‐54175446** display the same mode of antagonism at the P2X7 receptor. We first carried out a washout assay to show that both **1b** and **JNJ‐54175446** display reversible binding kinetics before conducting a Schild analysis (Section S1.1.2). To validate our assay, we also analyzed the binding mode of a well‐studied P2X7R antagonist, **A‐804598**, which has been previously reported to display a competitive binding mode [44, 45]. Our Schild analysis of **A‐804598** confirmed that it was BzATP competitive, displaying a parallel concentration–effect curve that yielded a Schild regression slope of 1.007 (95% CI; 0.44–1.57). Further regression analysis found that there was no significant deviation from unity (*p* = 0.9714, *R* ^2^ = 0.91; Figure S1). Thus, these results parallel those in literature and stand as a means of comparison for **1b** and **JNJ‐54175446**. Analysis of the dye uptake results of both **1b** and **JNJ‐54175446** at 150 min demonstrated a dextral nonparallel shift of BzATP dose–response curve with a decreasing maximum (Figure 4).

BzATP curves at the two highest concentrations remained at basal levels, consistent with insurmountable noncompetitive antagonism. Schild analysis of **1b** resulted in an asymmetric rightward transformation of BzATP concentration response curves, moving in a nonparallel manner with a decreasing maximum. No BzATP response was recovered with the highest two concentrations of **1b** (Figure 4A). The corresponding Schild regression had a slope of 1.819 (95% CI: 0.55–3.13) and significantly differed from unity (Figure 4B; *p* ≤ 0.05, *r* ^2^ = 0.9969). Similarly, analysis of JNJ‐54175446 resulted in a nonparallel rightward shift of concentration–response curves, all with decreasing maximums, until the last two curves (Figure 4C). The slope for the corresponding Schild plot was 1.638 (95% CI: −2.33 to 5.61) and deviated significantly from unity (Figure 4D; *p* ≤ 0.05, *r* ^2^ = 0.9649). The last two data points for both **1b** and **JNJ‐54175446** were not included in the Schild plot as EC_50_ could not be accurately predicted due to insurmountable antagonism creating a flat dose–response curve that lacked an inflection point (Figure 4A,C). Therefore, these results confirm that both **1b** and **JNJ‐54175446** are noncompetitive P2X7R antagonists.

### 2.7 Docking Study

**FIGURE 5:**
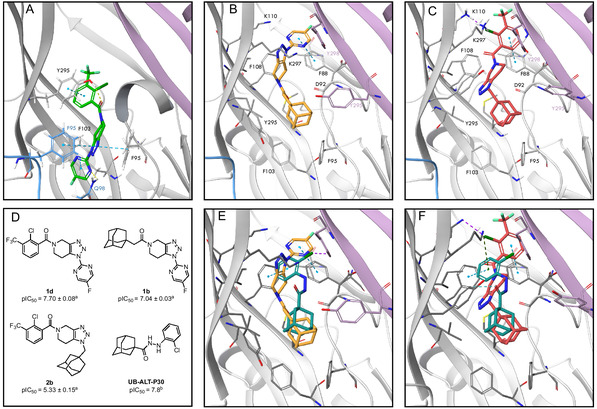
Induced‐fit docking of compounds **1d** (A) (green), **1b** (B) (yellow), and **2b** (C) (red) in the human P2X7 crystal structure (PDB ID: 9E3O). (D) Structures of compounds **1d,** **1b,** **2b,** and **UB‐ALT‐P30** with corresponding IC_50_ values. ^a^pIC_50_ calculated using the YO‐PRO‐1 dye uptake functional assay in human THP‐1 cells. ^b^pIC_50_ calculated using a calcium flux functional assay in human 1321N1 astrocytoma cells. (E) Overlay of compounds **1b** (yellow) with **UB‐ALT‐P30** (blue), highlighting the similar orientation of adamantyl groups. (F) Overlay of compounds **2b** (red) with **UB‐ALT‐P30** (blue), highlighting the similar orientation of adamantyl groups. π–π stacking interactions visualized in blue and halogen bonding visualized in purple. Docking analysis using Maestro 14.5 [35, 37]. The figure was generated using Maestro 14.5 in the Schrodinger suite.

Having established that both **1b** and **JNJ‐54175446** are noncompetetive antagonists, we then re‐examined our initial docking calculations within the human P2X7R allosteric pocket (PDB ID: 9E3O). We began by examining compound **1d** and both adamantane‐containing hybrids **1b** and **2b** in comparison to cocrystallized antagonist **UB‐ALT‐P30**. Induced‐fit docking suggests that the reference compound **1d** binds deep in the pocket analogously to **JNJ‐54157446**, as disclosed in a cryo‐EM complex from Guo et al. (Figure 5A) [33].

Recent cryo‐EM studies have shown that the P2X7R allosteric pocket has both distinct subpockets capable of accommodating antagonist binding [33] and distinct binding modes, categorized as “shallow,” “deep,” and “starfish” [46]. Docking of compound **1d** (Figure 5A) suggests it adopts a deep binding mode, analogous to the parent compound **JNJ‐54175446** 33]. In contrast, compounds **1b** and **2b** (Figure 5B,C) appear to adopt a shallow binding mode with interactions to key residues D92, F95, K110, Y295, and K297, more consistent with the shallow binding mode of **AZD‐9056**. Notably, no other carbocyclic or polycyclic analog in either series displayed this shallow binding mode, with all other compounds instead adopting deep binding poses. Taken together, these observations suggest that the shift in binding mode seen with **1b** and **2b** may be a consequence of their adamantyl moiety. The increased steric bulk and hydrophobic character of this substituent may preferentially direct these compounds toward the shallow hydrophobic subpocket. This change in binding mode may offer a structural rationale for the unusual SAR trends observed across both **Series 1** and **2.**

To further contextualize these findings, the docking poses of the adamantyl analogs **1b** and **2b** were compared to the adamantane‐containing cocrystallized ligand **UB‐ALT‐P30** (Figure 5D–F) [42]. This comparison revealed that the adamantane moieties occupy similar positions within the binding pocket. The adamantane of **UB‐ALT‐P30** occupies a highly hydrophobic subpocket between residues F95, F103, and Y295, consistent with the predicted binding of both **1b** and **2b**. These results are also consistent with the cocrystallized structure of **AZD‐9056** in the rat P2X7R (PDB ID: 8TR8) [46]. Collectively, these findings suggest that the binding behavior of **1b** and **2b** is governed, at least in part, by the hydrophobic character of the adamantyl group. Moreover, it raises the possibility that adamantane‐containing P2X7R antagonists may more broadly share a tendency to engage this allosteric subpocket.

## 3 Conclusions

This study demonstrates that molecular hybridization of an adamantane motif with the triazolopyridine core of JNJ‐54175446 affords novel P2X7R antagonists with nanomolar potency. Among a library of 22 compounds, five new adamantane‐containing P2X7R antagonists were synthesized and evaluated using a dye uptake assay. Subsequent biological evaluation identified three antagonists with nanomolar potency and two with micromolar activity. Our studies identified the minimum carbocyclic size required to retain measurable P2X7R antagonism within this scaffold and suggested distinct binding poses associated with the adamantane motif. Schild analysis of the most potent hybrid antagonists alongside a parent compound showed noncompetitive binding modes of actions for all molecules tested. Molecular docking indicated that the adamantyl group may direct antagonist binding pose, providing a possible rationale for the unexpected SAR trends observed experimentally. While further experimental and computational studies are required to validate these observations conclusively, the trends identified here appear consistent with other reported adamantane‐containing P2X7R antagonists. Future optimization efforts may benefit from rational fragment growth strategies centered around the adamantane scaffold. More broadly, these findings expand the current understanding of the structural requirements underpinning P2X7R antagonism and demonstrate the utility of molecular hybridization for accessing novel antagonist chemotypes. Collectively, this work highlights the need for further investigation to understand the role adamantane plays in P2X7R antagonism and provides a framework to guide the design of future P2X7R antagonists.

## Author Contributions

**Nicholas B. Lynch :** conceptualization, chemical synthesis, computational work, writing – original draft, review and editing. **Charleigh T. A. Agius :** conceptualization, pharmacological evaluation, writing – review and editing. **André D. J. McKenzie :** pharmacological evaluation, writing – review and editing. **Jessica O’Driscoll :** writing – review and editing. **Angus Tolmie :** writing – review and editing. **Taylor R. Garrett :** chemical synthesis, writing – review and editing. **Eryn Werry :** conceptualization, supervision, writing – review and editing. **Michael Kassiou :** conceptualization, supervision, writing – review and editing.

## Funding

This work was supported by the Australian Government Research Training Program.

## Conflicts of Interest

The authors declare no conflicts of interest.

## Supporting information

Supplementary Material

## Data Availability

Data are available in the Supporting Information.
