# Supplementary material for: Molecular Hybridization of Clinically Relevant P2X7 Antagonists
Source: ChemMedChem. 2026 Jul 8;21(13):e70371. doi: 10.1002/cmdc.70371 (PMC13346338; doi:10.1002/cmdc.70371)
Supplement: Supplementary file 1 — Supplementary Material [file CMDC-21-e70371-s001.pdf]

Supplementary Information for

Molecular Hybridisation of Clinically Relevant P2X7R Antagonists

Nicholas B. Lynch,[a] Charleigh T. A. Agius,[b] André D. J. McKenzie,[a] Jessica O'Driscoll,[a] Angus Tolmie,[a] Taylor R. Garrett,[a] Eryn Werry,[*a,b] and Michael Kassiou[*a].

[a] School of Chemistry, Faculty of Science, The University of Sydney, Sydney, NSW 2006, Australia

[b] Central Clinical School, Faculty of Medicine and Health, The University of Sydney, Sydney, New South Wales 2006, Australia

Corresponding Authors

*E-Mail: eryn.werry@sydney.edu.au, Phone: +612 9351 8634

*E-Mail: michael.kassiou@sydney.edu.au, Phone: +612 9351 2745

Contents

1 Biological Experimental **2**

1.1 Methods 2

1.1.1 Dye Uptake Assay 2

1.1.2 Washout Assay 3

1.1.3 Schild Analysis 3

1.2 Biological Data 4

2 Chemistry Experimental **6**

2.1 Computational Methods 6

2.2 General information for Chemical Synthesis 6

2.3 General Methods 8

2.3.1 General Procedure A 8

2.3.2 General Procedure B 8

2.3.3 General Procedure C 9

2.3.4 General Procedure D 9

2.3.5 General Procedure E 10

2.3.6 General Procedure F 10

2.4 Synthesis and Characterisation 11

2.4.1 Synthesis and characterisation of compounds **1a-o** 11

2.4.2 Synthesis and characterisation of compounds **2a-g** 24

2.5 HPLC Traces of biologically tested compounds 43

2.6 NMR Spectra of biologically tested compounds 65

3 Bibliography **109**

Chapter 1

Biological Experimental

1.1 Methods

1.1.1 Dye Uptake Assay

THP-1 cells were cultured in RPMI1640 (Gibco) with 10% FBS (Gibco) at 37 °C, 5% CO_2. Cells were resuspended in RPMI1640 with 5% FBS, LPS (from E. coli strain 0111:B4; Sigma; 100 ng/mL) and recombinant human interferon gamma (rhIFN-γ; R&D Systems; 10 ng/mL). Cells were seeded into 96-well black-walled CellBIND plates (Corning) at a density of 1.5 x 10^5 cells/well and were allowed 22–24 h to differentiate (37 ° C, 5% CO_2). Media was aspirated and cells washed once with 150 µL of Hanks' Balanced Salt Solution (HBSS; KCl 5.37 mM, KH_2PO_4 0.44 mM, NaCl 136.89 mM, Na_2HPO_4 0.34 mM, glucose 5.55 mM, $NaHCO_3$ 4.17 mM, $CaCl_2$ 0.1 mM, pH 7.4). Cells were then treated with 100 µL HBSS containing 1 µM YO-PRO-1® Iodide (Invitrogen), 100 µM 2'(3')-O-(4-Benzoylbenzoyl) adenosine-5'-triphosphate (BzATP; 100 µM; Sigma-Aldrich), (or ultrapure water vehicle) and the test compound (or DMSO vehicle). Fluorescence was recorded within each well approximately every 30 s for 2 h using a BMG POLARstar Omega (λex = 485-12, λem= 520). To determine the IC_{50}, fluorescence values at 1 h were analysed from each experiment and were fitted using a 4-parameter sigmoidal dose-response (inhibition) fit with GraphPad Prism 10 (GraphPad Prism Software). Each test compound was assayed in duplicate a minimum of 3 times, and the IC_{50} from each assay was used to derive the arithmetic mean and standard deviation reported in the tables. All curves with a poor goodness of fit ($R^2 \leq 0.9$) were excluded, and data was omitted if deemed an outlier through $p < 0.05$ in Grubb's Test (GraphPad Prism). Values are presented as mean ± standard deviation (S.D.).

1.1.2 Washout Assay

HEK-293 hP2X7R cells were harvested by enzymatic dispersion using trypsin-EDTA (0.25% trypsin, 0.53 mM EDTA in DBPS) and resuspended with DMEM/F12 medium supplemented with 5% FBS without geneticin. Cells were seeded at a density of 2 x 10^4 cells/well onto a 96-black-walled CellBIND plate coated with 100 µg/mL poly-L-lysine (Sigma-Aldrich) and incubated for 22-24 h (37 °C, 5% CO_2). Cell supernatant was aspirated, and cells were treated with 100 µL of NMDG buffer (NaCl 140 nM, glucose 10 mM, HEPES 10 mM, KCl 5.6 nM, N-methyl-D-glucamine 6 mM, $CaCl_2$ 0.5 mM, pH 7.4) supplemented with test compound, vehicle control 1 (0.1 % DMSO), or vehicle control 2 (MilliQ water) and incubated for 40 mins (37 °C, 5% CO_2). Following incubation, media was aspirated from all conditions and cells were treated with 100 µL NMDG buffer containing 1 µM YO-PRO-1® Iodide (Invitrogen) (1 µM) and either ATP (1 M) or vehicle control (MilliQ water). YO-PRO-1® dye uptake was then measured as previously reported.

1.1.3 Schild Analysis

Cells were harvested and plated as per **1.1.2**. Following a 22-24 h incubation period, media was removed, and cells were washed with 150 µL of warm NMDG buffer (37 °C). Cells were then treated with either vehicle control (0.1% DMSO) or compound (10 nM – 1 µM) and incubated for 1 hour at 25 °C in a non-CO_2 incubator to allow receptor-ligand equilibration. Cells were removed and treated with YO-PRO-1® dye (1 µM), compounds (10 nM –1 µM) or vehicle control (0.1% DMSO) and BzATP (9.77 µM – 1250 µM). Fluorescence was read at a 28 °C to prevent a bell-shaped concentration curve from formining on application of high BzATP concentrations, which appears at 37 °C.[1] Data was taken at 150 minutes to ensure curve plateauing and that equilibrium between the receptor, agonist and antagonist had been achieved. EC_{50} values for each concentration were calculated using a sigmoidal agonist concentration response curve with variable slope (4 parameters) non-linear regression fit in Prism 10. Average EC_{50} values from each repetition were used to calculate the dose ration (DR). The DR was determined and plotted as a simple linear regression, with log (DR^{-1}) versus log [B], where [B] is

the antagonist concentration. The resulting Schild slope was then analysed for significant difference from unity ($p < 0.05$) using a one-sample t-test in Prism 10.

1.2 Biological Data

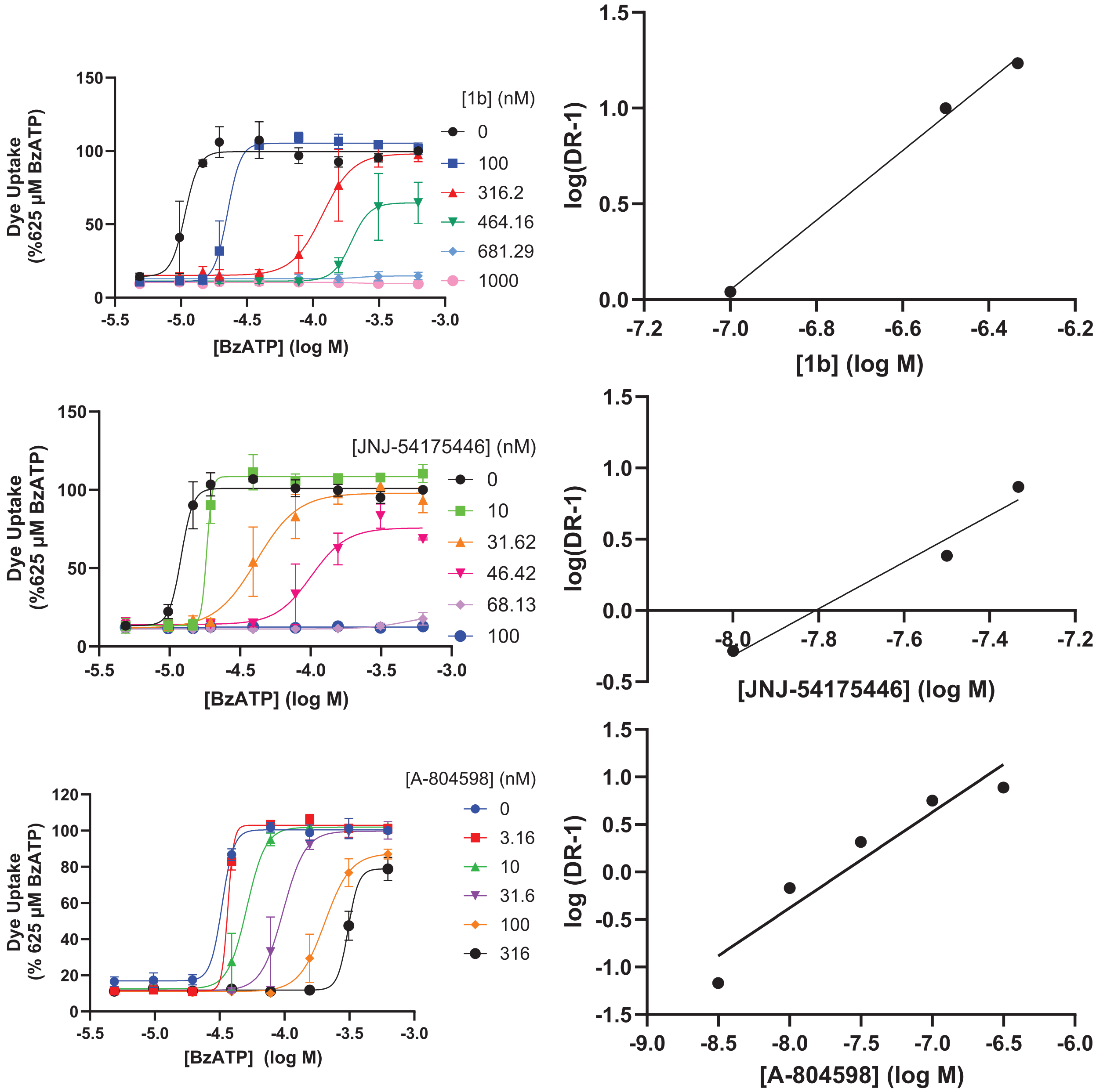

Figure 1: Dye Uptake and corresponding schild plots of compound **1b**, **JNJ-54175446** and **A-804598**

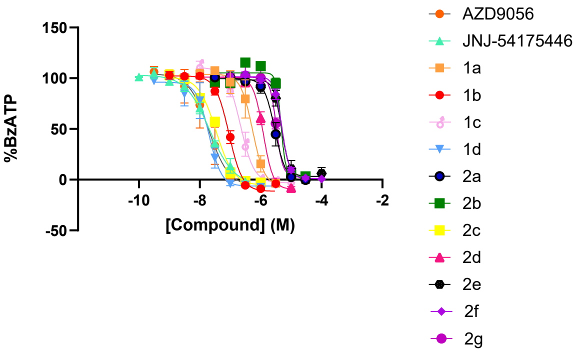

Figure 2: Dose response curves of all biologically active compounds evaluated

Table 1: pIC_{50} ± SEM, Hill Slope and R^2 values of biologically active compounds evaluated

Compound Number	pIC_{50} ± SEM[a]	Hill Slope	R^2
**1a**	6.27 ± 0.09	-2.23	0.9606
**1b**	7.04 ± 0.03	-1.97	0.9057
**1c**	6.66 ± 0.03	-1.93	0.9881
**1d**	7.70 ± 0.08	-2.11	0.9277
**1l**	5.55 ± 0.05	-1.98	0.9512
**2a**	5.53 ± 0.04	-2.41	0.9494
**2b**	5.33 ± 0.15	-5.59	0.9696
**2c**	7.46 ± 0.04	-1.50	0.9877
**2d**	5.91 ± 0.03	-2.67	0.9650
**2e**	5.32 ± 0.04	-3.34	0.9829
**2f**	5.30 ± 0.03	-3.42	0.9916
**AZD-9056**	7.71 ± 0.06	-1.32	0.9205
**JNJ-54175446**	7.68 ± 0.09	-1.10	0.9671

Chapter 2

Chemistry Experimental

2.1 Computational Methods

All molecular docking studies were performed within the Maestro software package (14.5, 2025-1, Schrodinger Inc., New York, USA) within the centre for Drug Design and Innovation *In Silico* Drug Design Facility at the School of Pharmacy, University of Sydney. The human P2X7 receptor crystal structure (PDB: 9E3O) was imported directly from PDB and prepared using the Protein Preparation Wizard embedded in Maestro. The hydrogen bond networks within the protein were optimised with all het groups within the receptor bounding box removed. The protein structure was minimised to a RMS deviation of 0.2 Åusing the OPLS4 force field. The Glide Receptor Grid Generation tool was used to build a 5 Åbounding box centered on UB-ALT-P30 as a reference ligand within the active site of the receptor. The ligands were prepared using LigPrep to generate protonation states at pH 7.0 $\pm$ 2.0 and ConfGen was used to obtain 64 different conformers of each ligand. Ligands were docked into the receptor grid using the Induced Fit Docking Tool. The conformer with the lowest Glidescore of each compound was analysed.

2.2 General information for Chemical Synthesis

Reagents were obtained from commercial sources and used without further purification unless otherwise stated. Dry solvents were obtained from a PureSolv MD 7 solvent purification system (Innovative Technology, Inc.). Reactions were conducted under a positive pressure of a dry nitrogen atmosphere unless otherwise specified. Analytical thin-layer chromatography (TLC) were performed on Merck 0.2 mm aluminium-backed silica gel 60 F254 plates which were visualised with shortwave (254 nm) and/or longwave (365 nm) ultraviolet (UV) light. Compounds without a UV active chromophore were visualised with p-anisaldehyde, cerium molybdate, potassium permanganate or vanillin.

Flash chromatography was performed using Merck silica gel 60 (230–430 mesh). All solvent compositions are reported as their volume/volume ratios.

^{1}H NMR Spectra were recorded at 300 K unless otherwise stated, using either a Bruker AVANCE DRX300 (300 MHz), DRX 400 (400 MHz), or AVANCE II 500 Ascend (500 Mhz). Chemical shifts (σ ppm) are reported relative to the residual solvent peak. Signal multiplicities are assigned as singlet (s), doublet (d), triplet (t), quartet (q), pentet (p), doublet of doublets (dd), or multiplet (m), with the observed coupling constant (J) described in Hertz (Hz). Nuclear assignments were performed with the aid of COSY, HSQC and HMBC experiments where necessary. Low-resolution mass spectra (LRMS) were recorded on a Bruker amazon SL mass spectrometer using electrospray ionisation (ESI) with samples directly infused using a Cole Palmer syringe pump. High-resolution mass spectra (HRMS) were recorded on a Thermo Velos Pro Orbitrap mass spectrometer and performed by the Mass Spectrometry Facility of the School of Chemistry at the University of Sydney. Purity analysis was performed by analytical high performance liquid chromatography (HPLC) on a Waters Alliance 2695 apparatus equipped with a waters 2996 photodiode array detector, set at 254 nm. Separation using a SunFireTM C18 column (5µm, 2.1 × 150 mm) was achieved using water (solvent A) and acetonitrile (solvent B) at a flow rate of 0.2 mL/min. The method consisted of 0% B to 100% B over 30 minutes. HPLC data is recorded as percentage purity and retention time (R_T) in minutes.

Note: Spectra for compounds **1a-o** and **2a-g** are reported as a complex mixture of conformers. Variable temperature NMR did not show coalescence of peaks with both high temperature and low temperature experiments. See section 2.6 for 2D analysis of compound **2c**.

2.3 General Methods

2.3.1 General Procedure A

Acid chloride mediated amide coupling of carboxylic acids with secondary amines.

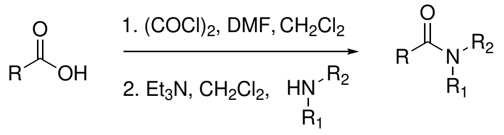

To a solution of carboxylic acid (1.0 eq.) in CH_2Cl_2 (15 mL/mmol) at 0 °C was added oxalyl chloride (1.2 eq.). DMF (1 drop) was added and the solution was allowed to come to room temperature. The reaction mixture was stirred at room temperature until completion by TLC or 3 hours, whichever came first. The reaction mixture was then concentrated under a stream of N_2 yielding the desired acid chloride. The acid chlorides were then used without further purification.

To a solution of acid chloride (1.2 eq.) in CH_2Cl_2 (15 mL/mmol) was added Et_3N (1.5 eq.), followed by the corresponding secondary amine (1.0 eq.). The reaction was stirred at room temperature until completion by TLC or 3 hours, whichever came first. The reaction mixture was quenched in Na_2CO_3 (25 mL/mmol) and extracted with CH_2Cl_2 (3 × 50 mL/mmol). The organic phases were combined, dried over $MgSO_4$, and concentrated *in vacuo*. The crude product was purified by flash column chromatography (EtOAc/Hex or MeOH/CH_2Cl_2 as eluents) to yield the desired product.

2.3.2 General Procedure B

Nucleophilic aromatic substitution of 4-chloro-3-nitropyridine

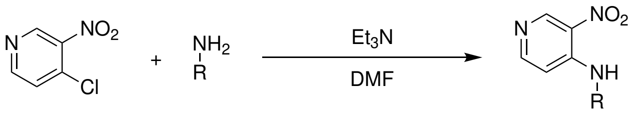

A solution of 4-chloro-3-nitropyridine (1.0 eq.) in DMF (15 mL/mmol) was treated with Et_3N (2.0 eq.) and the corresponding primary amine (1.5 eq.). For aryl amines, the reaction mixture was heated to either the boiling point of the amine, or 80 °C, whichever

was lower. The reaction was then let stir until completion by TLC or 16 hours, whichever came first. For alkyl amines, the reaction mixture was stirred at room temperature for 15 minutes. The reaction mixture was allowed to cool to room temperature and diluted into EtOAc (40 mL/mmol). The organic phase was then washed with H_2O (5 × 25 mL/mmol), brine (1 × 25 mL/mmol), dried over $MgSO_4$, and concentrated *in vacuo*. The crude products were then purified by flash column chromatography (EtOAc/Hex or MeOH/CH_2Cl_2 as eluents) to yield the desired product.

2.3.3 General Procedure C

Reduction of a aryl nitro group to a primary amine

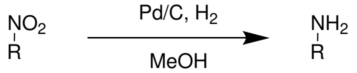

To a solution of nitro compound (1.0 eq.) in MeOH (0.1 M) was added Pd/C (15% w/w, 10 mol%). The resulting solution was sparged with H_2 for 5 minutes and then placed under an atmosphere of H_2 at room temperature. The reaction mixture was allowed to stir for 2-24 hours, or until TLC indicated conversion of starting material. The reaction mixture was then filtered through a pad of Celite© and concentrated *in vacuo* to yield the corresponding amine. Amines were then used without further purification.

2.3.4 General Procedure D

Diazotisation of aryl amines to form triazoles

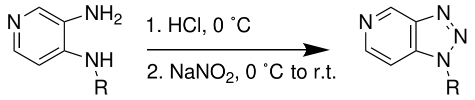

A solution of diamine (1.0 eq) in deionised H_2O (0.15 M) was chilled to 0 °C, treated dropwise with concentrated HCl (32%, 2.2 eq.) and let stir for 15 minutes. $NaNO_2$ (1.25 eq.) was then dissolved in H_2O (4 mL/mmol) and added dropwise to the chilled reaction mixture. The solution was stirred at 0 °C for 15 minutes, before being warmed to room temperature. The reaction mixture was stirred for a further 2-24 hours, or until TLC indicated conversion of starting material. The reaction mixture was then quenched over

ice with NaOH (3.0 M, 10 mL/mL HCl) and subsequently extracted with CH_2Cl_2 (3 × 40 mL/mmol). The organic layers were combined, dried over $MgSO_4$ and concentrated *in vacuo* to yield the desired product. Triazoles were then used without further purification.

2.3.5 General Procedure E

Reduction of triazolopyridines to tetrahydrotriazolopyridines

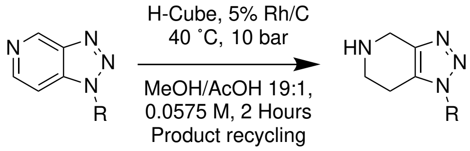

The corresponding triazolopyridine was dissolved in MeOH/AcOH (19:1, 18 mL/mmol) and hydrogenated under 10 bar H_2 pressure at 40 °C using a 5% Rh/C catalyst cart. Hydrogenation was carried out using product recycling for 2 hours, or until completion by TLC, whichever came first. Product was collected and both the solvent and reagent lines were flushed with MeOH/AcOH (19:1, 20 mL/mmol). The solutions were concentrated under a stream of N_2 to yield the crude tetrahydrotriazolopyridine. The crude product was dissolved in a solution of saturated NH_4Cl (20 mL/mmol) and the aqueous phase was washed with CH_2Cl_2 (3 × 40 mL/mmol). The aqueous phase was adjusted to pH 10 with NaOH (1.0 M) and extracted with CH_2Cl_2 (3 × 60 mL/mmol). The organics were combined, dried over $MgSO_4$ and concentrated *in vacuo* to yield the desired product. Tetrahydrotriazolopyridines were then used without further purification.

2.3.6 General Procedure F

HATU mediated amide coupling of carboxylic acids with secondary amines

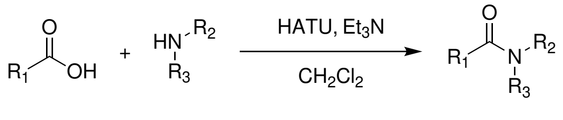

A solution of carboxylic acid (1.0 eq.), Et_3N (3.0 eq.) and HATU (1.3 eq.) was stirred in CH_2Cl_2 (0.5 M) for 30 minutes. The reaction mixture was then treated with the corresponding amine (1.3 eq.) and stirred for a further 15 minutes, or until TLC indicated

conversion of starting material. The reaction mixture was diluted in H_2O (25 mL/mmol) and extracted with CH_2Cl_2 (3 × 25 mL/mmol). The combined organic layers were then washed with H_2O and dried over $MgSO_4$ and concentrated under reduced pressure. The crude product was then purified by flash chromatography (EtOAc/Hex or MeOH/CH_2Cl_2 as eluents) to yield the desired amide.

2.4 Synthesis and Characterisation

2.4.1 Synthesis and characterisation of compounds 1a-o

5-fluoro-2-hydrazineylpyrimidine (**6**)

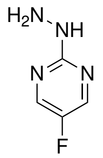

2-chloro-5-fluoropyrimidine (1.871 g, 1 eq.) was dissolved in EtOH (10 mL). The reaction mixture was treated with hydrazine monohydrate (97%, 1.5 mL, 2 eq.) and heated to reflux for 16 hours. The reaction mixture was diluted into H_2O (50 mL) and extracted with CH_2Cl_2 (5 × 100 mL) followed by $CHCl_3$/2-propanol (3:1, 50 mL). The organics were combined, dried over $MgSO_4$, and concentrated *in vacuo* yielding **6** as white crystals (1.503 g, 78%).

^{1}**H NMR** (500 MHz, DMSO−d_6): δ 8.39 (s, 2H), 8.20 (s, 1H), 4.14 (s, 2H) ppm; ^{13}**C NMR** (126 MHz, DMSO−d_6): δ 162.2, 153.8, 150.6, 146.0, 145.7 ppm; ^{19}**F NMR** (471 MHz, DMSO−d_6): δ -156.8 ppm; **LRMS (+ESI) m/z**: 129.1 $[M+H]^+$. Characterisation is consistent with literature.[2]

5-fluoro-2-azidopyrimidine (**7**)

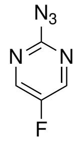

Compound **6** (2.119 g, 1 eq.) was dissolved in CH_3COOH/H_2O (3:1, 66 mL) and chilled to 5 °C. $NaNO_2$ (2.2761 g, 2 eq.) was dissolved in H_2O (15.4 mL) and added to the

solution dropwise. The solution was then stirred over ice for 2 hours and diluted in H_2O (66 mL). The reaction mixture was extracted with Et_2O/pentane (3 × 80 mL) and the combined organics were basified to pH 8 with Na_2CO_3. The layers were separated and the aqueous layer was extracted with Et_2O/pentane (3 × 60 mL). The organic layers were combined, dried over $MgSO_4$, and concentrated over ice to yield **7** as a white solid at 0 °C (1.791 g, 78%). Compound **7** melts at room temperature to form a colourless oil.
^{1}**H NMR** (300 MHz, DMSO−d_6): δ 8.86 (s, 1H) ppm; ^{13}**C NMR** (75 MHz, DMSO−d_6): δ 157.6 (d, J = 2.9 Hz), 155.6 (d, J = 256.2 Hz), 148.0 (d, J = 23.1 Hz) ppm; ^{19}**F NMR** (282 MHz, DMSO−d_6): δ -144.5 ppm; **LRMS (+ESI) m/z**: 152.6 $[M+Na]^+$. Characterisation is consistent with literature.[2]

***tert*-butyl 1-(5-fluoropyrimidin-2-yl)-1,4,6,7-tetrahydro-5*H*-[1,2,3]triazolo[4,5-*c*]pyridine-5-carboxylate** (8)

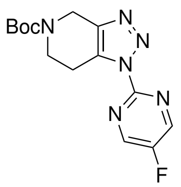

To a solution of *tert*-butyl 4-oxopiperidine-1-carboxylate (1.97 g, 1 eq.) in MePh (77 mL) was added pyrrolidine (1.1 mL, 1 eq.). The solution was stirred at room temperature for 3 hours. The reaction mixture was then treated with a solution of **7** (1.791 g, 1.3 eq.) in MePh (11.5 mL) and heated to reflux for 16 hours. The reaction mixture was then cooled over ice and diluted with CH_2Cl_2 (60 mL), followed by the addition of $NaHCO_3$ (840 mg, 1 eq.) and *m*-CPBA (2.589 g, 1.5 eq.). The reaction mixture was stirred over ice for 30 minutes and then quenched in NaOH (1.0 M, 50 mL). The reaction mixture was extracted with CH_2Cl_2 (3 × 80 mL). The organics were combined, dried over $MgSO_4$ and concentrated *in vacuo*. The crude product was purified by flash column chromatography (0–100% EtOAc/Hex) to yield **8** as a white solid (1.7028 g, 53%).
^{1}**H NMR** (400 MHz, $CDCl_3$): δ 8.73 (s, 2H), 4.72 (s, 2H), 3.78 (t, J = 5.8 Hz, 2H), 3.23 (t, J = 5.7 Hz, 2H), 1.49 (s, 9H) ppm; ^{13}**C NMR** (101 MHz, $CDCl_3$): δ 158.0, 155.3, 154.8, 151.4, 151.4, 147.0, 146.8, 142.5, 132.4, 80.6, 41.5, 28.4, 24.3 ppm; ^{19}**F NMR** (376 MHz, $CDCl_3$): δ −137.9 ppm; **LRMS (+ESI) m/z**: 343.2 $[M+Na]^+$. Characterisation is consistent with literature.[2]

1-(5-fluoropyrimidin-2-yl)-4,5,6,7-tetrahydro-1*H*-[1,2,3]triazolo[4,5-*c*]pyridine (9)

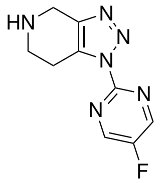

Compound **8** (1.65 g, 1 eq.) was suspended in HCl·1,4-Dioxane (4.0 M, 6.4 mL, 5 eq.) and treated with MeOH (5 mL). The reaction mixture was stirred at room temperature for 16 hours. The reaction mixture was then concentrated under a stream of N_2 before being quenched in NaOH (1.0 M, 40 mL). The aqueous solution was extracted with CH_2Cl_2 (3 × 80 mL), the organic layers combined, dried over $MgSO_4$, and concentrated to yield **9** as a white solid (1.04 g, 91%).
^{1}**H NMR** (400 MHz, DMSO−d_6): δ 9.09 (s, 2H), 3.87 (s, 2H), 3.01 – 2.91 (m, 4H) ppm; ^{13}**C NMR** (101 MHz, DMSO−d_6): δ 158.5, 155.9, 151.4, 151.4, 148.0, 147.8, 143.9, 133.2, 42.7, 42.1, 25.2 ppm; ^{19}**F NMR** (376 MHz, DMSO−d_6): δ −138.9 ppm; **LRMS (+ESI) m/z**: 221.2 $[M+H]^+$; **HRMS (+ESI) m/z**: calc. for $C_9H_{10}FN_6$ $[M+H]^+$: 221.0946, found: 221.0943; Characterisation is consistent with literature.[3]

(adamantan-1-yl)(1-(5-fluoropyrimidin-2-yl)-1,4,6,7-tetrahydro-5*H*-[1,2,3]triazolo[4,5-*c*]pyridin-5-yl)methanone (1a)

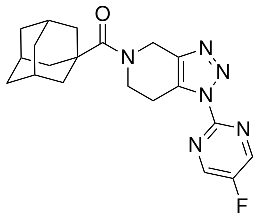

Compound **1a** was prepared according to **General Procedure A** from adamantane carboxylic acid (37.4 mg). The crude product was purified by flash chromatography (0–100% EtOAc/Hex) to yield the title compound **1a** as a white powder (15.8 mg, 21%).
IR (neat, diamond cell): $\tilde{\nu}$ = 1619, 1481, 1438, 1408, 1253, 1234, 1066, 1039, 931 cm^{-1}; ^{1}**H NMR** (500 MHz, $CDCl_3$): δ 8.73 (s, 2H), 4.98 (d, J = 1.5 Hz, 2H), 3.96 (t, J = 5.7 Hz, 2H), 3.25 (tt, J = 5.8, 1.5 Hz, 2H), 2.06 (s, 3H), 2.03 (s, 6H), 1.75 (s, 6H) ppm; ^{13}**C NMR** (126 MHz, $CDCl_3$): δ 176.9, 157.7, 155.6, 151.4, 147.0, 146.8, 142.5, 132.9,

43.5, 42.5, 42.1, 39.0, 36.6, 28.4, 24.6 ppm; ^{19}**F NMR** (471 MHz, $CDCl_3$): δ −137.7 ppm; **LRMS (+ESI) m/z**: 405.2 $[M+Na]^+$; **HRMS (+ESI) m/z**: calc. for $C_{20}H_{23}FN_6NaO$ $[M+Na]^+$: 405.1810, found: 405.1805; **HPLC:** R_T = 24.0 min, 96.9% (254 nm).

2-(adamantan-1-yl)-1-(1-(5-fluoropyrimidin-2-yl)-1,4,6,7-tetrahydro-5*H*-[1,2,3]triazolo[4,5-*c*]pyridin-5-yl)ethan-1-one (1b)

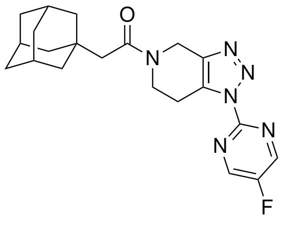

Compound **1b** was prepared according to **General Procedure A** from adamantyl acetic acid (37.4 mg). The crude product was purified by flash chromatography (0–100% EtOAc/Hex) to yield the title compound **1b** as a white powder (33.1 mg, 61%).

IR (neat, diamond cell): $\tilde{\nu}$ = 1631, 1434, 1235, 1139, 1068, 1042, 921 cm^{-1}; ^{1}**H NMR** (500 MHz, $CDCl_3$): δ 8.76 – 8.71 (m, 2H), 4.94 – 4.78 (m, 2H), 4.02 – 3.81 (m, 2H), 3.31 – 3.21 (m, 2H), 2.28 – 2.24 (m, 2H), 2.01 – 1.92 (m, 3H), 1.75 – 1.59 (m, 12H) ppm; ^{13}**C NMR** (126 MHz, $CDCl_3$): δ 170.7, 170.5, 157.8, 157.7, 155.7, 155.6, 151.4, 151.3, 147.0, 147.0, 146.8, 146.8, 142.6, 142.0, 133.3, 131.4, 46.8, 46.5, 44.3, 43.7, 42.9, 42.8, 39.5, 39.0, 36.8, 36.7, 33.8, 33.7, 28.7, 28.7, 25.2, 24.2 ppm; ^{19}**F NMR** (471 MHz, $CDCl_3$): δ −137.6, −137.8 ppm; **LRMS (+ESI) m/z**: 419.2 $[M+Na]^+$; **HRMS (+ESI) m/z**: calc. for $C_{21}H_{25}FN_6NaO$ $[M+Na]^+$: 419.1966, found: 419.1961; **HPLC:** R_T = 24.6 min, 97.7% (254 nm).

3-(adamantan-1-yl)-1-(1-(5-fluoropyrimidin-2-yl)-1,4,6,7-tetrahydro-5*H*-[1,2,3]triazolo[4,5-*c*]pyridin-5-yl)propan-1-one (1c)

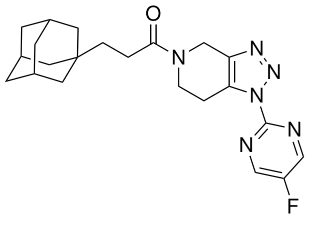

Compound **1c** was prepared according to **General Procedure A** from 3-(1-Adamantyl) propanoic acid (60.3 mg). The crude product was purified by flash chromatography (0–

100% EtOAc/Hex) to yield the title compound **1c** as a white powder (26.8 mg, 30%).

IR (neat, diamond cell): $\tilde{\nu}$ = 1647, 1474, 1421, 1261, 1235, 1042, 926 cm^{-1}; ^{1}**H NMR** (500 MHz, $CDCl_3$): δ 8.73 (d, J = 2.5 Hz, 2H), 4.89 (s, 1H), 4.77 (s, 1H), 3.99 – 3.93 (m, 1H), 3.82 – 3.76 (m, 1H), 3.33 – 3.27 (m, 1H), 3.26 – 3.20 (m, 1H), 2.40 (dt, J = 8.6, 5.6 Hz, 2H), 1.97 (s, 3H), 1.76 – 1.62 (m, 6H), 1.53 – 1.38 (m, 8H) ppm; ^{13}**C NMR** (126 MHz, $CDCl_3$): δ 173.6, 173.1, 157.8, 155.7, 151.4, 147.0, 146.8, 142.6, 141.8, 133.2, 131.4, 43.0, 42.7, 42.2, 39.6, 39.5, 39.3, 39.0, 37.1, 32.0, 28.6, 27.7, 27.3, 25.2, 24.0 ppm; ^{19}**F NMR** (471 MHz, $CDCl_3$): δ −137.6, −137.8 ppm; **LRMS (+ESI) m/z**: 433.2 $[M+Na]^+$; **HRMS (+ESI) m/z**: calc. for $C_{22}H_{27}FN_6NaO$ $[M+Na]^+$: 433.2123, found: 433.2128; **HPLC:** R_T = 25.9 min, 97.5% (254 nm).

(2-chloro-3-(trifluoromethyl)phenyl)(5-fluoropyrimidin-2-yl)-1,4,6,7-tetrahydro-5*H*-[1,2,3]triazolo[4,5-*c*]pyridin-5-yl)propan-1-one (1d)

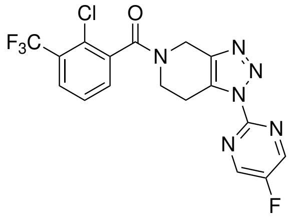

Compound **1d** was prepared according to **General Procedure F** from 2-chloro-3-(trifluoromethyl) benzoic acid (43.7 mg). The crude product was purified by flash chromatography (0–100% EtOAc/Hex) to yield the title compound **1d** as a white powder (34.8 mg, 58%).

^{1}**H NMR** (500 MHz, $CDCl_3$): δ 8.80 – 8.69 (m, 2H), 7.83 – 7.75 (m, 1H), 7.57 – 7.44 (m, 2H), 5.22 – 5.04 (m, 1H), 4.64 – 4.44 (m, 1H), 4.35 – 4.03 (m, 1H), 3.67 – 3.51 (m, 1H), 3.42 – 3.10 (m, 2H) ppm; ^{13}**C NMR** (126 MHz, $CDCl_3$): δ 166.7, 166.6, 157.8, 155.7, 155.7, 155.7, 151.3, 147.1, 147.0, 146.9, 146.9, 141.5, 141.1, 138.3, 137.9, 132.5, 131.2, 131.0, 128.5, 128.4, 127.6, 127.6, 123.5, 121.4, 65.9, 44.0, 43.8, 39.7, 39.2, 24.9, 24.0, 15.3 ppm; ^{19}**F NMR** (471 MHz, $CDCl_3$): δ -62.4, -62.5, −137.3, −137.5 ppm; **LRMS (+ESI) m/z**: 449.0 $[M+Na]^+$; **HPLC:** R_T = 22.0 min, 99.1% (254 nm). Characterisation is consistent with literature.[4]

cyclopropyl(1-(5-fluoropyrimidin-2-yl)-1,4,6,7-tetrahydro-5*H*-[1,2,3]triazolo[4,5-*c*]pyridin-5-yl)methanone (1e)

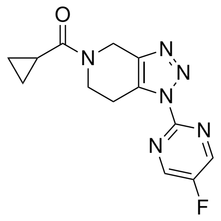

Compound **1e** was prepared according to **General Procedure F** from cyclopropane carboxylic acid (20.6 mg). The crude product was purified by flash chromatography (0–2% MeOH/CH_2Cl_2) to yield the title compound **1e** as a white powder (24.3 mg, 42%).

IR (neat, diamond cell): $\tilde{\nu}$ = 1631, 1463, 1431, 1234, 1198, 1062, 1038, 923 cm^{-1}; ^{1}**H NMR** (500 MHz, $CDCl_3$): δ 8.74 (s, 2H), 4.99 (s, 1H), 4.91 (s, 1H), 3.98 (s, 2H), 3.34 (s, 1H), 3.24 (s, 1H), 1.83 (tt, J = 8.1, 4.6 Hz, 1H), 1.03 (s, 2H), 0.85 (dq, J = 7.1, 3.9 Hz, 2H) ppm; ^{13}**C NMR** (126 MHz, $CDCl_3$): δ 173.2, 157.8, 155.7, 151.4, 147.0, 146.8, 142.6, 141.9, 133.2, 42.7, 41.0, 40.2, 39.5, 25.3, 24.0, 11.8, 11.4, 7.9, 7.6 ppm; ^{19}**F NMR** (471 MHz, $CDCl_3$): δ −137.6, −137.8 ppm; **LRMS (+ESI) m/z**: 311.1 $[M+Na]^+$; **HRMS (+ESI) m/z**: calc. for $C_{13}H_{13}FN_6NaO$ $[M+Na]^+$: 311.1027, found: 311.1021; **HPLC:** R_T = 15.3 min, 97.5% (254 nm).

2-cyclopropyl-1-(1-(5-fluoropyrimidin-2-yl)-1,4,6,7-tetrahydro-5*H*-[1,2,3]triaz-olo[4,5-*c*]pyridin-5-yl)ethan-1-one (1f)

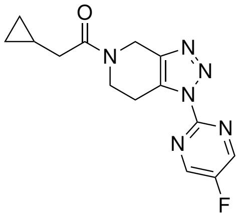

Compound **1f** was prepared according to **General Procedure F** from cyclopropyl acetic acid (0.023 mL). The crude product was purified by flash chromatography (0–100% EtOAc/Hex) to yield the title compound **1f** as a white powder (32.7 mg, 60%).

IR (neat, diamond cell): $\tilde{\nu}$ = 1643, 1575, 1483, 1427, 1278, 1254, 1232, 1204, 1070, 1044, 979, 923, 837 cm^{-1}; ^{1}**H NMR** (500 MHz, $CDCl_3$): δ 8.66 (d, J = 4.2 Hz, 2H), 4.85 (s, 1H), 4.68 (s, 1H), 3.92 (t, J = 5.8 Hz, 1H), 3.72 (t, J = 5.7 Hz, 1H), 3.20 (dt, J = 19.9, 5.9 Hz, 2H), 2.33 (t, J = 7.1 Hz, 2H), 1.03 – 0.99 (m, 1H), 0.58 – 0.47 (m, 2H), 0.18 – 0.10 (m, 2H) ppm; ^{13}**C NMR** (126 MHz, $CDCl_3$): δ 172.2, 171.8, 155.7, 151.4,

147.0, 146.9, 142.5, 141.7, 133.2, 43.0, 42.7, 39.6, 39.1, 39.0, 38.9, 25.2, 24.0, 7.3, 7.1, 4.7, 4.5 ppm; ^{19}**F NMR** (471 MHz, $CDCl_3$): δ −137.5, −137.8 ppm; **LRMS (+ESI) m/z**: 325.1 $[M+Na]^+$; **HRMS (+ESI) m/z**: calc. for $C_{14}H_{15}FN_6NaO$ $[M+Na]^+$: 325.1184 , found: 325.1188; **HPLC:** R_T = 16.3 min, 97.5% (254 nm).

cyclobutyl(1-(5-fluoropyrimidin-2-yl)-1,4,6,7-tetrahydro-5*H*-[1,2,3]triazolo [4,5-*c*]pyridin-5-yl)methanone (1g)

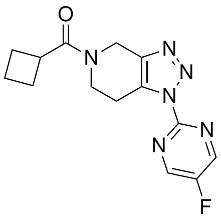

Compound **1g** was prepared according to **General Procedure F** from cyclobutane carboxylic acid (0.025 mL). The crude product was purified by flash chromatography (0–100% EtOAc/Hex) to yield the title compound **1g** as a white powder (24.3 mg, 53%). **IR** (neat, diamond cell): $\tilde{\nu}$ = 1634, 1596, 1479, 1431, 1298, 1258, 1234, 1209, 1083, 1045, 951, 926 cm^{-1}; ^{1}**H NMR** (500 MHz, $CDCl_3$): δ 8.73 (d, J = 4.9 Hz, 2H), 4.89 (t, J = 1.5 Hz, 1H), 4.64 (t, J = 1.5 Hz, 1H), 3.96 (t, J = 5.8 Hz, 1H), 3.68 (t, J = 5.7 Hz, 1H), 3.36 (h, J = 8.6 Hz, 1H), 3.27 – 3.20 (m, 2H), 2.48 – 2.35 (m, 1H), 2.38 – 2.29 (m, 1H), 2.28 – 2.18 (m, 2H), 2.08 – 1.96 (m, 1H), 1.91 – 1.87 (m, 1H) ppm; ^{13}**C NMR** (126 MHz, $CDCl_3$): δ 174.2, 173.8, 157.8, 157.7, 155.7, 155.6, 151.4, 147.0, 147.0, 146.8, 146.8, 142.5, 141.8, 133.1, 131.4, 42.3, 41.9, 39.6, 39.1, 37.7, 37.5, 25.2, 25.2, 25.0, 24.0, 18.1, 17.9 ppm; ^{19}**F NMR** (471 MHz, $CDCl_3$): δ −137.6, −137.8 ppm; **LRMS (+ESI) m/z**: 325.1 $[M+Na]^+$; **HRMS (+ESI) m/z**: calc. for $C_{14}H_{15}FN_6NaO$ $[M+Na]^+$: 325.1184, found: 325.1189; **HPLC:** R_T = 17.0 min, 97.1% (254 nm).

2-cyclobutyl-1-(1-(5-fluoropyrimidin-2-yl)-1,4,6,7-tetrahydro-5*H*-[1,2,3]triazo lo[4,5-*c*]pyridin-5-yl)ethan-1-one (1h)

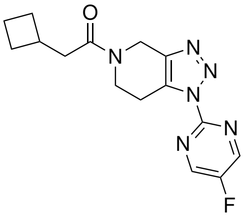

Compound **1h** was prepared according to **General Procedure F** from cyclobutyl acetic acid (0.027 mL). The crude product was purified by flash chromatography (0–100% EtOAc/Hex) to yield the title compound **1h** as a white powder (34.9 mg, 61%).
IR (neat, diamond cell): $\tilde{\nu}$ = 1647, 1482, 1417, 1252, 1235, 1065, 1042, 931 cm^{-1}; ^{1}**H NMR** (500 MHz, $CDCl_3$): δ 8.73 (s, 2H), 4.88 (s, 1H), 4.75 (s, 1H), 3.95 (t, J = 5.8 Hz, 1H), 3.78 (t, J = 5.7 Hz, 1H), 3.29 (t, J = 5.7 Hz, 1H), 3.22 (t, J = 5.8 Hz, 1H), 2.82 – 2.67 (m, 1H), 2.57 (t, J = 7.5 Hz, 2H), 2.22 – 2.10 (m, 2H), 1.97 – 1.79 (m, 2H), 1.77 – 1.66 (m, 2H) ppm; ^{13}**C NMR** (126 MHz, $CDCl_3$): δ 171.7, 171.3, 157.8, 157.7, 155.7, 155.6, 151.4, 147.0, 147.0, 146.9, 146.8, 142.5, 141.8, 133.2, 131.4, 42.9, 42.6, 40.7, 40.4, 39.4, 38.8, 32.4, 32.3, 28.6, 28.5, 25.2, 24.0, 18.7 ppm; ^{19}**F NMR** (471 MHz, $CDCl_3$): δ −137.6, −137.8 ppm; **LRMS (+ESI) m/z**: 339.1 $[M+Na]^+$; **HRMS (+ESI) m/z**: calc. for $C_{15}H_{17}FN_6NaO$ $[M+Na]^+$: 339.1340 , found: 339.1344; **HPLC:** R_T = 18.4 min, 98.6% (254 nm).

cyclopentyl(1-(5-fluoropyrimidin-2-yl)-1,4,6,7-tetrahydro-5*H*-[1,2,3]triazolo[4,5-*c*]pyridin-5-yl)methanone (1i)

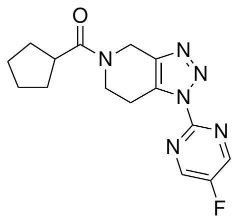

Compound **1i** was prepared according to **General Procedure F** from cyclopentane carboxylic acid (0.026 mL). The crude product was purified by flash chromatography (0–100% EtOAc/Hex) to yield the title compound **1i** as a white powder (25.7 mg, 45%).
IR (neat, diamond cell): $\tilde{\nu}$ = 1624, 1431, 1254, 1232, 1041, 923, 845 cm^{-1}; ^{1}**H NMR** (500 MHz, $CDCl_3$): δ 8.73 (s, 2H), 4.91 (s, 1H), 4.82 (s, 1H), 3.97 (t, J = 5.8 Hz, 1H), 3.85 (t, J = 5.7 Hz, 1H), 3.29 (t, J = 5.9 Hz, 1H), 3.23 (t, J = 5.9 Hz, 1H), 2.99 (p, J = 7.1 Hz, 1H), 1.94 – 1.70 (m, 5H), 1.66 – 1.56 (m, 3H) ppm; ^{13}**C NMR** (126 MHz, $CDCl_3$): δ 175.7, 175.3, 157.8, 155.7, 151.4, 147.0, 146.8, 142.6, 142.0, 133.3, 131.4, 42.9, 42.5, 41.8, 41.6, 39.8, 39.4, 30.3, 30.1, 26.1, 25.4, 24.0 ppm; ^{19}**F NMR** (471 MHz, $CDCl_3$): δ −137.6, −137.8 ppm; **LRMS (+ESI) m/z**: 339.1 $[M+Na]^+$; **HRMS (+ESI) m/z**: calc. for $C_{15}H_{17}FN_6NaO$ $[M+Na]^+$: 339.1340, found: 339.1343; **HPLC:** R_T = 18.3 min,

98.3% (254 nm).

2-cyclopentyl-1-(1-(5-fluoropyrimidin-2-yl)-1,4,6,7-tetrahydro-5*H*-[1,2,3]triazolo[4,5-*c*]pyridin-5-yl)ethan-1-one (1j)

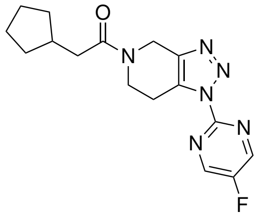

Compound **1j** was prepared according to **General Procedure F** from cyclopentyl acetic acid (0.028 mL). The crude product was purified by flash chromatography (0–100% EtOAc/Hex) to yield the title compound **1j** as a white powder (34.9 mg, 61%).

IR (neat, diamond cell): $\tilde{\nu}$ = 1647, 1481, 1415, 1251, 1227, 1196, 1040, 930 cm^{-1}; ^{1}**H NMR** (500 MHz, $CDCl_3$): δ 8.73 (d, J = 3.1 Hz, 2H), 4.90 (s, 1H), 4.77 (s, 1H), 3.97 (t, J = 5.8 Hz, 1H), 3.80 (t, J = 5.7 Hz, 1H), 3.29 (t, J = 5.7 Hz, 1H), 3.23 (t, J = 5.9 Hz, 1H), 2.47 (t, J = 7.0 Hz, 2H), 2.36 – 2.19 (m, 1H), 1.94 – 1.80 (m, 1H), 1.71 – 1.50 (m, 4H), 1.24 – 1.12 (m, 3H) ppm; ^{13}**C NMR** (126 MHz, $CDCl_3$): δ 172.3, 171.9, 157.8, 157.7, 155.7, 155.6, 151.4, 147.0, 147.0, 146.9, 146.8, 142.6, 141.8, 133.2, 131.4, 43.0, 42.7, 39.9, 39.6, 39.5, 38.9, 36.6, 36.6, 32.8, 32.7, 25.2, 25.0, 25.0, 24.0 ppm; ^{19}**F NMR** (471 MHz, $CDCl_3$): δ −137.6, −137.8 ppm; **LRMS (+ESI) m/z**: 353.1 $[M+Na]^{+}$; **HRMS (+ESI) m/z**: calc. for $C_{16}H_{19}FN_6NaO$ $[M+Na]^{+}$: 353.1497 , found: 353.1499; **HPLC:** R_T = 19.8 min, 98.6% (254 nm).

cyclohexyl(1-(5-fluoropyrimidin-2-yl)-1,4,6,7-tetrahydro-5*H*-[1,2,3]triazolo [4,5-*c*]pyridin-5-yl)methanone (1k)

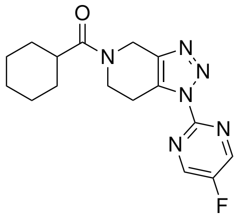

Compound **1k** was prepared according to **General Procedure F** from cyclohexane carboxylic acid (0.027 mL). The crude product was purified by flash chromatography (0–100% EtOAc/Hex) to yield the title compound **1k** as a white powder (41.2 mg, 70%). **IR** (neat, diamond cell): $\tilde{\nu}$ = 1626, 1429, 1258, 1231, 1210, 1040, 919, 842 cm^{-1}; ^{1}**H NMR** (500 MHz, $CDCl_3$): δ 8.73 (s, 2H), 4.89 (s, 1H), 4.80 (s, 1H), 3.99 – 3.93 (m, 1H), 3.82 (s, 1H), 3.31 (s, 1H), 3.25 – 3.19 (m, 1H), 2.56 (tt, J = 11.7, 3.4 Hz, 1H), 1.88 – 1.44 (m, 7H), 1.36 – 1.25 (m, 3H) ppm; ^{13}**C NMR** (126 MHz, $CDCl_3$): δ 175.6, 157.8, 155.7, 151.4, 147.0, 146.8, 142.6, 142.0, 133.3, 131.4, 77.3, 42.8, 42.4, 41.3, 41.2, 39.6, 39.2, 29.6, 29.3, 25.8, 25.6, 24.0 ppm; ^{19}**F NMR** (471 MHz, $CDCl_3$): δ −137.6, −137.8 ppm; **LRMS (+ESI) m/z**: 353.1 $[M+Na]^+$; **HRMS (+ESI) m/z**: calc. for $C_{16}H_{19}FN_6NaO$ $[M+Na]^+$: 353.1497, found: 353.1500; **HPLC:** R_T = 19.5 min, 99.0% (254 nm).

2-cyclohexyl-1-(1-(5-fluoropyrimidin-2-yl)-1,4,6,7-tetrahydro-5*H*-[1,2,3]triazo lo[4,5-*c*]pyridin-5-yl)ethan-1-one (1l)

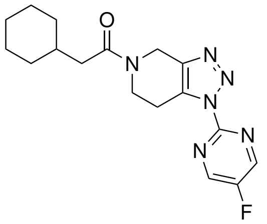

Compound **1l** was prepared according to **General Procedure F** from cyclohexyl acetic acid (32.1 mg). The crude product was purified by flash chromatography (0–100% EtOAc/Hex) to yield the title compound **1l** as a white powder (32.1 mg, 51%).

IR (neat, diamond cell): $\tilde{\nu}$ = 1638, 1578, 1435, 1257, 1235, 1191, 1065, 027, 843 cm^{-1}; ^{1}**H NMR** (500 MHz, $CDCl_3$): δ 8.73 (d, J = 2.5 Hz, 2H), 4.91 (s, 1H), 4.77 (s, 1H), 3.97 (t, J = 5.8 Hz, 1H), 3.80 (t, J = 5.7 Hz, 1H), 3.26 (dt, J = 23.0, 5.8 Hz, 2H), 2.32 (t, J

= 6.3 Hz, 2H), 1.88 – 1.63 (m, 6H), 1.35 – 1.21 (m, 2H), 1.14 (dtt, J = 12.6, 7.1, 3.0 Hz, 1H), 1.05 – 0.93 (m, 2H) ppm; ^{13}**C NMR** (126 MHz, $CDCl_3$): δ 172.0, 171.6, 157.8, 157.7, 155.7, 155.6, 151.4, 147.0, 147.0, 146.8, 146.8, 142.5, 141.8, 133.2, 131.4, 43.2, 42.8, 41.4, 41.1, 39.6, 39.0, 35.1, 33.5, 33.4, 26.2, 26.2, 26.1, 26.1, 25.3, 24.1 ppm; ^{19}**F NMR** (471 MHz, $CDCl_3$): δ −137.6, −137.8 ppm; **LRMS (+ESI) m/z**: 367.2 $[M+Na]^+$; **HRMS (+ESI) m/z**: calc. for $C_{17}H_{21}FN_6NaO$ $[M+Na]^+$: 367.1653, found: 367.1650; **HPLC:** R_T = 21.1 min, 98.8% (254 nm).

bicyclo[1.1.1]pentan-1-yl(1-(5-fluoropyrimidin-2-yl)-1,4,6,7-tetrahydro-5*H*-[1,2,3]triazolo[4,5-*c*]pyridin-5-yl)methanone (1m)

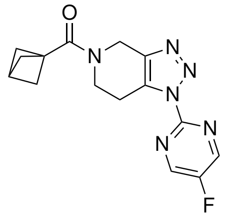

Compound **1m** was prepared according to **General Procedure F** from bicyclo[1.1.1]-pentane–1–carboxylic acid (36.3 mg). The crude product was purified by flash chromatography (0–100% EtOAc/Hex) to yield the title compound **1m** as a white powder (35.2 mg, 39%).

IR (neat, diamond cell): $\tilde{\nu}$ = 1622, 1432, 1302, 1219, 1088, 1042, 922 cm^{-1}; ^{1}**H NMR** (500 MHz, $CDCl_3$): δ 8.74 (s, 2H), 4.92 (s, 1H), 4.86 (s, 1H), 3.94 (dt, J = 11.6, 5.7 Hz, 2H), 3.29 – 3.21 (m, 2H), 2.54 (d, J = 9.8 Hz, 1H), 2.24 (d, J = 8.6 Hz, 6H) ppm; ^{13}**C NMR** (126 MHz, $CDCl_3$): δ 169.1, 168.7, 157.8, 157.7, 155.7, 155.6, 151.3, 147.0, 147.0, 146.9, 146.8, 142.1, 141.7, 133.0, 131.4, 53.0, 52.8, 45.1, 45.0, 42.8, 42.3, 39.9, 39.3, 28.9, 28.8, 25.3, 23.9 ppm; ^{19}**F NMR** (471 MHz, $CDCl_3$): δ −137.5, −137.8 ppm; **LRMS (+ESI) m/z**: 315.2 $[M+H^+]$; **HRMS (+ESI) m/z**: calc. for $C_{15}H_{15}FN_6NaO$ $[M+Na]^+$: 337.1184, found: 337.1182; **HPLC:** R_T = 17.2 min, 96.5% (254 nm).

cuban-1-yl(1-(5-fluoropyrimidin-2-yl)-1,4,6,7-tetrahydro-5*H*-[1,2,3]triazolo[4,5-*c*]pyridin-5-yl)methanone (1n)

Compound **1n** was prepared according to **General Procedure F** from cubane–1–carboxylic acid (35.6 mg). The crude product was purified by flash chromatography (0–100% EtOAc/Hex) to yield the title compound **1n** as a white powder (36.0 mg, 57%).

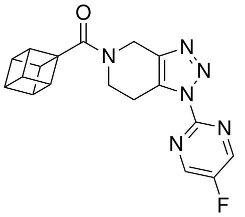

IR (neat, diamond cell): $\tilde{\nu}$ = 1627, 1416, 1253, 1225, 1069, 1037, 921 cm^{-1}; ^{1}**H NMR** (500 MHz, $CDCl_3$): δ 8.74 (d, J = 4.4 Hz, 2H), 4.87 (s, 1H), 4.57 (s, 1H), 4.37 – 4.25 (m, 3H), 4.11 – 3.99 (m, 4H), 3.95 (t, J = 5.8 Hz, 1H), 3.59 (t, J = 5.7 Hz, 1H), 3.30 (t, J = 5.8 Hz, 1H), 3.24 (t, J = 5.8 Hz, 1H) ppm; ^{13}**C NMR** (126 MHz, $CDCl_3$): δ 171.5, 171.2, 157.8, 157.7, 155.7, 155.6, 151.4, 147.0, 147.0, 146.9, 146.8, 142.2, 141.8, 133.1, 131.3, 57.9, 57.9, 49.5, 49.3, 46.9, 46.8, 44.6, 44.6, 42.2, 41.6, 39.6, 39.2, 25.5, 24.0 ppm; ^{19}**F NMR** (471 MHz, $CDCl_3$): δ −137.5, −137.8 ppm; **LRMS (+ESI) m/z**: 373.1 $[M+Na]^+$; **HRMS (+ESI) m/z**: calc. for $C_{18}H_{15}FN_6NaO$ $[M+Na]^+$: 373.1184, found: 373.1179; **HPLC:** R_T = 19.6 min, 98.4% (254 nm).

bicyclo[2.2.2]octan-1-yl(1-(5-fluoropyrimidin-2-yl)-1,4,6,7-tetrahydro-5*H*-[1,2,3]triazolo[4,5-*c*]pyridin-5-yl)methanone (1o)

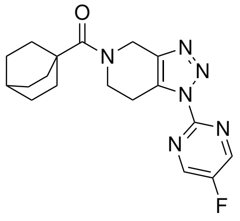

Compound **1o** was prepared according to **General Procedure F** from bicyclo[2.2.2]-octane–1–carboxylic acid (39.7 mg). The crude product was purified by flash chromatography (0–100% EtOAc/Hex) to yield the title compound **1o** as a white powder (42.7 mg, 65%).

IR (neat, diamond cell): $\tilde{\nu}$ = 1639, 1594, 1485, 1428, 1297, 1258, 1234, 1209, 1083, 1045, 951, 926, 844 cm^{-1}; ^{1}**H NMR** (500 MHz, $CDCl_3$): δ 8.73 (s, 2H), 4.94 (d, J = 1.5 Hz, 2H), 3.94 (t, J = 5.7 Hz, 2H), 3.23 (tt, J = 5.8, 1.5 Hz, 2H), 1.90 – 1.83 (m, 6H), 1.68 (hept, J = 3.0 Hz, 1H), 1.64 – 1.59 (m, 6H) ppm; ^{13}**C NMR** (126 MHz, $CDCl_3$): δ 177.1, 157.7, 155.6, 151.4, 151.4, 147.0, 146.8, 142.4, 132.9, 43.6, 42.3, 39.6, 28.2, 25.5, 24.4, 23.8 ppm; ^{19}**F NMR** (471 MHz, $CDCl_3$): δ −137.6, −137.8 ppm; **LRMS (+ESI) m/z**: 379.3 $[M+Na]^+$; **HRMS (+ESI) m/z**: calc. for $C_{18}H_{21}FN_6NaO$ $[M+Na]^+$: 379.1653, found: 379.1653; **HPLC:** R_T = 21.2 min, 98.9% (254 nm).

2.4.2 Synthesis and characterisation of compounds 2a-g

***N*^4^-(adamantan-1-yl)-3-nitropyridin-4-amine (10a)**

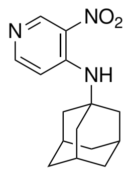

Compound **10a** was prepared according to **General Procedure B** from adamantan-1-amine (1.105 g). The crude product was purified by flash chromatography (0-50% EtOAc/Hex) to yield the title compound **10a** as a yellow powder (1.21 g, 94%).

IR (neat, diamond cell): $\tilde{\nu}$ = 1647, 1474, 1421, 1261, 1235, 1042, 926, 781, 634 cm^{-1}; ^{1}**H NMR** (300 MHz, $CDCl_3$): δ 9.20 (s, 1H), 8.38 (s, 1H), 8.18 (d, J = 6.3 Hz, 1H), 7.02 (d, J = 6.3 Hz, 1H), 2.21 (t, J = 3.2 Hz, 3H), 2.11 (d, J = 2.9 Hz, 6H), 1.77 (d, J = 3.3 Hz, 6H) ppm; ^{13}**C NMR** (75 MHz, $CDCl_3$): δ 151.9, 149.9, 147.8, 130.2, 110.2, 53.5, 41.9, 36.1, 29.4 ppm; **LRMS (+ESI) m/z**: 274.2 $[M+H]^+$; **HRMS (+ESI) m/z**: calc. for $C_{15}H_{20}N_3O_2$ $[M+H]^+$: 274.1550, found: 274.1548.

***N*^4^-((adamantan-1-yl)methyl)-3-nitropyridin-4-amine (10b)**

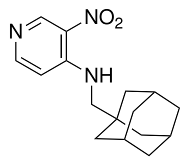

Compound **10b** was prepared according to **General Procedure B** from adamantan-1-yl)methanamine (1.26 mL). The crude product was purified by flash chromatography (0-50% EtOAc/Hex) to yield the title compound **10b** as a yellow powder (1.17 g, 93%).

IR (neat, diamond cell): $\tilde{\nu}$ = 3375, 2897, 2845, 1625, 1564, 1519, 1407, 1364, 1244, 1190, 1116, 1036, 879, 825 cm^{-1}; ^{1}**H NMR** (500 MHz, $CDCl_3$): δ 9.20 (s, 1H), 8.37 (s, 1H), 8.25 (d, J = 6.3 Hz, 1H), 6.74 (d, J = 6.2 Hz, 1H), 3.01 (d, J = 5.7 Hz, 2H), 2.08 – 2.02 (m, 3H), 1.80 – 1.66 (m, 6H), 1.63 – 1.60 (m, 6H) ppm; ^{13}**C NMR** (126 MHz, $CDCl_3$): δ 152.9, 149.3, 149.2, 129.7, 107.9, 54.7, 40.5, 36.7, 33.9, 28.1 ppm; **LRMS (+ESI) m/z**: 288.2 $[M+H]^+$; **HRMS (+ESI) m/z**: calc. for $C_{16}H_{21}N_3NaO_2$ $[M+Na]^+$: 310.1526, found: 310.1524.

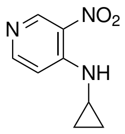

N^4**-cyclopropyl-3-nitropyridin-4-amine (10c)**

Compound **10c** was prepared according to **General Procedure B** from cyclopropanamine (500.4 mg). The crude product was purified by flash chromatography (0-50% EtOAc/Hex) to yield the title compound **10c** as a yellow powder (426.1 mg, 75%).

IR (neat, diamond cell): $\tilde{\nu}$ = 3367, 1608, 1559, 1512, 1403, 1362, 1240, 1194, 1038, 879, 845, 817 cm^{-1}; ^{1}**H NMR** (500 MHz, $CDCl_3$): δ 9.20 (s, 1H), 8.35 (d, J = 6.1 Hz, 1H), 8.18 (s, 1H), 7.15 (d, J = 6.1 Hz, 1H), 2.63 (ttd, J = 6.9, 3.7, 2.0 Hz, 1H), 1.06 – 0.95 (m, 2H), 0.75 – 0.68 (m, 2H) ppm; ^{13}**C NMR** (126 MHz, $CDCl_3$): δ 153.0, 149.9, 148.8, 130.0, 109.3, 24.3, 7.8 ppm; **LRMS (+ESI) m/z**: 180.1 $[M+H]^+$; **HRMS (+ESI) m/z**: calc. for $C_8H_9N_3NaO_2$ $[M+Na]^+$: 202.0587, found: 202.0588.

N^4**-(cyclopropylmethyl)-3-nitropyridin-4-amine (10d)**

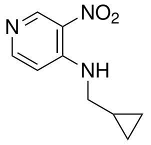

Compound **10d** was prepared according to **General Procedure B** from cyclopropylmethananamine (343.9 mg). The crude product was purified by flash chromatography (0-50% EtOAc/Hex) to yield the title compound **10d** as a crystalline yellow solid (527.7 mg, 87%).

IR (neat, diamond cell): $\tilde{\nu}$ = 3270, 2905, 2842, 1610, 1556, 1516, 1390, 1367, 1343, 1239, 1187, 1034, 864, 813 cm^{-1}; ^{1}**H NMR** (500 MHz, $CDCl_3$): δ 9.21 (s, 1H), 8.28 (d, J = 6.1 Hz, 1H), 8.23 (s, 1H), 6.68 (d, J = 6.1 Hz, 1H), 3.19 (dd, J = 7.0, 5.0 Hz, 2H), 1.24 – 1.13 (m, 1H), 0.73 – 0.65 (m, 2H), 0.38 – 0.32 (m, 2H) ppm; ^{13}**C NMR** (126 MHz, $CDCl_3$): δ 153.1, 149.1, 148.5, 129.7, 107.8, 47.8, 10.1, 3.9 ppm; **LRMS (+ESI) m/z**: 194.2 $[M+H]^+$; **HRMS (+ESI) m/z**: calc. for $C_9H_{11}N_3NaO_2$ $[M+Na]^+$: 216.0744, found: 216.0742.

N^4**-cyclohexyl-3-nitropyridin-4-amine (10e)**

Compound **10e** was prepared according to **General Procedure B** from cyclohexanamine

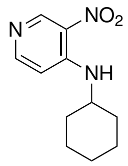

(505.8 mg). The crude product was purified by flash chromatography (0-50% EtOAc/Hex) to yield the title compound **10e** as a yellow powder (627.4 mg, 90%).

IR (neat, diamond cell): $\tilde{\nu}$ = 3368, 2931, 2856, 1615, 1560, 1511, 1401, 1363, 1237, 1194, 1095, 1034, 876, 820 cm^{-1}; ^{1}**H NMR** (500 MHz, $CDCl_3$): δ 9.20 (s, 1H), 8.25 (d, J = 6.2 Hz, 1H), 8.21 – 8.16 (m, 1H), 6.71 (d, J = 6.2 Hz, 1H), 3.59 – 3.49 (m, 1H), 2.11 – 2.01 (m, 2H), 1.87 – 1.77 (m, 2H), 1.73 – 1.65 (m, 1H), 1.49 – 1.29 (m, 5H) ppm; ^{13}**C NMR** (126 MHz, $CDCl_3$): δ 152.9, 149.5, 147.7, 129.6, 108.0, 51.2, 32.4, 25.3, 24.4 ppm; **LRMS (+ESI) m/z**: 222.2 $[M+H]^+$; **HRMS (+ESI) m/z**: calc. for $C_{11}H_{15}N_3NaO_2$ $[M+Na]^+$: 244.1057, found: 244.1052.

***N*^{4}-(cyclohexylmethyl)-3-nitropyridin-4-amine (10f)**

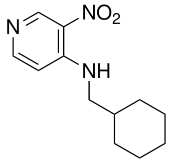

Compound **10f** was prepared according to **General Procedure B** from cyclohexylmet h-anamine (547.4 mg). The crude product was purified by flash chromatography (0-50% EtOAc/Hex) to yield the title compound **10f** as a yellow powder (690.2 mg, 93%).

IR (neat, diamond cell): $\tilde{\nu}$ = 3274, 2917, 2850, 1610, 1560, 1503, 1466, 1401, 1355, 1237, 1216, 1114, 1035, 953, 866 cm^{-1}; ^{1}**H NMR** (500 MHz, $CDCl_3$): δ 9.20 (s, 1H), 8.27 (d, J = 6.2 Hz, 1H), 8.26 – 8.23 (m, 1H), 6.70 (d, J = 6.2 Hz, 1H), 3.17 (s, 2H), 1.88 – 1.63 (m, 5H), 1.36 – 1.15 (m, 3H), 1.11 – 1.00 (m, 2H) ppm; ^{13}**C NMR** (126 MHz, $CDCl_3$): δ 153.0, 149.2, 148.9, 129.7, 107.8, 49.2, 37.3, 31.0, 26.2, 25.7 ppm; **LRMS (+ESI) m/z**: 236.2 $[M+H]^+$; **HRMS (+ESI) m/z**: calc. for $C_{12}H_{17}N_3NaO_2$ $[M+Na]^+$: 258.1213, found: 258.1214.

N^4**-(adamantan-1-yl)-pyridine-3,4-diamine (11a)**

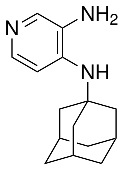

Compound **11a** was prepared according to **General Procedure C** from compound **10a** (462.0 mg). The title compound **11a** was yielded as a white powder (398.4 mg, 96%). **IR** (neat, diamond cell): $\tilde{\nu}$ = 2903, 2848, 1585, 1513, 1357, 1287, 1196, 1096, 866, 805 cm^{-1}; ^{1}**H NMR** (500 MHz, $CDCl_3$): δ 7.66 (s, 1H), 7.54 (d, J = 5.4 Hz, 1H), 6.68 (d, J = 5.4 Hz, 1H), 4.60 (s, 2H), 4.46 (s, 1H), 2.09 (s, 3H), 1.96 (d, J = 2.9 Hz, 6H), 1.69 (d, J = 3.2 Hz, 6H) ppm; ^{13}**C NMR** (126 MHz, $CDCl_3$): δ 143.9, 143.5, 139.0, 128.7, 107.9, 51.7, 42.3, 35.1, 29.6 ppm; **LRMS (+ESI) m/z**: 244.3 $[M+H]^+$; **HRMS (+ESI) m/z**: calc. for $C_{15}H_{22}N_3$ $[M+Na]^+$: 244.1808, found: 244.1805.

N^4**-((adamantan-1-yl)methyl)pyridine-3,4-diamine (11b)**

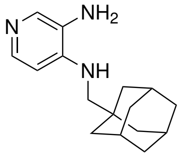

Compound **11b** was prepared according to **General Procedure C** from compound **10b** (752.4 mg). The title compound **11b** was yielded as a yellow powder (599.1 mg, 90%). **IR** (neat, diamond cell): $\tilde{\nu}$ = 3375, 2896, 2844, 1595, 1526, 1450, 1345, 1277, 1065, 856, 801 cm^{-1}; ^{1}**H NMR** (500 MHz, $CDCl_3$): δ 7.95 (d, J = 5.5 Hz, 1H), 7.88 (s, 1H), 6.48 (d, J = 5.4 Hz, 1H), 2.83 (s, 2H), 2.04 – 1.98 (m, 3H), 1.79 – 1.62 (m, 6H), 1.60 – 1.58 (m, 6H) ppm; ^{13}**C NMR** (126 MHz, $CDCl_3$): δ 146.1, 144.4, 138.5, 127.9, 104.7, 54.8, 40.7, 37.0, 33.9, 28.3 ppm; **LRMS (+ESI) m/z**: 258.2 $[M+H]^+$; **HRMS (+ESI) m/z**: calc. for $C_{16}H_{23}N_3$ $[M+H]^+$: 258.1965, found: 258.1962.

N^4**-cyclopropylpyridine-3,4-diamine (11c)**

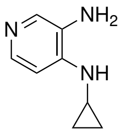

Compound **11c** was prepared according to **General Procedure C** from compound **10c** (385.1 mg). The title compound **11c** was yielded as a white powder (301.1 mg, 94%). **IR** (neat, diamond cell): $\tilde{\nu}$ = 3419, 3231, 1650, 1589, 1519, 1440, 1347, 1284, 1254, 1188, 1064, 1023, 876, 813 cm^{-1}; ^{1}**H NMR** (500 MHz, $CDCl_3$): δ 8.08 – 7.98 (m, 1H), 7.89 (s, 1H), 6.87 (d, J = 5.3 Hz, 1H), 4.56 (s, 1H), 3.04 (s, 2H), 2.51 – 2.43 (m, 1H), 0.87 – 0.72 (m, 2H), 0.63 – 0.53 (m, 2H) ppm; ^{13}**C NMR** (126 MHz, $CDCl_3$): δ 143.9, 143.5, 139.0, 128.7, 107.9, 51.7, 42.3, 35.1, 29.6 ppm; **LRMS (+ESI) m/z**: 150.2 $[M+H]^+$; **HRMS (+ESI) m/z**: calc. for $C_8H_{12}N_3$ $[M+H]^+$: 150.1026, found: 150.1025.

***N*^{4}-(cyclopropylmethyl)pyridine-3,4-diamine (11d)**

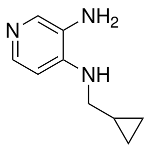

Compound **11d** was prepared according to **General Procedure C** from compound **10d** (540.9 mg). The title compound **11d** was yielded as a white powder (408.3 mg, 89%). **IR** (neat, diamond cell): $\tilde{\nu}$ = 3240, 1592, 1521, 1436, 1357, 1277, 1194, 1068, 1020, 867 cm^{-1}; ^{1}**H NMR** (500 MHz, $CDCl_3$): δ 7.96 (d, J = 5.4 Hz, 1H), 7.90 (s, 1H), 6.44 (d, J = 5.4 Hz, 1H), 4.26 (s, 1H), 3.17 – 3.10 (m, 2H), 3.03 – 2.97 (m, 2H), 1.12 (s, 1H), 0.64 – 0.52 (m, 2H), 0.27 (s, 2H) ppm; ^{13}**C NMR** (126 MHz, $CDCl_3$): δ 144.9, 144.1, 138.0, 128.3, 104.9, 47.9, 10.5, 3.6 ppm; **LRMS (+ESI) m/z**: 164.2 $[M+H]^+$; **HRMS (+ESI) m/z**: calc. for $C_9H_{14}N_3$ $[M+H]^+$: 164.1182, found: 164.1182.

***N*^{4}-cyclohexylpyridine-3,4-diamine (11e)**

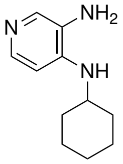

Compound **11e** was prepared according to **General Procedure C** from compound **10e** (627.4 mg). The title compound **11e** was yielded as a white powder (301.1 mg, 94%). **IR** (neat, diamond cell): $\tilde{\nu}$ = 3330, 2931, 2852, 1595, 1522, 1445, 1328, 1276, 1187, 1101, 1068, 903, 804 cm^{-1}; ^{1}**H NMR** (500 MHz, $CDCl_3$): δ 7.94 (d, J = 5.5 Hz, 1H), 7.88 (s, 1H), 6.46 (d, J = 5.5 Hz, 1H), 4.08 (d, J = 7.4 Hz, 1H), 3.36 – 3.25 (m, 1H), 2.98 –

2.89 (br-s, 2H), 2.10 – 2.01 (m, 2H), 1.83 – 1.74 (m, 2H), 1.68 (dt, J = 13.1, 3.9 Hz, 1H), 1.46 – 1.34 (m, 2H), 1.31 – 1.17 (m, 3H) ppm; ^{13}**C NMR** (126 MHz, $CDCl_3$): δ 144.3, 144.1, 138.5, 128.1, 105.0, 65.9, 50.9, 33.1, 25.8, 24.9, 15.3 ppm; **LRMS (+ESI) m/z**: 192.2 $[M+H]^+$; **HRMS (+ESI) m/z**: calc. for $C_{11}H_{18}N_3$ $[M+H]^+$: 192.1495, found: 192.1492.

***N*^{4}-(cyclohexylmethyl)pyridine-3,4-diamine (11f)**

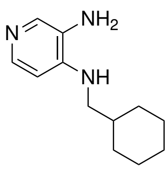

Compound **11f** was prepared according to **General Procedure C** from compound **10f** (640.2 mg). The title compound **11f** was yielded as a white powder (522.3 mg, 93%).

IR (neat, diamond cell): $\tilde{\nu}$ = 3347, 2921, 2845, 1588, 1521, 1465, 1446, 1342, 1287, 1189, 1066, 893, 873 cm^{-1}; ^{1}**H NMR** (500 MHz, $CDCl_3$): δ 7.96 (d, J = 5.4 Hz, 1H), 7.88 (s, 1H), 6.45 (d, J = 5.4 Hz, 1H), 4.26 – 4.21 (m, 1H), 3.07 – 3.01 (m, 2H), 3.01 – 2.97 (m, 2H), 1.86 – 1.66 (m, 5H), 1.66 – 1.56 (m, 1H), 1.32 – 1.16 (m, 3H), 1.06 – 0.95 (m, 2H) ppm; ^{13}**C NMR** (126 MHz, $CDCl_3$): δ 145.4, 144.2, 138.2, 128.1, 104.8, 49.4, 37.4, 31.3, 26.5, 25.9 ppm; **LRMS (+ESI) m/z**: 206.2 $[M+H]^+$; **HRMS (+ESI) m/z**: calc. for $C_{12}H_{20}N_3$ $[M+H]^+$: 206.1651, found: 206.1653.

1-(adamantan-1-yl)-1*H*-[1,2,3]triazolo[4,5-*c*]pyridine (12a)

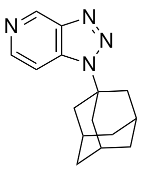

Compound **12a** was prepared according to **General Procedure D** from compound **11a** (168.8 mg). The title compound **12a** was yielded as a white powder (159.3 mg, 91%). **IR** (neat, diamond cell): $\tilde{\nu}$ = 2910, 2850, 1595, 1468, 1357, 1306, 1191, 1135, 1100, 1033, 981, 941, 921, 815 cm^{-1}; ^{1}**H NMR** (500 MHz, $CDCl_3$): δ 9.47 (d, J = 1.2 Hz, 1H), 8.48 (d, J = 6.0 Hz, 1H), 7.68 (dd, J = 6.0, 1.2 Hz, 1H), 2.51 (d, J = 2.9 Hz, 6H), 2.40-2.31 (m, 3 H), 1.88 (t, J = 3.1 Hz, 6H) ppm; ^{13}**C NMR** (126 MHz, $CDCl_3$): δ 145.4, 144.1, 143.7, 135.0, 106.9, 62.3, 42.3, 36.0, 29.6 ppm; **LRMS (+ESI) m/z**: 255.1 $[M+H]^+$; **HRMS (+ESI) m/z**: calc. for $C_{15}H_{18}NaN_4$ $[M+Na]^+$: 206.1651, found: 206.1653.

1-((adamantan-1-yl)methyl)-1*H*-[1,2,3]triazolo[4,5-*c*]pyridine (**12b**)

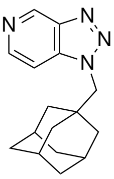

Compound **12b** was prepared according to **General Procedure D** from compound **11b** (272.1 mg). The title compound **12b** was yielded as a white powder (240.7 mg, 77%). **IR** (neat, diamond cell): $\tilde{\nu}$ = 1604, 1583, 1472, 1435, 1315, 1278, 1253, 1214, 1183, 1107, 1074, 1039 ,1021, 989, 927, 812 cm^{-1}; ^{1}**H NMR** (500 MHz, $CDCl_3$): δ 9.47 (s, 1H), 8.56 (d, J = 5.9 Hz, 1H), 7.45 (d, J = 5.9 Hz, 1H), 4.31 (s, 2H), 2.03 – 1.98 (m, 3H), 1.74 – 1.56 (m, 12H) ppm; ^{13}**C NMR** (126 MHz, $CDCl_3$): δ 144.8, 144.6, 142.9, 137.7, 104.9, 60.2, 40.7, 36.5, 35.6, 28.1 ppm; **LRMS (+ESI) m/z**: 269.2 $[M+H]^+$; **HRMS (+ESI) m/z**: calc. for $C_{16}H_{20}NaN_4$ $[M+Na]^+$: 291.1580, found: 291.1577.

1-cyclopropyl-1*H*-[1,2,3]triazolo[4,5-*c*]pyridine (12c)

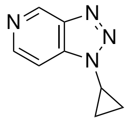

Compound **12c** was prepared according to **General Procedure D** from compound **11c** (272.1 mg). The title compound **12c** was yielded as a white powder (262.2 mg, 89%). **IR** (neat, diamond cell): $\tilde{\nu}$ = 1602, 1482, 1434, 1365, 1278, 1195, 1098, 1022, 946, 926, 876, 824 cm^{-1}; ^{1}**H NMR** (500 MHz, $CDCl_3$): δ 9.45 (d, J = 1.2 Hz, 1H), 8.58 (d, J = 5.8 Hz, 1H), 7.59 (dd, J = 5.9, 1.2 Hz, 1H), 3.85 – 3.78 (m, 1H), 1.43 – 1.33 (m, 4H) ppm; ^{13}**C NMR** (126 MHz, $CDCl_3$): δ 144.8, 144.8, 143.3, 137.7, 104.6, 28.7, 6.4 ppm; **LRMS (+ESI) m/z**: 161.1 $[M+H]^+$; **HRMS (+ESI) m/z**: calc. for $C_8H_8NaN_4$ $[M+Na]^+$: 183.0641, found: 183.0640.

1-(cyclopropylmethyl)-1*H*-[1,2,3]triazolo[4,5-*c*]pyridine (12d)

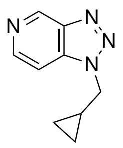

Compound **12d** was prepared according to **General Procedure D** from compound **11d** (396.1 mg). The title compound **12d** was yielded as a pale yellow oil (359.3 mg, 85%). **IR** (neat, diamond cell): $\tilde{\nu}$ = 1604, 1433, 1327, 1264, 1219, 1176, 1070, 1045, 1015, 984, 914, 823 cm^{-1}; ^{1}**H NMR** (500 MHz, $CDCl_3$): δ 9.48 (s, 1H), 8.58 (d, J = 5.8 Hz, 1H), 7.52 (d, J = 5.9 Hz, 1H), 4.55 (d, J = 7.1 Hz, 2H), 1.47 – 1.36 (m, 1H), 0.74 – 0.69 (m, 2H), 0.55 – 0.49 (m, 2H) ppm; ^{13}**C NMR** (126 MHz, $CDCl_3$): δ 144.9, 144.6, 143.4, 136.3, 104.4, 53.3, 11.0, 4.5 ppm; **LRMS (+ESI) m/z**: 197.2 $[M+Na]^+$; **HRMS (+ESI) m/z**: calc. for $C_9H_{10}NaN_4$ $[M+Na]^+$: 197.0798, found: 197.0800.

1-cyclohexyl-1*H*-[1,2,3]triazolo[4,5-*c*]pyridine (12e)

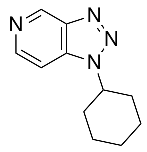

Compound **12e** was prepared according to **General Procedure D** from compound **11e** (272.1 mg). The title compound **12e** was yielded as a white powder (262.2 mg, 89%). **IR** (neat, diamond cell): $\tilde{\nu}$ = 1601, 1579, 1471, 1434, 1328, 1272, 1221, 1179, 1137, 1048, 1018, 997, 926, 894, 811 cm^{-1}; ^{1}**H NMR** (500 MHz, $CDCl_3$): δ 9.47 (s, 1H), 8.54 (d, J = 5.9 Hz, 1H), 7.51 (dd, J = 5.9, 1.3 Hz, 1H), 4.75 – 4.65 (m, 1H), 2.26 – 2.10 (m, 4H), 2.08 – 1.99 (m, 2H), 1.89 – 1.81 (m, 1H), 1.61 – 1.48 (m, 2H), 1.47 – 1.34 (m, 1H) ppm;

^{13}**C NMR** (126 MHz, $CDCl_3$): δ 145.4, 144.2, 138.2, 128.1, 104.8, 49.4, 37.4, 31.3, 26.5, 25.9. ppm; **LRMS (+ESI) m/z**: 225.2 $[M+Na]^+$; **HRMS (+ESI) m/z**: calc. for $C_{11}H_{14}NaN_4$ $[M+Na]^+$: 225.1111, found: 225.1107.

1-(cyclohexylmethyl)-1*H*-[1,2,3]triazolo[4,5-*c*]pyridine (12f)

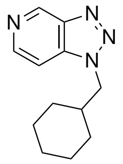

Compound **12f** was prepared according to **General Procedure D** from compound **11f** (500.1 mg). The title compound **12f** was yielded as a white solid (460.1 mg, 87%).

IR (neat, diamond cell): $\tilde{\nu}$ = 1607, 1581, 1445, 1433, 1263, 1208, 1181, 1160, 1092, 1045, 1017, 949, 927, 807 cm^{-1}; ^{1}**H NMR** (500 MHz, $CDCl_3$): δ 9.47 (s, 1H), 8.57 (d, J = 5.8 Hz, 1H), 7.45 (d, J = 5.9, 1.2 Hz, 1H), 4.48 (d, J = 7.1 Hz, 2H), 2.11 – 2.00 (m, 1H), 1.77 – 1.60 (m, 5H), 1.29 – 1.02 (m, 5H) ppm; ^{13}**C NMR** (126 MHz, $CDCl_3$): δ 144.9, 144.6, 143.1, 136.9, 104.3, 54.5, 38.6, 30.8, 26.0, 25.5 ppm; **LRMS (+ESI) m/z**: 239.2 $[M+Na]^+$; **HRMS (+ESI) m/z**: calc. for $C_{12}H_{16}NaN_4$ $[M+Na]^+$: 239.1267, found: 239.1269.

1-(adamantan-1-yl)-4,5,6,7-tetrahydro-1*H*-[1,2,3]triazolo[4,5-*c*]pyridine (13a)

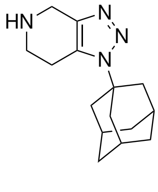

Compound **13a** was prepared according to **General Procedure E** from compound **12a** (63.8 mg). The title compound **13a** was yielded as a white powder (42.6 mg, 66%).

IR (neat, diamond cell): $\tilde{\nu}$ = 3324, 2904, 1582, 1409, 1344, 1298, 1245, 1093, 1027, 950, 853 cm^{-1}; ^{1}**H NMR** (500 MHz, $CDCl_3$): δ 4.05 (s, 2H), 3.11 (t, J = 5.7 Hz, 2H), 2.92 (t, J = 5.8 Hz, 2H), 2.52 – 2.49 (m, 1H), 2.33 – 2.29 (m, 6H), 2.26 – 2.22 (m, 3H), 1.80 – 1.75 (m, 6H) ppm; ^{13}**C NMR** (126 MHz, $CDCl_3$): δ 142.7, 129.1, 61.3, 43.3, 43.1, 42.0, 36.0, 29.6, 25.8 ppm; **LRMS (+ESI) m/z**: 259.2 $[M+H]^+$; **HRMS (+ESI) m/z**: calc. for $C_{15}H_{23}N_4$ $[M+H]^+$: 259.1917, found: 259.1912.

1-((adamantan-1-yl)methyl)-4,5,6,7-tetrahydro-1*H*-[1,2,3]triazolo[4,5-*c*]pyridine (13b)

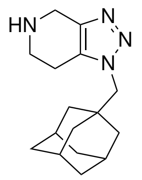

Compound **13b** was prepared according to **General Procedure E** from compound **12b** (98.1 mg). The title compound **13b** was yielded as a white powder (72.3 mg, 72%).
IR (neat, diamond cell): $\tilde{\nu}$ = 2898, 2846, 1450, 1346, 1207, 1181, 1089, 940, 831 cm^{-1}; ^{1}**H NMR** (500 MHz, $CDCl_3$): δ 4.05 (s, 2H), 3.85 (s, 2H), 3.12 (t, J = 5.7 Hz, 2H), 2.65 (t, J = 5.7 Hz, 2H), 2.02 – 1.96 (m, 3H), 1.70 – 1.57 (m, 6H), 1.57 – 1.54 (m, 6H) ppm; ^{13}**C NMR** (126 MHz, $CDCl_3$): δ 141.7, 131.4, 59.4, 42.9, 42.8, 40.6, 36.6, 35.2, 28.2, 22.9 ppm; **LRMS (+ESI) m/z**: 273.3 $[M+H]^+$; **HRMS (+ESI) m/z**: calc. for $C_{16}H_{25}N_4$ $[M+Na]^+$: 273.2073, found: 273.2071.

1-cyclopropyl-4,5,6,7-tetrahydro-1*H*-[1,2,3]triazolo[4,5-*c*]pyridine (13c)

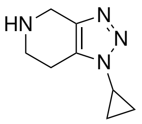

Compound **13c** was prepared according to **General Procedure E** from compound **12c** (232.4 mg). The title compound **13c** was yielded as a white powder (123.0 mg, 50%).
IR (neat, diamond cell): $\tilde{\nu}$ = 3206, 2844, 2815, 1584, 1432, 1413, 1369, 1351, 1300, 1288, 1217, 1160, 1109, 1066, 1047, 1030, 1000, 935, 890, 822 cm^{-1}; ^{1}**H NMR** (500 MHz, $CDCl_3$): δ 4.01 (d, J = 1.3 Hz, 2H), 3.51 – 3.41 (m, 1H), 3.15 (t, J = 5.8 Hz, 2H), 2.74 (t, J = 5.7, 1.4 Hz, 2H), 1.34 – 1.24 (m, 2H), 1.21 – 1.08 (m, 2H) ppm; ^{13}**C NMR** (126 MHz, $CDCl_3$): δ 142.5, 132.3, 42.7, 42.6, 28.7, 22.2, 6.0 ppm; **LRMS (+ESI) m/z**: 165.2 $[M+H]^+$; **HRMS (+ESI) m/z**: calc. for $C_8H_{12}NaN_4$ $[M+Na]^+$: 187.0954, found: 187.0955.

1-(cyclopropylmethyl)-4,5,6,7-tetrahydro-1*H*-[1,2,3]triazolo[4,5-*c*]pyridine (13d)

Compound **13d** was prepared according to **General Procedure E** from compound **12d** (150.3 mg). The title compound **13d** was yielded as a white powder (94.2 mg, 63%).

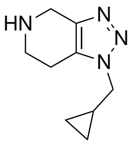

IR (neat, diamond cell): $\tilde{\nu}$ = 3219, 2922, 1428, 1354, 1334, 1266, 1169, 1115, 1082, 1028, 939, 893, 834, 813 cm^{-1}; ^{1}**H NMR** (500 MHz, $CDCl_3$): δ 4.11 (d, J = 7.0 Hz, 2H), 4.05 (s, 2H), 3.15 (t, J = 5.7 Hz, 2H), 2.71 (t, J = 5.7 Hz, 2H), 1.32 – 1.20 (m, 1H), 0.69 – 0.59 (m, 2H), 0.44 – 0.38 (m, 2H) ppm; ^{13}**C NMR** (126 MHz, $CDCl_3$): δ 142.5, 130.1, 52.6, 42.8, 42.7, 22.2, 11.1, 4.3 ppm; **LRMS (+ESI) m/z**: 179.2 [M+H]$^+$; **HRMS (+ESI) m/z**: calc. for $C_9H_{15}N_4$ [M+Na]$^+$: 179.1291, found: 179.1288.

1-cyclohexyl-4,5,6,7-tetrahydro-1*H*-[1,2,3]triazolo[4,5-*c*]pyridine (13e)

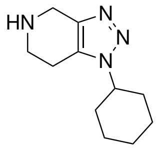

Compound **13e** was prepared according to **General Procedure E** from compound **12e** (90.3 mg). The title compound **13e** was yielded as a white powder (65.8 mg, 71%).

IR (neat, diamond cell): $\tilde{\nu}$ = 3217, 2926, 2855, 1584, 1451, 1332, 1289, 1206, 1149, 1096, 999, 941, 892, 830 cm^{-1}; ^{1}**H NMR** (500 MHz, $CDCl_3$): δ 4.21 – 4.13 (m, 1H), 4.03 (s, 2H), 3.14 (t, J = 5.7 Hz, 2H), 2.69 (t, J = 5.7 Hz, 2H), 2.09 – 1.90 (m, 7H), 1.78 – 1.72 (m, 1H), 1.47 – 1.24 (m, 3H) ppm; ^{13}**C NMR** (126 MHz, $CDCl_3$): δ 142.2, 129.4, 58.7, 42.9, 42.8, 32.7, 25.5, 25.1, 22.5 ppm; **LRMS (+ESI) m/z**: 207.2 [M+H]$^+$; **HRMS (+ESI) m/z**: calc. for $C_{11}H_{19}N_4$ [M+Na]$^+$: 207.1604, found: 207.1604.

1-(cyclohexylmethyl)-4,5,6,7-tetrahydro-1*H*-[1,2,3]triazolo[4,5-*c*]pyridine (13f)

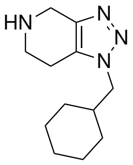

Compound **13f** was prepared according to **General Procedure E** from compound **12f** (44.0 mg). The title compound **13f** was yielded as a white powder (22.9 mg, 51%).

IR (neat, diamond cell): $\tilde{\nu}$ = 3273, 2920, 2851, 1583, 1447, 1350, 1302, 1238, 1194, 1093, 1065, 1047, 941, 875, 833, 814 cm^{-1}; ^{1}**H NMR** (500 MHz, $CDCl_3$): δ 4.06 – 4.01 (m, 4H), 3.14 (t, J = 5.7 Hz, 2H), 2.65 (t, J = 5.7, 2H), 1.97 – 1.86 (m, 3H), 1.76 – 1.59 (m, 4H), 1.29 – 1.12 (m, 3H), 1.05 – 0.93 (m, 2H) ppm; ^{13}**C NMR** (126 MHz, $CDCl_3$): δ 142.0, 130.7, 53.9, 42.8, 42.7, 38.6, 30.7, 26.1, 25.6, 22.2 ppm; **LRMS (+ESI) m/z**: 221.3 $[M+H]^+$; **HRMS (+ESI) m/z**: calc. for $C_{12}H_{21}N_4$ $[M+H]^+$: 221.1761, found: 221.1759.

(1-(adamantan-1-yl)-1,4,6,7-tetrahydro-5*H*-[1,2,3]triazolo[4,5-*c*]pyridin-5-yl)(2-chloro-3-(trifluoromethyl)phenyl)methanone (2a)

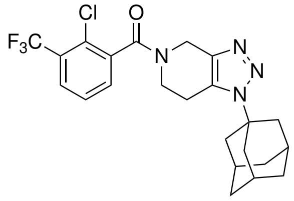

Compound **2a** was prepared according to **General Procedure F** from 2-chloro-3-(trifluoromethyl)benzoic acid (57.6 mg, 0.26 mmol) and compound **13a** (86.2 mg). The crude product was purified by flash chromatography (0-50% EtOAc/Hex) to yield the title compound **2a** as a white powder (41.7 mg, 35%).

IR (neat, diamond cell): $\tilde{\nu}$ = 2909, 1647, 1422, 1319, 1205, 1177, 1134, 1088, 1027, 817 cm^{-1}; ^{1}**H NMR** (500 MHz, $CDCl_3$): δ 7.81 – 7.74 (m, 1H), 7.54 – 7.41 (m, 2H), 5.09 – 4.93 (m, 1H), 4.53 – 4.36 (m, 1H), 4.35 – 4.27 (m, 0.5H), 3.90 (m, 0.5H), 3.57 – 3.44 (m, 1H), 3.18 – 3.07 (m, 1H), 3.06 – 2.99 (m, 0.5H), 2.92 – 2.83 (m, 0.5H), 2.35 – 2.23 (m, 9H), 1.85 – 1.72 (m, 6H) ppm; ^{13}**C NMR** (126 MHz, $CDCl_3$): δ 166.7, 166.3, 140.4, 140.1, 138.5, 138.0, 131.0, 131.0, 129.7, 129.6, 129.4, 129.4, 129.2, 128.7, 128.4, 128.3,

128.3, 128.2, 128.2, 127.9, 127.5, 127.5, 123.5, 121.4, 121.4, 61.9, 61.7, 44.4, 44.0, 42.0, 42.0, 40.1, 39.4, 35.9, 29.5, 29.5, 24.9, 23.9 ppm; ^{19}**F NMR** (471 MHz, $CDCl_3$): δ −62.5, −62.5 ppm; **LRMS (+ESI) m/z**: 487.1/489.1 $[M+Na]^+$; **HRMS (+ESI) m/z**: calc. for $C_{23}H_{24}ClF_3N_4NaO$ $[M+Na]^+$: 487.1483/489.1453, found: 487.1477/489.1447. **HPLC:** R_T = 26.7 min, 98.8% (254 nm).

(1-(adamantan-1-yl)methyl)-1,4,6,7-tetrahydro-5*H*-[1,2,3]triazolo[4,5-*c*]pyridin-5-yl)(2-chloro-3-(trifluoromethyl)phenyl)methanone (2b)

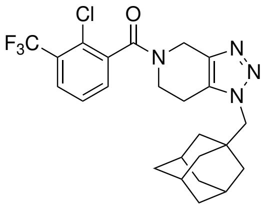

Compound **2b** was prepared according to **General Procedure F** from 2-chloro-3-(trifluoromethyl)benzoic acid (79.4 mg, 0.32 mmol) and compound **13b** (66.1 mg). The crude product was purified by flash chromatography (0-100% EtOAc/Hex) to yield the title compound **2b** as a white powder (79.5 mg, 89%).

IR (neat, diamond cell): $\tilde{\nu}$ = 2904, 1650, 1422, 1319, 1208, 1137, 1088 cm^{-1}; ^{1}**H NMR** (500 MHz, $CDCl_3$): δ 7.81 – 7.74 (m, 1H), 7.54 – 7.42 (m, 2H), 5.09 – 4.98 (m, 1H), 4.54 – 4.37 (m, 1H), 4.27 – 4.19 (m, 0.5H), 4.07 – 3.99 (m, 0.5H), 3.94 – 3.84 (m, 2H), 3.60 – 3.47 (m, 1H), 2.95 – 2.84 (m, 1H), 2.83 – 2.75 (m, 0.5H), 2.66 – 2.57 (m, 0.5H), 2.04 – 1.98 (m, 3H), 1.75 – 1.68 (m, 3H), 1.63 – 1.57 (m, 3H), 1.56 – 1.51 (m, 6H) ppm; ^{13}**C NMR** (126 MHz, $CDCl_3$): δ 166.9, 166.6, 139.3, 139.0, 138.5, 138.0, 131.5, 130.9, 130.9, 130.2, 129.9, 129.7, 129.4, 129.2, 128.8, 128.8, 128.7, 128.7, 128.5, 128.4, 128.4, 128.3, 128.3, 128.3, 128.2, 128.2, 127.6, 127.5, 125.7, 123.6, 123.5, 121.4, 121.4, 119.2, 77.3, 77.2, 77.0, 76.8, 59.8, 59.8, 44.4, 43.9, 40.6, 40.5, 40.1, 39.4, 36.5, 36.5, 35.2, 28.1, 22.2, 21.2 ppm; ^{19}**F NMR** (471 MHz, $CDCl_3$): δ −62.5, −62.5 ppm; **LRMS (+ESI) m/z**: 501.2/503.1 $[M+Na]^+$; **HRMS (+ESI) m/z**: calc. for $C_{24}H_{27}ClF_3N_4O$ $[M+H]^+$: 479.1820/481.1791, found: 479.1815/481.1785. **HPLC:** R_T = 27.6 min, 95.9% (254 nm).

(2-chloro-3-(trifluoromethyl)phenyl)(1-cyclopropyl-1,4,6,7-tetrahydro-5*H*-[1,2,3]triazolo[4,5-*c*]pyridin-5-yl)methanone (2d)

Compound **2d** was prepared according to **General Procedure F** from 2-chloro-3-(tri-

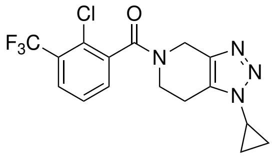

fluoromethyl)benzoic acid (53.0 mg, 0.24 mmol) and compound **13c** (50.4 mg). The crude product was purified by flash chromatography (0-5% CH_2Cl_2/MeOH) to yield the title compound **2d** as a white powder (66.5 mg, 76%).

IR (neat, diamond cell): $\tilde{\nu}$ = 1640, 1422, 1318, 1131, 925, 815 cm^{-1}; ^{1}**H NMR** (500 MHz, $CDCl_3$): δ 7.82 – 7.70 (m, 1H), 7.54 – 7.36 (m, 2H), 5.08 – 4.91 (m, 1H), 4.50 – 4.35 (m, 1H), 4.35 – 4.29 (m, 0.5H), 4.02 – 3.94 (m, 0.5H), 3.61 – 3.43 (m, 2H), 3.03 – 2.94 (m, 1H), 2.94 – 2.83 (m, 0.5H), 2.77 – 2.67 (m, 0.5H), 1.36 – 1.24 (m, 2H), 1.24 – 1.11 (m, 2H) ppm; ^{13}**C NMR** (126 MHz, $CDCl_3$): δ 166.8, 166.6, 140.0, 139.7, 138.5, 138.0, 132.4, 131.1, 131.0, 130.9, 129.7, 129.4, 128.8, 128.7, 128.5, 128.4, 128.4, 128.4, 128.3, 128.3, 127.5, 127.5, 123.6, 123.5, 121.4, 121.4, 44.2, 43.7, 39.9, 39.2, 29.1, 29.0, 21.5, 20.6, 6.2, 6.2 ppm; ^{19}**F NMR** (471 MHz, $CDCl_3$): δ −62.5, −62.5 ppm; **LRMS (+ESI) m/z**: 393.1/395.1 [M+Na]$^+$; **HRMS (+ESI) m/z**: calc. for $C_{16}H_{14}ClF_3N_4NaO$ [M+Na]$^+$: 393.0700/395.0671, found: 393.0697/395.0669. **HPLC:** R_T = 20.9 min, 98.2% (254 nm).

(2-chloro-3-(trifluoromethyl)phenyl)(1-(cyclopropylmethyl)-1,4,6,7-tetrahydro-5*H*-[1,2,3]triazolo[4,5-*c*]pyridin-5-yl)methanone (2e)

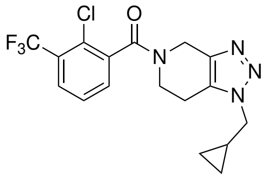

Compound **2e** was prepared according to **General Procedure F** from 2-chloro-3-(trifluoromethyl)benzoic acid (83.3 mg, 0.37 mmol) and compound **13d** (86.0 mg). The crude product was purified by flash chromatography (0-100% EtOAc/Hex) to yield the title compound **2e** as a white powder (54.2 mg, 38%).

IR (neat, diamond cell): $\tilde{\nu}$ = 1641, 1422, 1319, 1207, 1130, 1087, 841 cm^{-1}; ^{1}**H NMR** (500 MHz, $CDCl_3$): δ 7.82 – 7.74 (m, 1H), 7.54 – 7.43 (m, 2H), 5.10 – 4.96 (m, 1H), 4.54 – 4.38 (m, 1H), 4.37 – 4.29 (m, 0.5H), 4.21 – 4.08 (m, 2H), 4.03 – 3.94 (m, 0.5H), 3.64 – 3.49 (m, 1H), 2.96 – 2.90 (m, 1H), 2.90 – 2.80 (m, 0.5H), 2.73 – 2.64 (m, 0.5H), 1.33

– 1.19 (m, 1H), 0.73 – 0.61 (m, 2H), 0.46 – 0.38 (m, 2H) ppm; ^{13}**C NMR** (126 MHz, $CDCl_3$): δ 166.9, 166.6, 140.1, 139.8, 138.5, 138.0, 131.0, 130.9, 130.2, 129.7, 129.4, 129.2, 128.9, 128.8, 128.7, 128.5, 128.4, 128.4, 128.4, 128.3, 128.3, 127.6, 127.5, 123.5, 121.4, 53.1, 53.0, 44.3, 43.8, 40.0, 39.3, 21.6, 20.7, 11.0, 11.0, 4.4, 4.4, 4.3 ppm; ^{19}**F NMR** (471 MHz, $CDCl_3$): δ −62.5, −62.5 ppm; **LRMS (+ESI) m/z**: 407.0/409.0 $[M+Na]^+$; **HRMS (+ESI) m/z**: calc. for $C_{17}H_{16}ClF_3N_4NaO$ $[M+Na]^+$: 407.0857/409.0874 found: 407.0851/409.0822. **HPLC:** R_T = 22.5 min, 96.3% (254 nm).

(2-chloro-3-(trifluoromethyl)phenyl)(1-cyclohexyl-1,4,6,7-tetrahydro-5*H*-[1,2,3]triazolo[4,5-*c*]pyridin-5-yl) (2f)

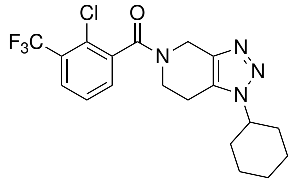

Compound **2f** was prepared according to **General Procedure F** from 2-chloro-3-(trifluoromethyl)benzoic acid (79.4 mg, 0.32 mmol) and compound **13e** (94.8 mg). The crude product was purified by flash chromatography (0-50% EtOAc/Hex) to yield the title compound **2f** as a white powder (68.6 mg, 47%).

IR (neat, diamond cell): $\tilde{\nu}$ = 2935, 2859, 1645, 1422, 1318, 1207, 1133, 1088, 817 cm^{-1}; ^{1}**H NMR** (500 MHz, $CDCl_3$): δ 7.81 – 7.73 (m, 1H), 7.54 – 7.41 (m, 2H), 5.08 – 4.95 (m, 1H), 4.52 – 4.36 (m, 1H), 4.36 – 4.29 (m, 0.5H), 4.24 – 4.11 (m, 1H), 4.00 – 3.91 (m, 0.5H), 3.62 – 3.47 (m, 1H), 2.94 – 2.88 (m, 1H), 2.87 – 2.78 (m, 0.5H), 2.72 – 2.60 (m, 0.5H), 2.10 – 1.92 (m, 6H), 1.81 – 1.73 (m, 1H), 1.51 – 1.24 (m, 3H) ppm; ^{13}**C NMR** (126 MHz, $CDCl_3$): δ 166.8, 166.5, 139.8, 139.5, 138.5, 138.0, 131.0, 130.9, 129.9, 129.7, 129.5, 129.4, 129.2, 128.8, 128.7, 128.4, 128.4, 128.4, 128.3, 128.3, 128.2, 128.2, 127.5, 127.5, 125.7, 123.6, 123.5, 121.4, 121.4, 119.2, 59.1, 59.1, 44.3, 43.8, 40.0, 39.2, 32.8, 32.7, 32.7, 25.4, 25.1, 21.8, 20.8 ppm; ^{19}**F NMR** (471 MHz, $CDCl_3$): δ −62.5, −62.5 ppm; **LRMS (+ESI) m/z**: 435.1/437.1 $[M+Na]^+$; **HRMS (+ESI) m/z**: calc. for $C_{19}H_{20}ClF_3N_4NaO$ $[M+Na]^+$: 435.1170/437.1140, found: 435.1168/437.1138. **HPLC:** R_T = 24.0 min, 95.2% (254 nm).

(2-chloro-3-(trifluoromethyl)phenyl)(1-(cyclopropylmethyl)-1,4,6,7-tetrahydro-5*H*-[1,2,3]triazolo[4,5-*c*]pyridin-5-yl)methanone (2g)

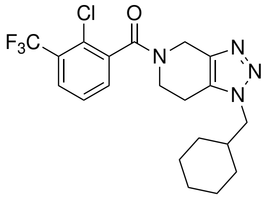

Compound **2g** was prepared according to **General Procedure F** from 2-chloro-3-(trifluoromethyl)benzoic acid (36.7 mg, 0.16 mmol) and compound **13f** (46.8 mg). The

crude product was purified by flash chromatography (0-100% EtOAc/Hex) to yield the title compound **2g** as a white powder (55.8 mg, 80%).

IR (neat, diamond cell): $\tilde{\nu}$ = 2927, 1640, 1422, 1319, 1208, 1177, 1135, 802 cm^{-1}; ^{1}**H NMR** (500 MHz, $CDCl_3$): δ 7.82 – 7.74 (m, 1H), 7.54 – 7.42 (m, 2H), 5.08 – 4.96 (m, 1H), 4.52 – 4.36 (m, 1H), 4.26 (dt, J = 13.2, 5.6 Hz, 0.5H), 4.13 – 3.98 (m, 2.5H), 3.62 – 3.47 (m, 1H), 2.93 – 2.85 (m, 1H), 2.82 – 2.73 (m, 0.5H), 2.66 – 2.57 (m, 0.5H), 1.98 – 1.84 (m, 1H), 1.79 – 1.65 (m, 3H), 1.64 – 1.58 (m, 2H), 1.30 – 1.11 (m, 3H), 1.07 – 0.93 (m, 2H) ppm; ^{13}**C NMR** (126 MHz, $CDCl_3$): δ 166.9, 166.6, 139.6, 139.4, 138.5, 138.0, 130.9, 130.8, 129.7, 129.5, 129.4, 129.2, 128.8, 128.7, 128.5, 128.4, 128.4, 128.4, 128.3, 128.2, 127.6, 127.5, 123.5, 121.4, 121.3, 54.3, 54.2, 44.4, 43.8, 40.0, 39.3, 38.6, 38.6, 30.6, 30.6, 26.0, 25.5, 21.6, 20.6 ppm; ^{19}**F NMR** (471 MHz, $CDCl_3$): δ −62.5 −62.5 ppm; **LRMS (+ESI) m/z**: 449.1/451.1 $[M+Na]^+$; **HRMS (+ESI) m/z**: calc. for $C_{20}H_{22}ClF_3N_4NaO$ $[M+Na]^+$: 449.1326/451.1296, found: 449.1325/451.1295. **HPLC:** R_T = 25.8 min, 96.3% (254 nm).

azidobenzene (14)

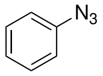

A solution of phenylhydrazine (211 mg, 1.95 mmol) was dissolved in dry MeCN (3 mL) and cooled to 0 °C. The solution was treated with *tert*-butyl nitrite (2.40 mL, 20.1 mmol) and CH_3COOH (2 drops). The solution was stirred for 5 minutes before being diluted in EtOAc (10 mL). The organic phase was washed with Na_2CO_3 (3 × 15 mL) and brine (10 mL). The organic phase was dried over $MgSO_4$, concentrated *in vacuo* to a minimal volume of EtOAc (1 mL) and used without further purification.

***tert*-butyl-1-phenyl-1,3a,4,6,7,7a-hexahydro-5*H*-[1,2,3]triazolo[4,5-*c*]pyridine-5-carboxylate (15)**

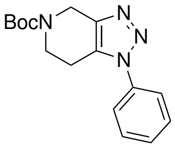

To a solution of *tert*-butyl 4-oxopiperidine-1-carboxylate (388.5 mg, 1 eq.) in MePh (15 mL) was added pyrrolidine (0.16 mL, 1 eq.). The solution was stirred at room temperature for 3 hours. The reaction mixture was then treated with a solution of **14** (232.4 mg, 1.3 eq.) in EtOAc (1 mL) and heated to reflux for 16 hours. The reaction mixture was then cooled over ice and diluted with CH_2Cl_2 (12 mL), followed by the addition of $NaHCO_3$ (163.4 mg, 1 eq.) and *m*-CPBA (504.8 mg, 1.5 eq.). The reaction mixture was stirred over ice for 30 minutes then quenched in NaOH (1.0 M, 10 mL). The reaction mixture was extracted with CH_2Cl_2 (3 × 15 mL). The organics were combined, dried over $MgSO_4$ and concentrated *in vacuo*. The crude product was purified by flash column chromatography (0-100% EtOAc/Hex) to yield **15** as a white solid (418.6 mg, 71%).

^{1}H NMR (500 MHz, $CDCl_3$): δ 7.58 – 7.52 (m, 4H), 7.51 – 7.45 (m, 1H), 4.73 (s, 2H), 3.76 (s, 2H), 2.87 (t, J = 5.6 Hz, 2H), 1.50 (s, 9H) ppm; **^{13}C NMR** (126 MHz, $CDCl_3$): δ 154.9, 141.7, 136.4, 130.9, 129.7, 129.0, 122.9, 80.7, 41.8, 40.4, 28.4, 22.4 ppm; **LRMS (+ESI) m/z**: 323.2 $[M+Na]^+$; Characterisation is consistent with literature.[2]

1-phenyl-3a,4,5,6,7,7a-hexahydro-1*H*-[1,2,3]triazolo[4,5-*c*]pyridine (13g)

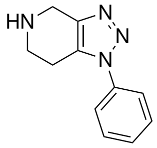

A solution of **15** (200.0 mg) in CH_2Cl_2 (5 mL) was treated with HCl·$_{1,4}$−Dioxane (4.0 M, 2.0 mL) and stirred at room temperature for 16 hours. The reaction mixture was quenched in NaOH (1.0 M, 50 mL) and extracted with CH_2Cl_2 (3 × 80 mL). The organics were combined, dried over $MgSO_4$ and concentrated *in vacuo* to yield a pale yellow powder (123.1 mg, 92%).

^{1}**H NMR** (500 MHz, DMSO−d_6): δ 7.68 – 7.64 (m, 2H), 7.63 – 7.59 (m, 2H), 7.55 – 7.51 (m, 1H), 3.87 (s, 2H), 2.95 (t, J = 5.6 Hz, 2H), 2.76 (tt, J = 5.7, 1.4 Hz, 2H) ppm; ^{13}**C NMR** (126 MHz, DMSO−d_6): δ 143.4, 136.8, 132.0, 130.2, 129.2, 123.2, 42.8, 42.2, 23.3 ppm; **LRMS (+ESI) m/z**: 201.2 $[M+H]^+$; Characterisation is consistent with literature.[2]

(2-chloro-3-(trifluoromethyl)phenyl)(1-phenyl-1,3a,4,6,7,7a-hexahydro-5*H*-[1,2,3]triazolo[4,5-*c*]pyridin-5-yl)methanone (2c)

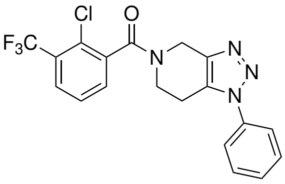

Compound **2c** was prepared according to **General Procedure F** from 2-chloro-3-(trifluoromethyl)benzoic acid (71 mg, 0.32 mmol) and compound **13g** (83 mg). The crude product was purified by flash chromatography (0-100% EtOAc/Hex) to yield the title compound **2c** as a white powder (107 mg, 83%).

^{1}**H NMR** (500 MHz, $CDCl_3$): δ 7.82 – 7.77 (m, 1H), 7.61 – 7.44 (m, 7H), 5.19 – 5.05 (m, 1H), 4.63 – 4.46 (m, 1H), 4.36 – 4.28 (m, 0.5H), 4.04 – 3.96 (m, 0.5H), 3.64 – 3.49 (m, 1H), 3.08 – 3.01 (m, 1H), 2.99 – 2.90 (m, 0.5H), 2.85 – 2.76 (m, 0.5H) ppm; ^{13}**C NMR** (126 MHz, $CDCl_3$): δ 166.9, 166.6, 140.7, 140.4, 138.4, 138.0, 136.2, 131.0, 131.0, 130.8, 129.8, 129.8, 129.5, 129.5, 129.3, 129.2, 128.8, 128.7, 128.6, 128.5, 128.5, 128.4, 128.4, 128.3, 127.6, 122.8, 122.8, 121.4, 44.2, 43.9, 40.0, 39.4, 23.0, 22.1 ppm; ^{19}**F NMR**

(471 MHz, $CDCl_3$): δ −62.5, −62.5 ppm; **LRMS (+ESI) m/z**: 429.0/431.1 $[M+Na]^+$; **HPLC:** R_T = 23.4 min, 99.2% (254 nm). Characterisation is consistent with literature.[2]

2.5 HPLC Traces of biologically tested compounds

Project Name:	P2X7_Hybrids	Acq. Method Set:	JD_0to100ov30 HPLC4
Sample Name:	NLD11	Sample Set Name:	NLD11_2301107
Injection Volume:	5.00 ul		Wavelength W2489 ChA 254nm
Run Time:	52.0 Minutes		

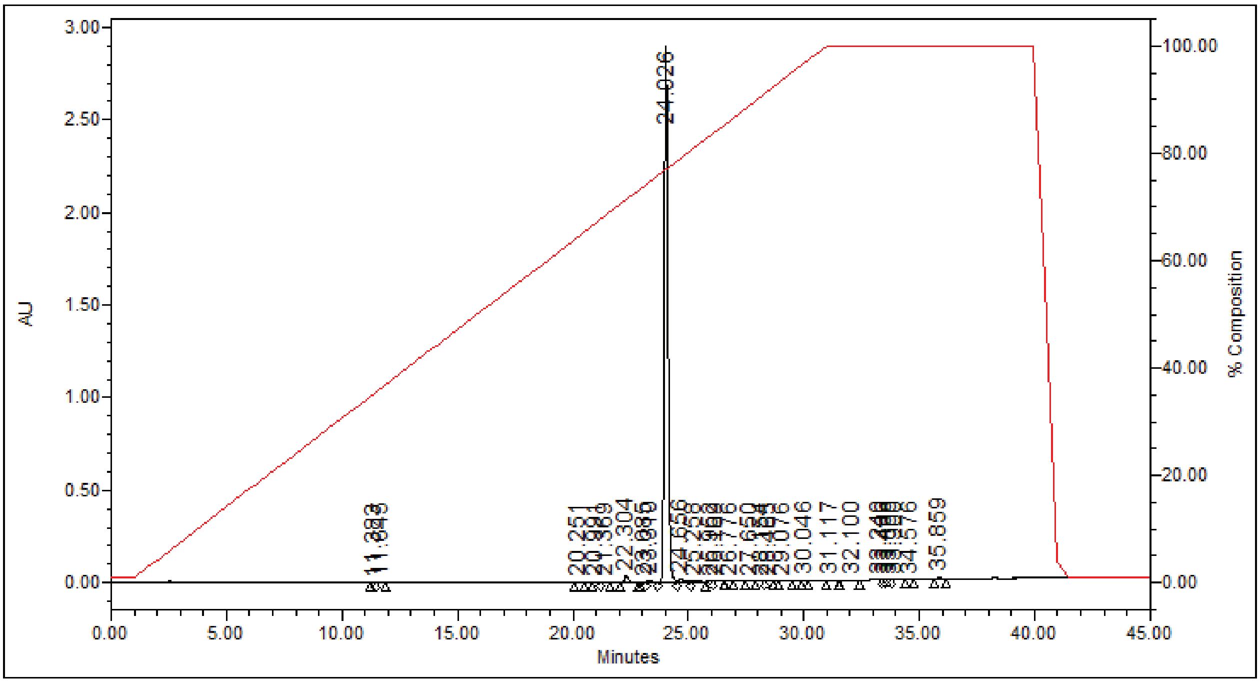

Peak information

	RT	Area	% Area		RT	Area	% Area		RT	Area	% Area
1	11.383	4136	0.01	12	25.952	27676	0.09	23	33.483	1449	0.00
2	11.645	32007	0.10	13	26.169	34617	0.11	24	33.686	4650	0.01
3	20.251	15224	0.05	14	26.776	8017	0.02	25	33.900	4685	0.01
4	20.991	7000	0.02	15	27.650	3873	0.01	26	34.576	9260	0.03
5	21.369	22622	0.07	16	28.154	8716	0.03	27	35.859	80841	0.25
6	22.304	356516	1.11	17	28.465	11314	0.04				
7	23.085	1984	0.01	18	29.076	6659	0.02				
8	23.310	78290	0.24	19	30.046	4903	0.02				
9	24.026	31116043	96.92	20	31.117	1856	0.01				
10	24.656	222700	0.69	21	32.100	4943	0.02				
11	25.258	26188	0.08	22	33.240	8149	0.03				

Figure 3: HPLC trace of compound **1a**

Project Name:	P2X7_Hybrids	Acq. Method Set:	_Generic 0to100ov30 HPLC10
Sample Name:	251023_NLD10	Sample Set Name:	NLD10_231025
Injection Volume:	10.00 ul	**Wavelength W2489 ChA 254nm**	
Run Time:	50.0 Minutes		

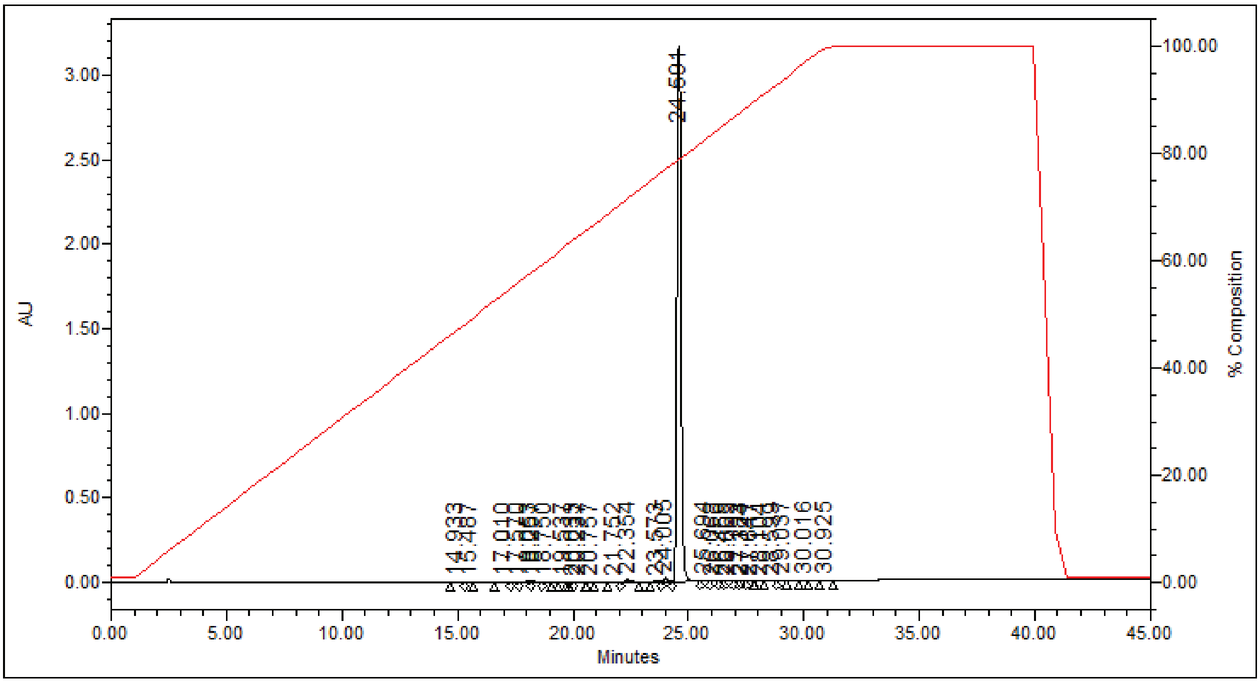

Peak information

	RT	Area	% Area		RT	Area	% Area		RT	Area	% Area
1	14.933	24660	0.06	12	21.752	6786	0.02	23	27.647	12383	0.03
2	15.487	25130	0.06	13	22.354	202374	0.47	24	28.104	6721	0.02
3	17.010	37405	0.09	14	23.573	45606	0.11	25	28.599	12801	0.03
4	17.570	13567	0.03	15	24.005	218206	0.51	26	29.037	12105	0.03
5	18.053	103566	0.24	16	24.591	41851955	97.65	27	30.016	5250	0.01
6	18.257	73353	0.17	17	25.694	73676	0.17	28	30.925	9251	0.02
7	18.750	8217	0.02	18	26.068	43243	0.10				
8	19.537	4080	0.01	19	26.459	6625	0.02				
9	20.033	2100	0.00	20	26.665	12117	0.03				
10	20.237	21757	0.05	21	27.183	2992	0.01				
11	20.757	8009	0.02	22	27.324	13814	0.03				

Figure 4: HPLC trace of compound **1b**

Project Name:	P2X7_Hybrids	Acq. Method Set:	JD_0to100ov30 HPLC4
Sample Name:	NLD28b_s1	Sample Set Name:	240627_WT5104
Injection Volume:	10.00 ul		
Run Time:	52.0 Minutes		

Wavelength W2489 ChA 254nm

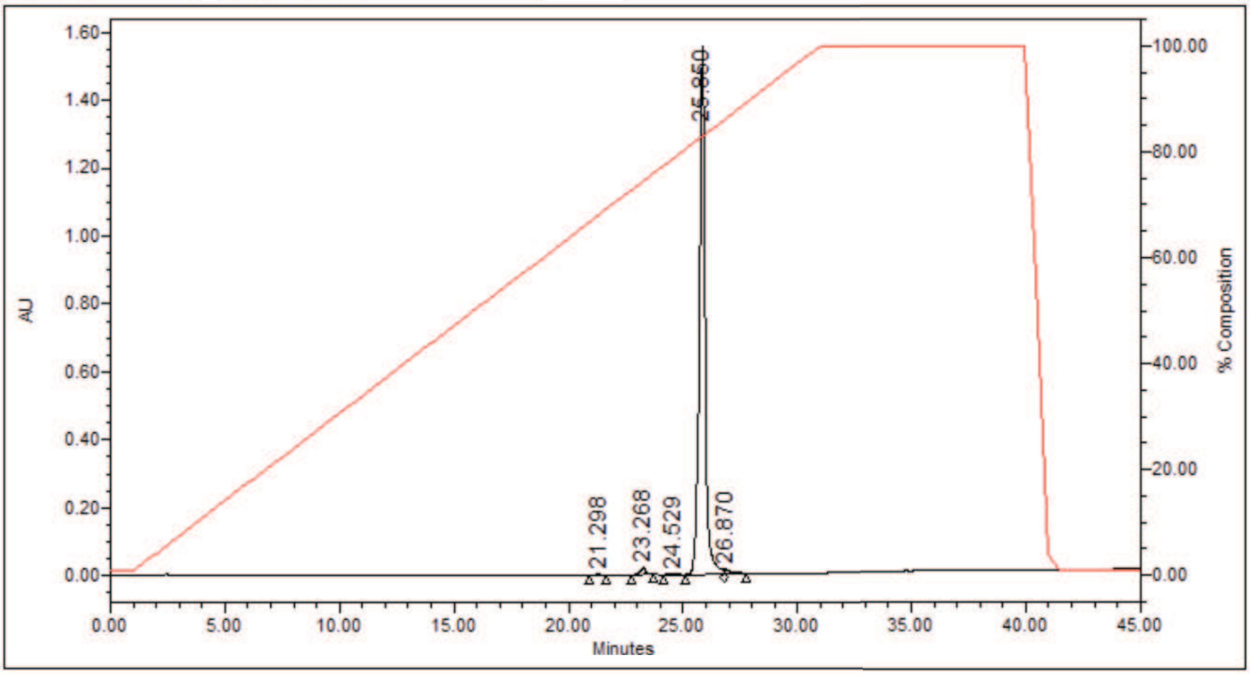

Peak information

	RT	Area	% Area
1	21.298	43972	0.16
2	23.268	330717	1.23
3	24.529	42171	0.16
4	25.850	26134972	97.54
5	26.870	241420	0.90

Figure 5: HPLC trace of compound **1c**

Project Name:	P2X7_Hybrids	Acq. Method Set:	JD_0to100ov30 HPLC4
Sample Name:	NLD27	Sample Set Name:	230224_NLD23_24_25_27
Injection Volume:	5.00 ul		
Run Time:	52.0 Minutes		

Wavelength W2489 ChA 254nm

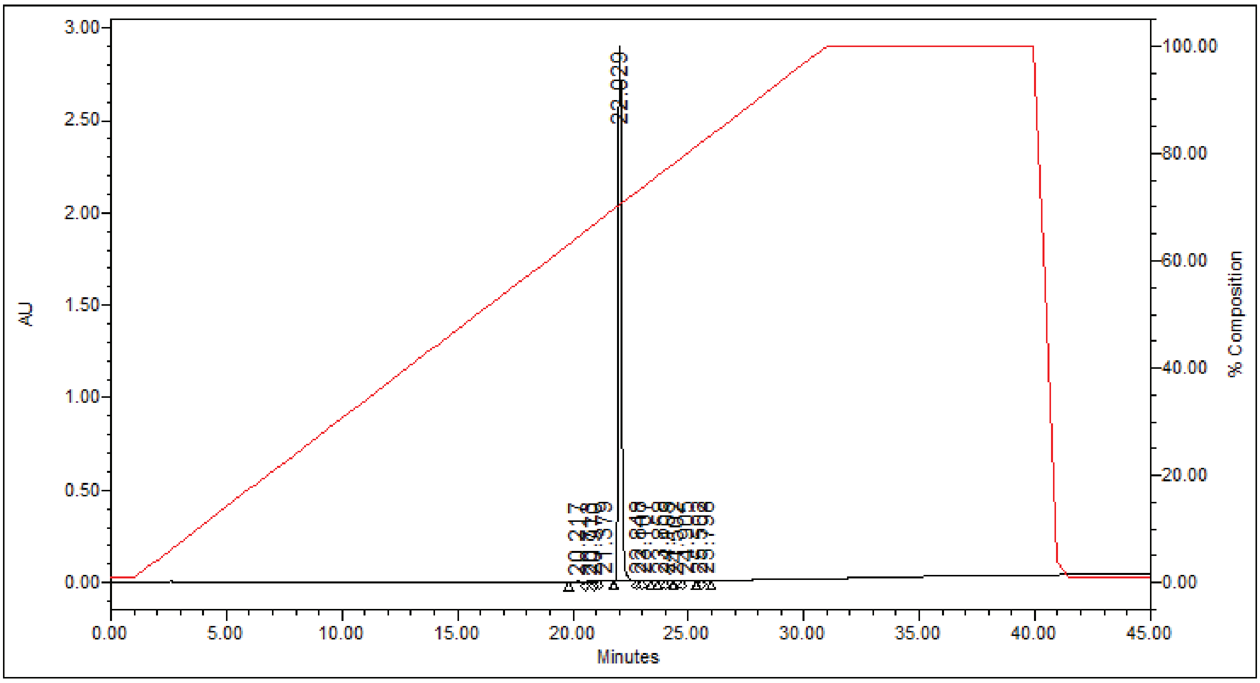

Peak information

	RT	Area	% Area
1	20.217	16586	0.06
2	20.713	36009	0.12
3	20.970	7869	0.03
4	21.379	70547	0.24
5	22.029	29132643	99.15
6	22.848	23991	0.08
7	23.107	23124	0.08
8	23.858	34495	0.12
9	24.105	11414	0.04
10	24.502	7499	0.03
11	24.905	7553	0.03

	RT	Area	% Area
12	25.502	1771	0.01
13	25.798	8381	0.03

Figure 6: HPLC trace of compound **1d**

Project Name:	P2X7_Hybrids	Acq. Method Set:	JD_0to100ov30 HPLC4
Sample Name:	NLC30	Sample Set Name:	240605_DTCompounds
Injection Volume:	10.00 ul		
Run Time:	53.0 Minutes		

Wavelength W2489 ChA 254nm

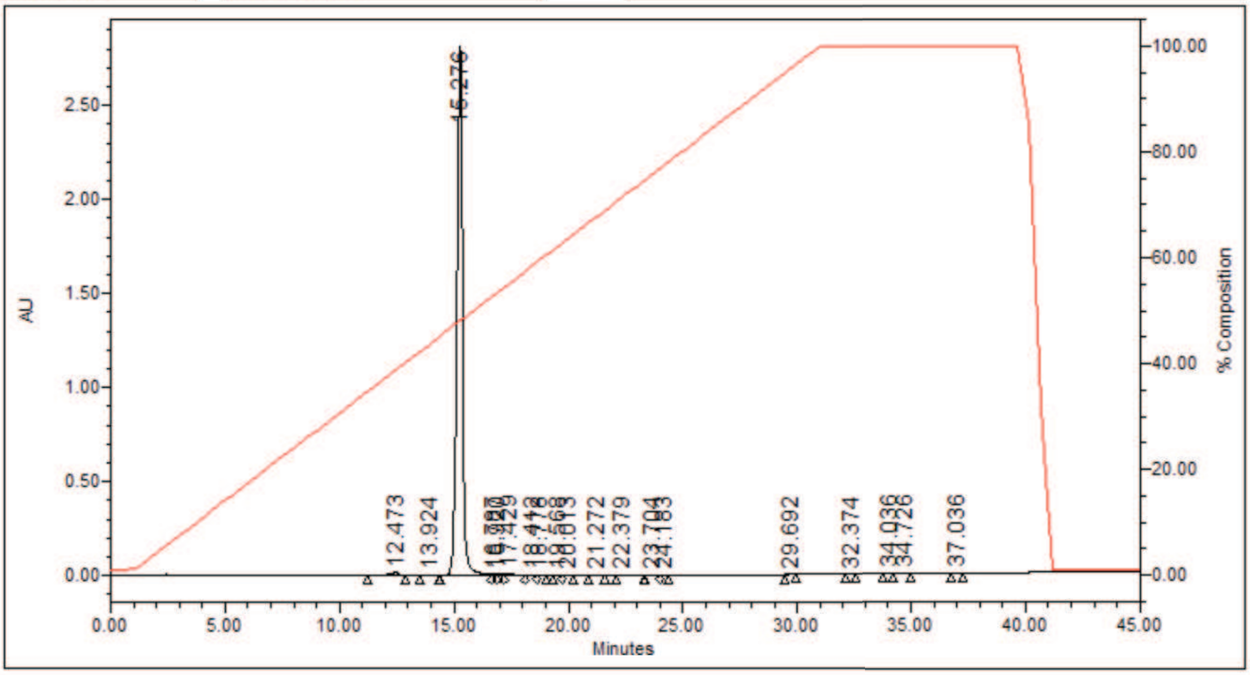

Peak information

	RT	Area	% Area
1	12.473	477170	0.95
2	13.924	44448	0.09
3	15.276	49119424	97.52
4	16.787	107302	0.21
5	16.920	117849	0.23
6	17.429	259414	0.52
7	18.412	43503	0.09
8	18.778	21628	0.04
9	19.568	10788	0.02
10	20.013	8046	0.02
11	21.272	54101	0.11

	RT	Area	% Area
12	22.379	16926	0.03
13	23.704	12763	0.03
14	24.183	8564	0.02
15	29.692	11734	0.02
16	32.374	7646	0.02
17	34.036	6288	0.01
18	34.726	30283	0.06
19	37.036	12616	0.03

Figure 7: HPLC trace of compound **1e**

Project Name:	P2X7_Hybrids	Acq. Method Set:	JD_0to100ov30 HPLC4
Sample Name:	NLD34b	Sample Set Name:	240816_NLD34b
Injection Volume:	10.00 ul		Wavelength W2489 ChA 254nm
Run Time:	53.0 Minutes		

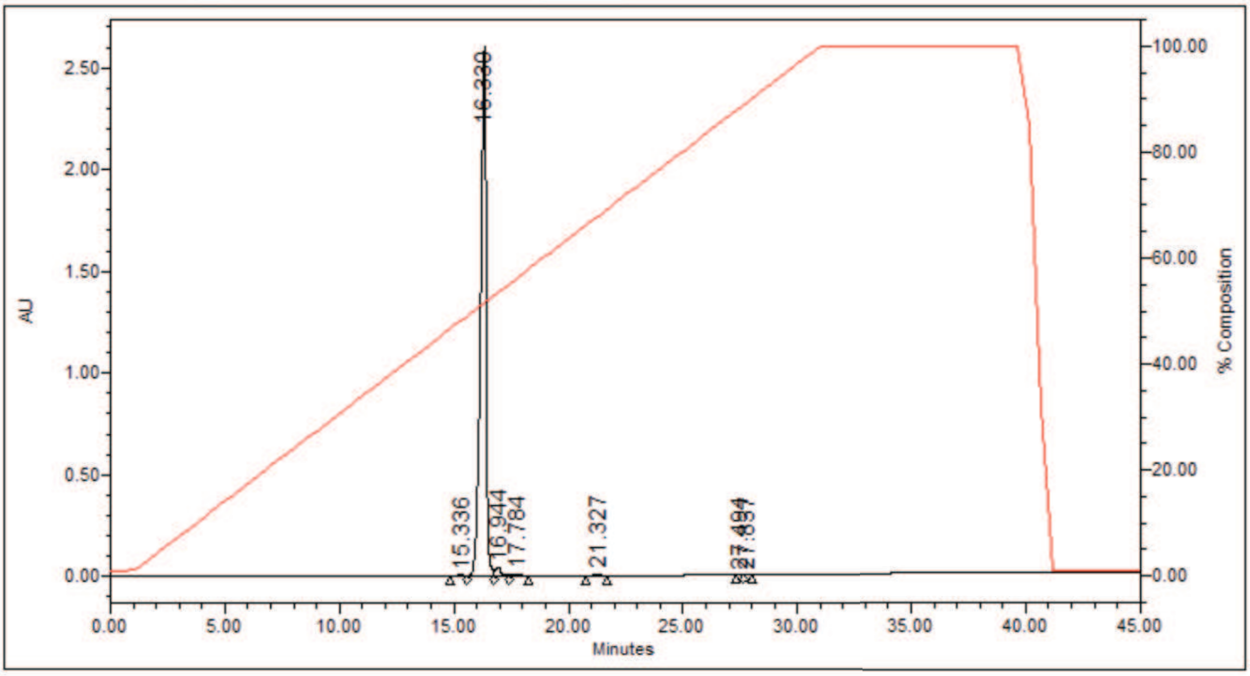

Peak information

	RT	Area	% Area
1	15.336	117939	0.26
2	16.330	45002051	97.53
3	16.944	704974	1.53
4	17.784	185231	0.40
5	21.327	117020	0.25
6	27.494	5653	0.01
7	27.837	7697	0.02

Figure 8: HPLC trace of compound **1f**

Project Name:	P2X7_Hybrids	Acq. Method Set:	JD_0to100ov30 HPLC4
Sample Name:	NLD31b	Sample Set Name:	240627_WT5104
Injection Volume:	5.00 ul		
Run Time:	52.0 Minutes		

Wavelength W2489 ChA 254nm

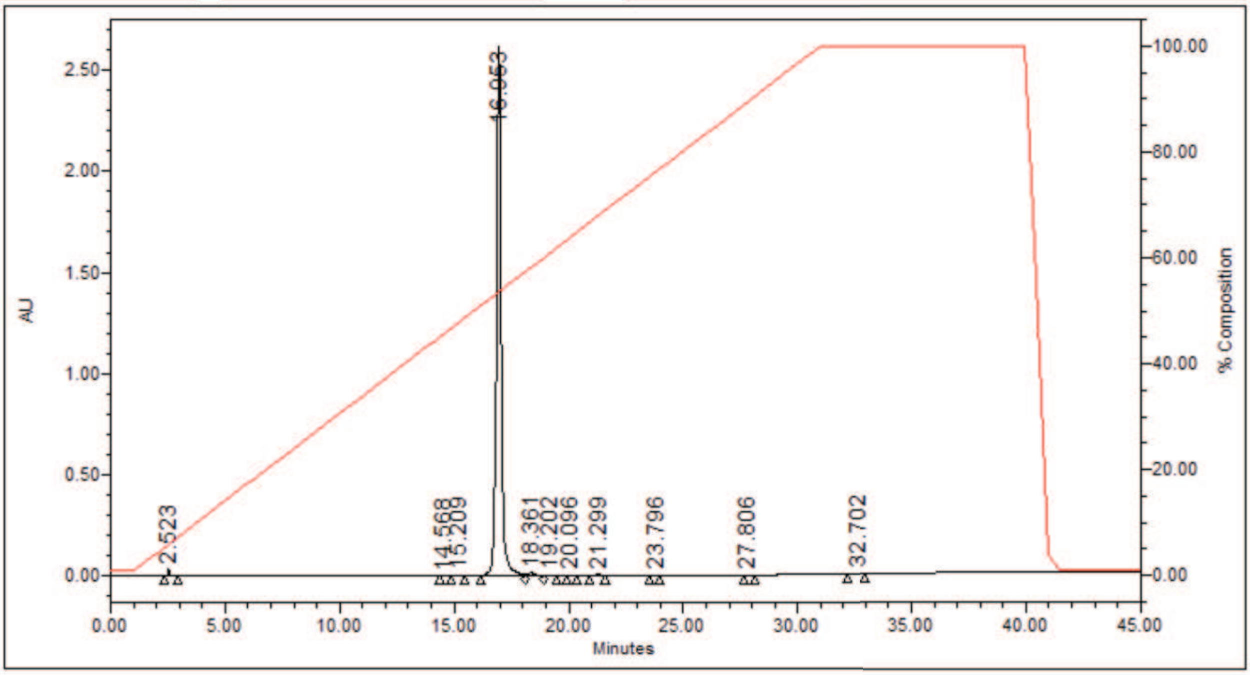

Peak information

	RT	Area	% Area
1	2.523	225067	0.57
2	14.568	6840	0.02
3	15.209	16483	0.04
4	16.953	38132412	97.08
5	18.361	275013	0.70
6	19.202	45092	0.11
7	20.096	9684	0.02
8	21.299	74739	0.19
9	23.796	8439	0.02
10	27.806	5202	0.01
11	32.702	34159	0.09

	RT	Area	% Area
12	46.258	445649	1.13

Figure 9: HPLC trace of compound **1g**

Project Name:	P2X7_Hybrids	Acq. Method Set:	JD_0to100ov30 HPLC4
Sample Name:	NLD35	Sample Set Name:	240703_NLD34NLD36
Injection Volume:	10.00 ul	Wavelength W2489 ChA 254nm	
Run Time:	52.0 Minutes		

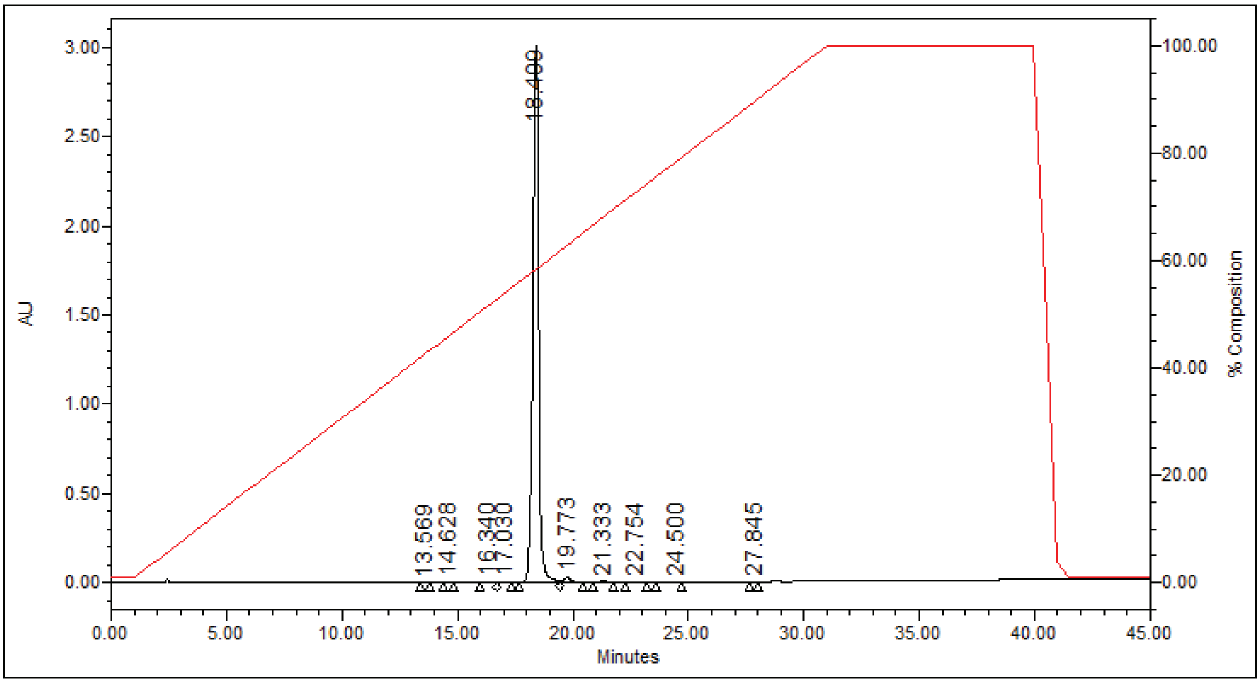

Peak information

	RT	Area	% Area
1	13.569	7884	0.01
2	14.628	11611	0.02
3	16.340	26551	0.05
4	17.030	21244	0.04
5	18.409	53272393	98.60
6	19.773	528749	0.98
7	21.333	120737	0.22
8	22.754	23717	0.04
9	24.500	11662	0.02
10	27.845	5566	0.01

Figure 10: HPLC trace of compound **1h**

Project Name:	P2X7_Hybrids	Acq. Method Set:	JD_0to100ov30 HPLC4
Sample Name:	NLD32	Sample Set Name:	240806_NLC41to44_NLD24_NLD32
Injection Volume:	10.00 ul		
Run Time:	52.0 Minutes		

Wavelength W2489 ChA 254nm

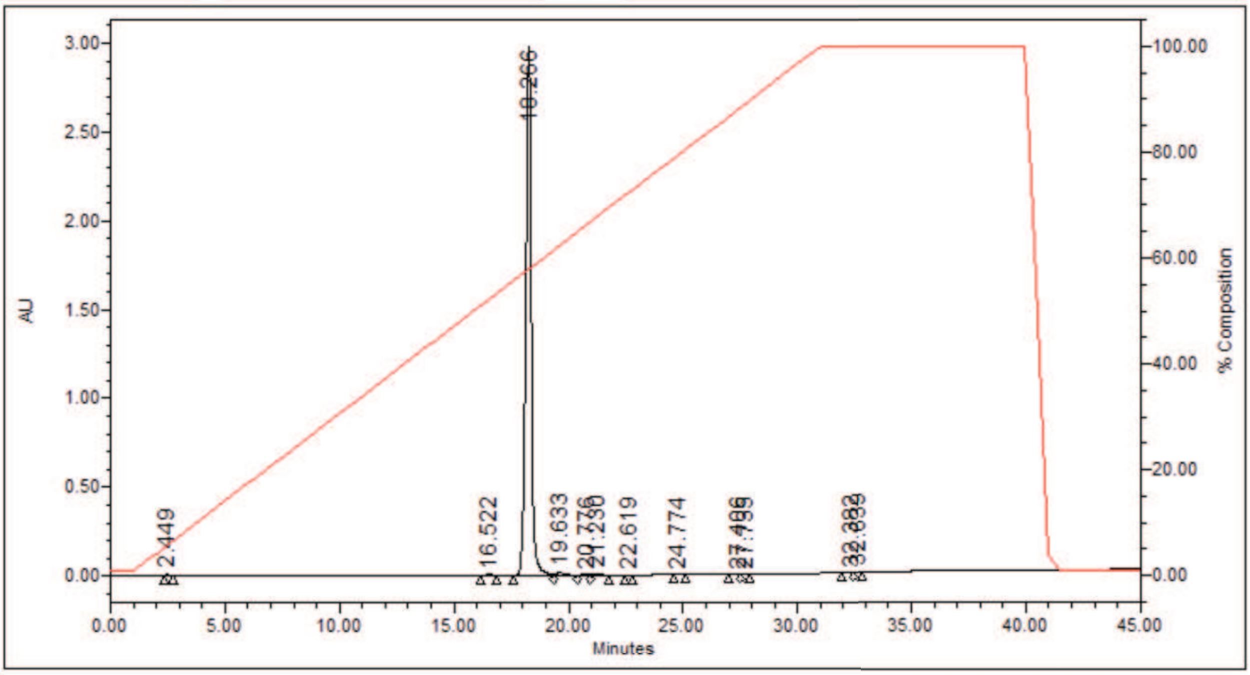

Peak information

	RT	Area	% Area
1	2.449	91615	0.18
2	16.522	105245	0.21
3	18.266	49910678	98.27
4	19.633	369793	0.73
5	20.776	75000	0.15
6	21.230	180396	0.36
7	22.619	4767	0.01
8	24.774	4443	0.01
9	27.406	6492	0.01
10	27.735	10013	0.02
11	32.382	13757	0.03
12	32.639	15723	0.03

Figure 11: HPLC trace of compound **1i**

Project Name:	P2X7_Hybrids	Acq. Method Set:	JD_0to100ov30 HPLC4
Sample Name:	NLD36	Sample Set Name:	240703_NLD34NLD36
Injection Volume:	10.00 ul	**Wavelength W2489 ChA 254nm**	
Run Time:	53.0 Minutes		

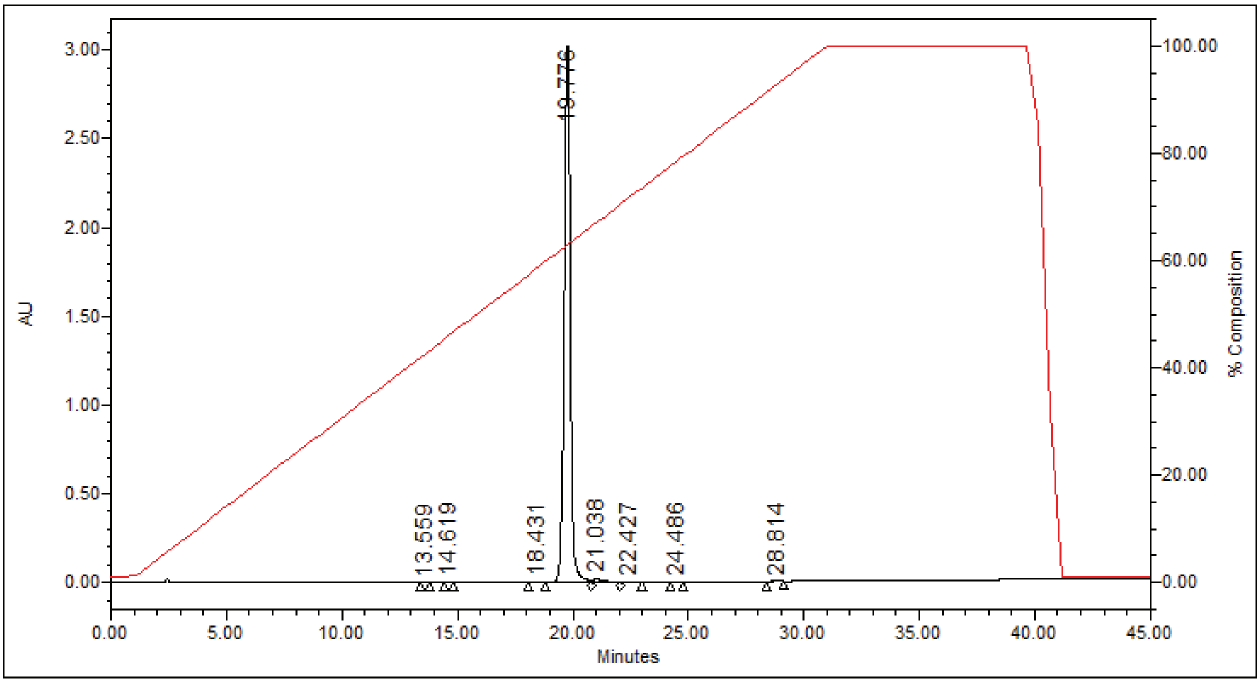

Peak information

	RT	Area	% Area
1	13.559	6839	0.01
2	14.619	7742	0.01
3	18.431	38493	0.07
4	19.776	54747954	98.64
5	21.038	548938	0.99
6	22.427	74895	0.13
7	24.486	21576	0.04
8	28.814	57155	0.10

Figure 12: HPLC trace of compound **1j**

Project Name:	P2X7_Hybrids	Acq. Method Set:	JD_0to100ov30 HPLC4
Sample Name:	NLD33b	Sample Set Name:	240626_NLD31NLD32NLD33
Injection Volume:	10.00 ul		
Run Time:	53.0 Minutes		

Wavelength W2489 ChA 254nm

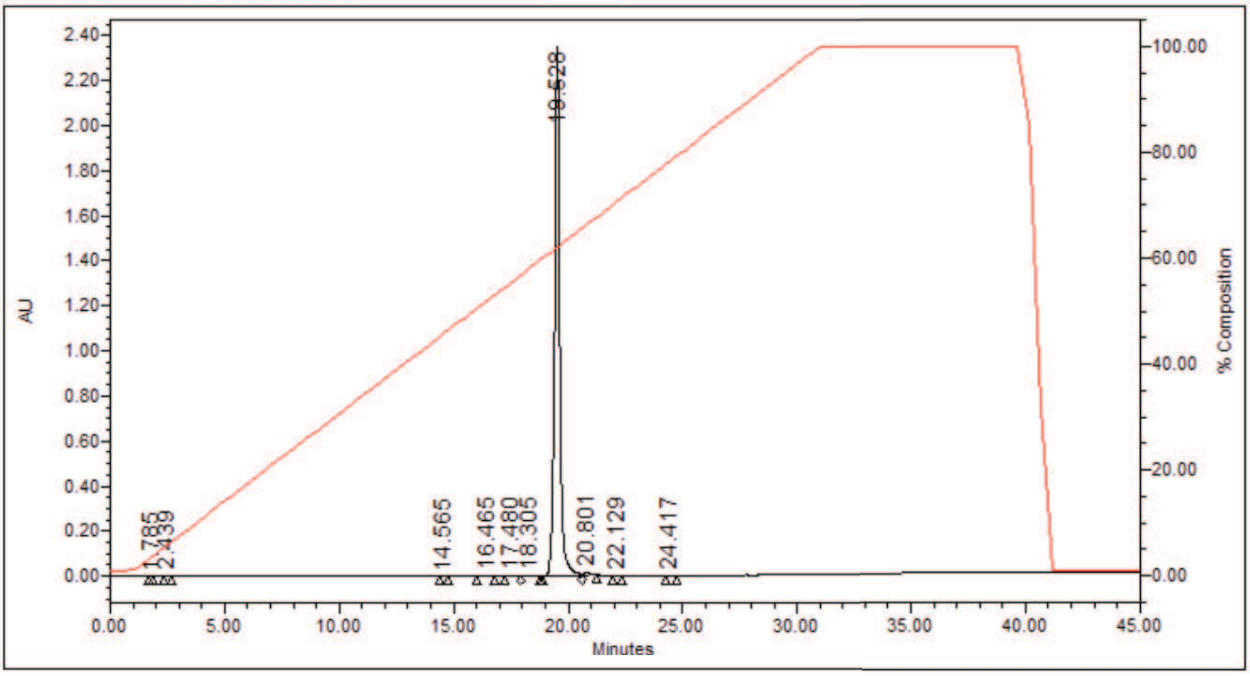

Peak information

	RT	Area	% Area
1	1.785	3638	0.01
2	2.439	33610	0.09
3	14.565	4934	0.01
4	16.465	62931	0.17
5	17.480	10079	0.03
6	18.305	42792	0.12
7	19.528	36596520	99.01
8	20.801	192704	0.52
9	22.129	7662	0.02
10	24.417	5868	0.02

Figure 13: HPLC trace of compound **1k**

Project Name:	P2X7_Hybrids		Acq. Method Set:	JD_0to100ov30 HPLC4
Sample Name:	NLD39		Sample Set Name:	240718_NLD24e_NLD39
Injection Volume:	10.00 ul		**Wavelength W2489 ChA 254nm**	
Run Time:	53.0 Minutes			

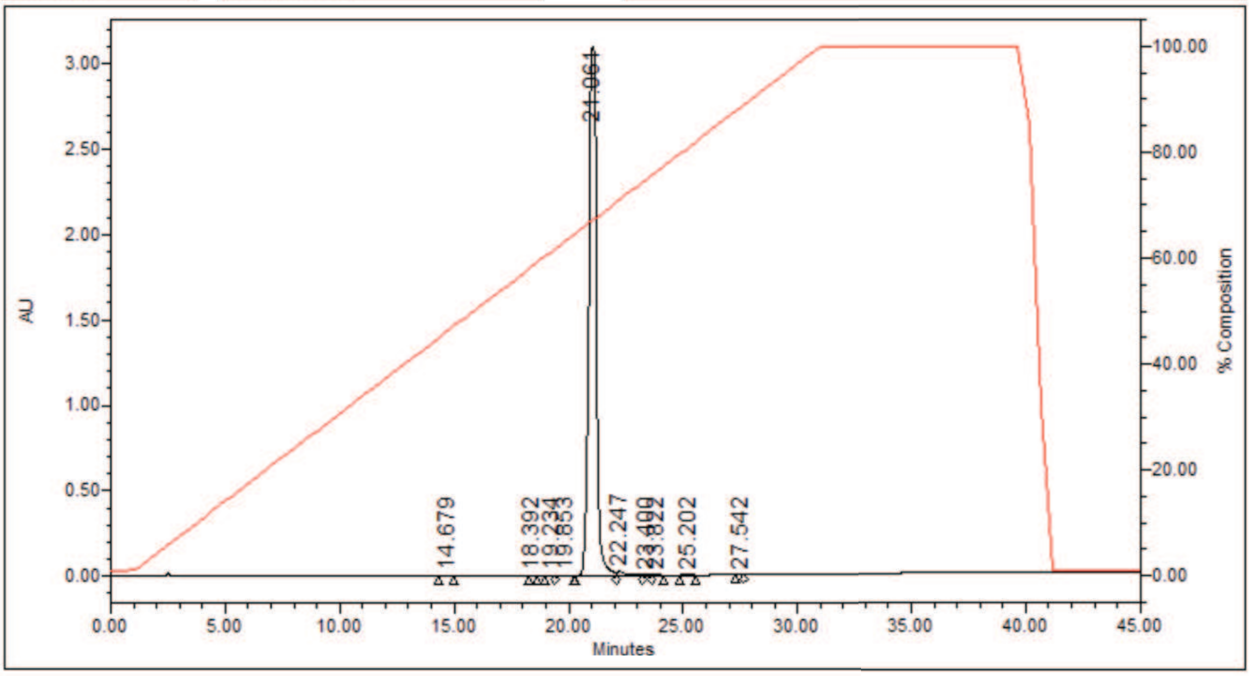

Peak information

	RT	Area	% Area
1	14.679	40797	0.06
2	18.392	5120	0.01
3	19.234	11488	0.02
4	19.853	61201	0.08
5	21.061	72858880	98.75
6	22.247	623427	0.85
7	23.400	48244	0.07
8	23.822	43738	0.06
9	25.202	72289	0.10
10	27.542	12705	0.02

Figure 14: HPLC trace of compound **1l**

Project Name:	P2X7_Hybrids	Acq. Method Set:	JD_0to100ov30 HPLC4
Sample Name:	NLD37	Sample Set Name:	240712_NLD37_repeat
Injection Volume:	10.00 ul		
Run Time:	53.0 Minutes		

Wavelength W2489 ChA 254nm

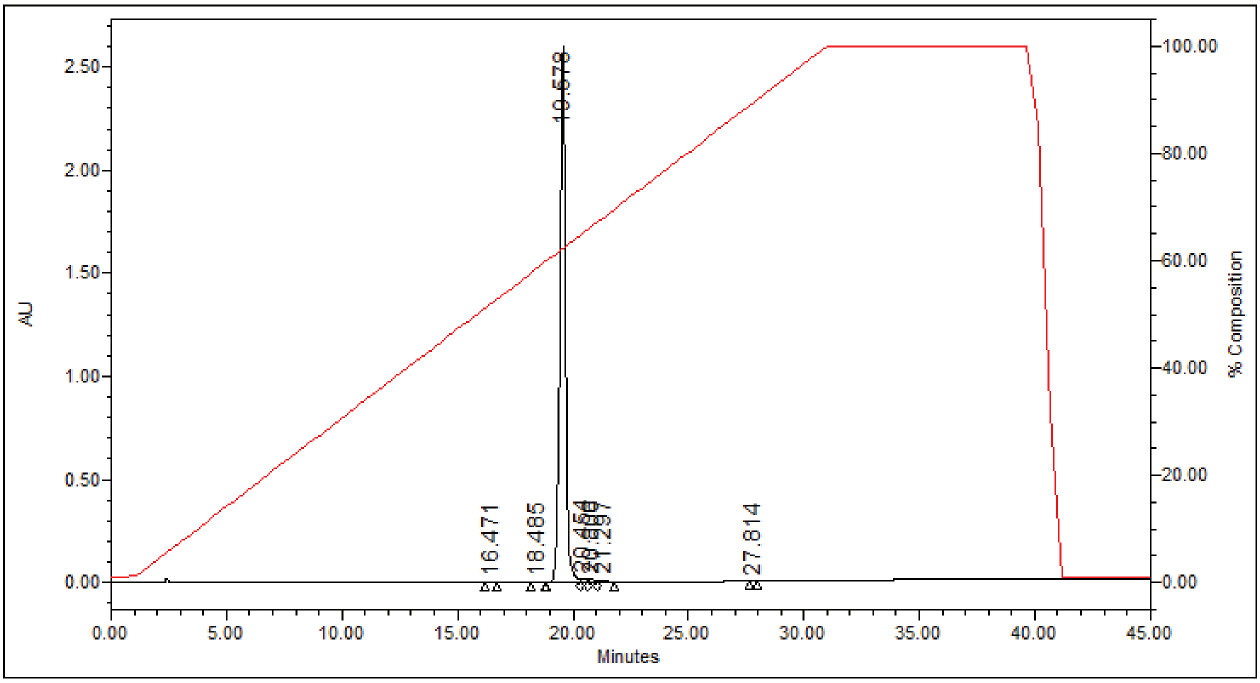

Peak information

	RT	Area	% Area
1	16.471	13217	0.03
2	18.485	23573	0.05
3	19.578	44125477	98.42
4	20.454	300287	0.67
5	20.806	200740	0.45
6	21.297	163962	0.37
7	27.814	4335	0.01

Figure 15: HPLC trace of compound **1m**

Project Name:	P2X7_Hybrids	Acq. Method Set:	JD_0to100ov30 HPLC4
Sample Name:	NLD46	Sample Set Name:	240905_NLD46_NLC41_NLC43
Injection Volume:	10.00 ul		
Run Time:	50.0 Minutes		

Wavelength W2489 ChA 254nm

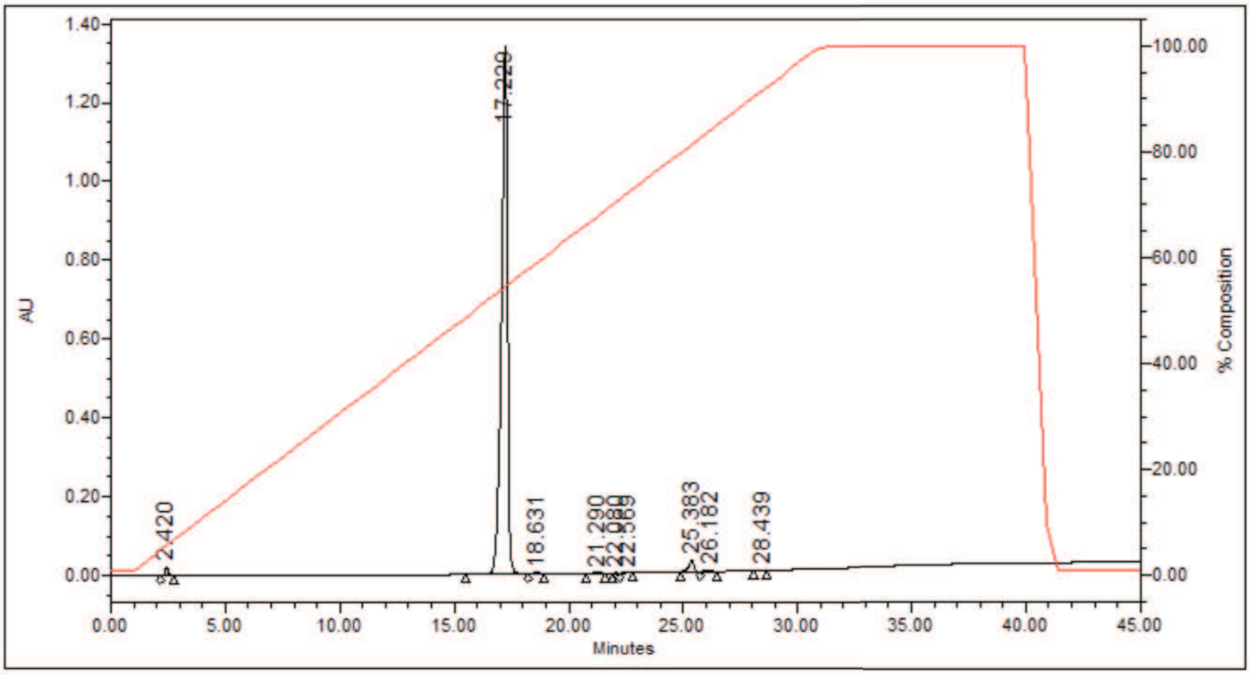

Peak information

	RT	Area	% Area
1	2.420	185812	0.73
2	17.229	24550483	96.52
3	18.631	66327	0.26
4	21.290	59057	0.23
5	22.080	3325	0.01
6	22.569	15332	0.06
7	25.383	475053	1.87
8	26.182	67100	0.26
9	28.439	13072	0.05

Figure 16: HPLC trace of compound **1n**

Project Name:	P2X7_Hybrids	Acq. Method Set:	JD_0to100ov30 HPLC4
Sample Name:	NLD50	Sample Set Name:	250320_NLE11c_NLD50
Injection Volume:	5.00 ul		
Run Time:	50.0 Minutes		

Wavelength W2489 ChA 254nm

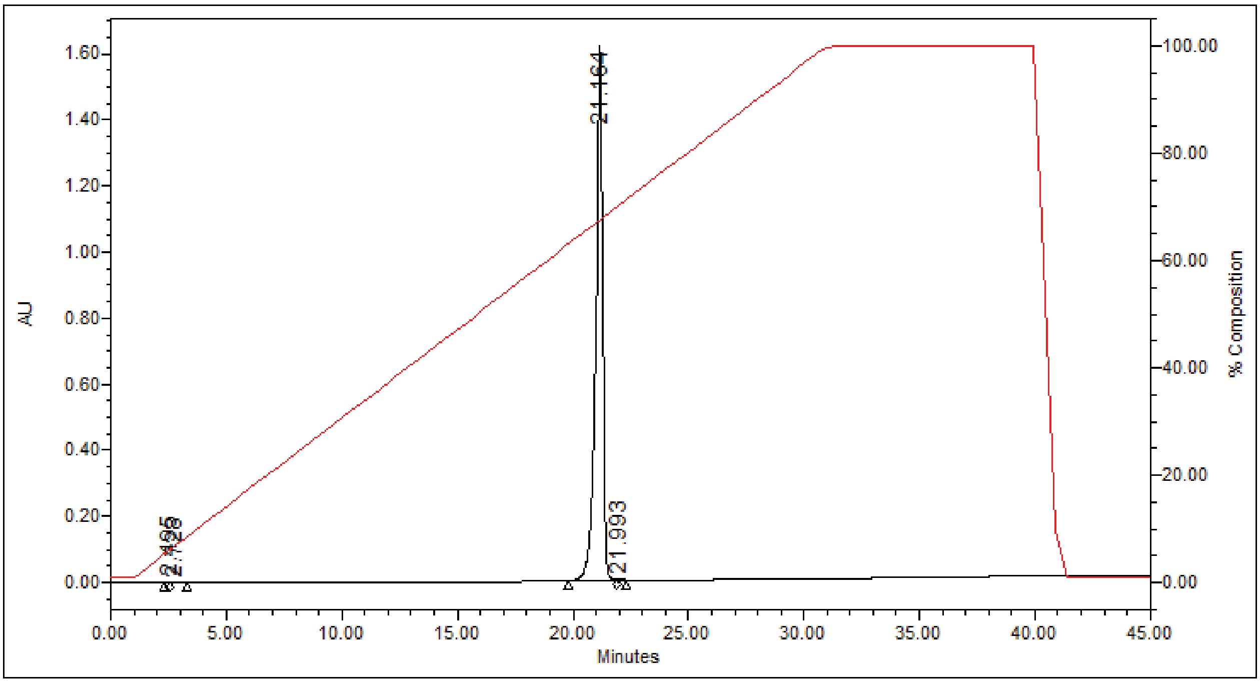

Peak information

	RT	Area	% Area
1	2.495	7981	0.02
2	2.728	12870	0.04
3	21.164	34026216	98.92
4	21.993	35267	0.10
5	46.208	315747	0.92

Figure 17: HPLC trace of compound **1o**

Project Name:	P2X7_Hybrids	Acq. Method Set:	JD_0to100ov30 HPLC4
Sample Name:	NLC44 F14-15	Sample Set Name:	290824_NLC44
Injection Volume:	10.00 ul		
Run Time:	50.0 Minutes		

Wavelength W2489 ChA 254nm

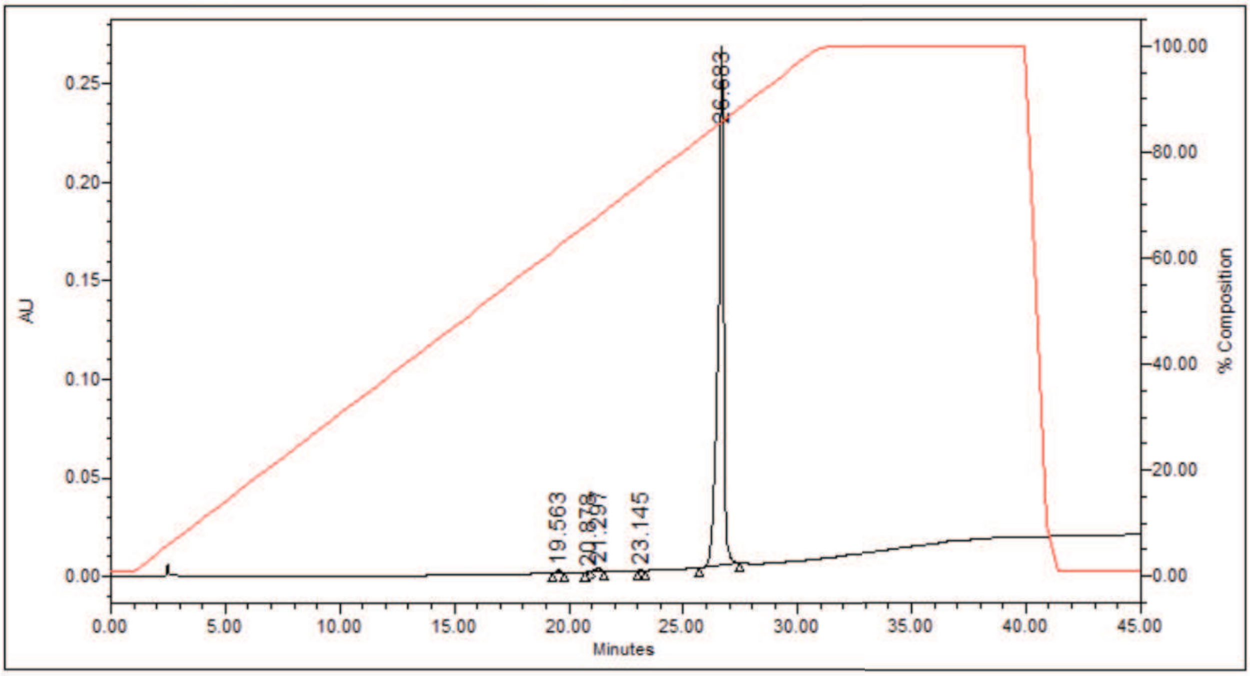

Peak information

	RT	Area	% Area
1	19.563	13972	0.31
2	20.878	3259	0.07
3	21.297	31760	0.71
4	23.145	2897	0.06
5	26.683	4438964	98.84

Figure 18: HPLC trace of compound **2a**

Project Name:	P2X7_Hybrids	Acq. Method Set:	JD_0to100ov30 HPLC4
Sample Name:	NLC17	Sample Set Name:	240415_NLD28_NLC16_NLC17
Injection Volume:	10.00 ul		
Run Time:	53.0 Minutes		

Wavelength W2489 ChA 254nm

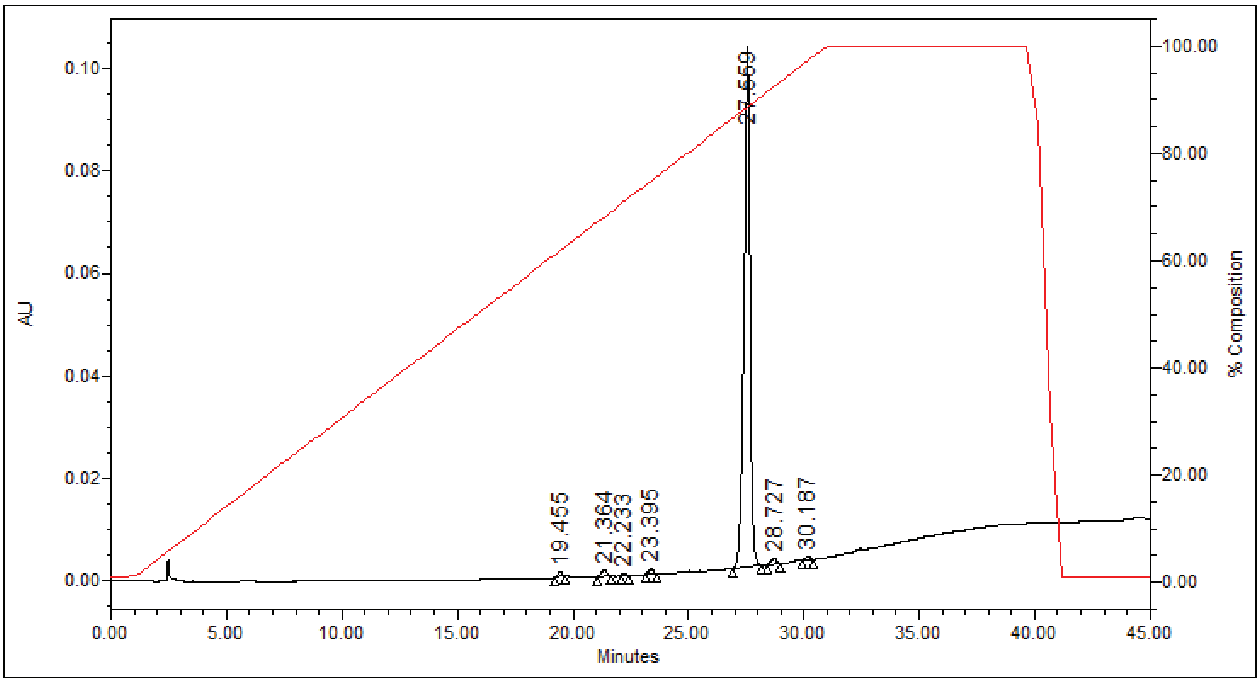

Peak information

	RT	Area	% Area
1	19.455	10862	0.57
2	21.364	19687	1.04
3	22.233	4698	0.25
4	23.395	14216	0.75
5	27.559	1813447	95.87
6	28.727	19226	1.02
7	30.187	9473	0.50

Figure 19: HPLC trace of compound **2b**

Project Name:	P2X7_Hybrids		Acq. Method Set:	JD_0to100ov30 HPLC4
Sample Name:	NLD49_F4_5		Sample Set Name:	20241014_CD
Injection Volume:	10.00 ul		**Wavelength W2489 ChA 254nm**	
Run Time:	52.0 Minutes			

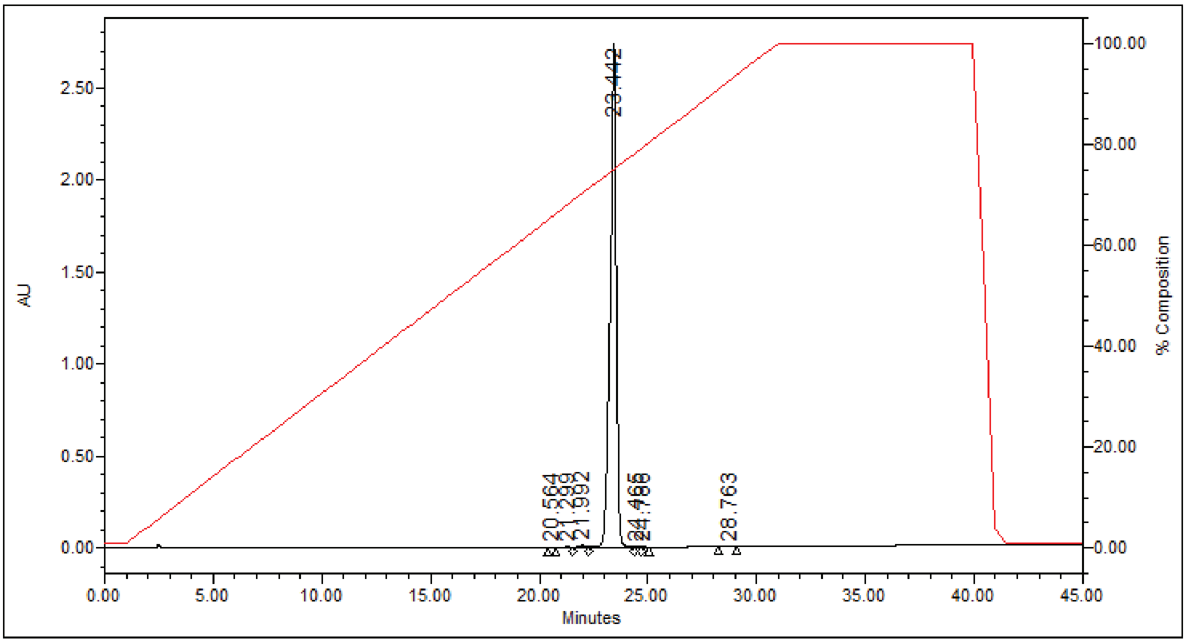

Peak information

	RT	Area	% Area
1	20.564	6216	0.01
2	21.299	78992	0.15
3	21.992	222651	0.41
4	23.442	53982226	99.23
5	24.465	43412	0.08
6	24.786	17260	0.03
7	28.763	49842	0.09

Figure 20: HPLC trace of compound **2c**

Project Name:	P2X7_Hybrids	Acq. Method Set:	JD_0to100ov30 HPLC4
Sample Name:	NLC27 F7&8	Sample Set Name:	240523_NLC27andDane
Injection Volume:	10.00 ul		
Run Time:	53.0 Minutes		

Wavelength W2489 ChA 254nm

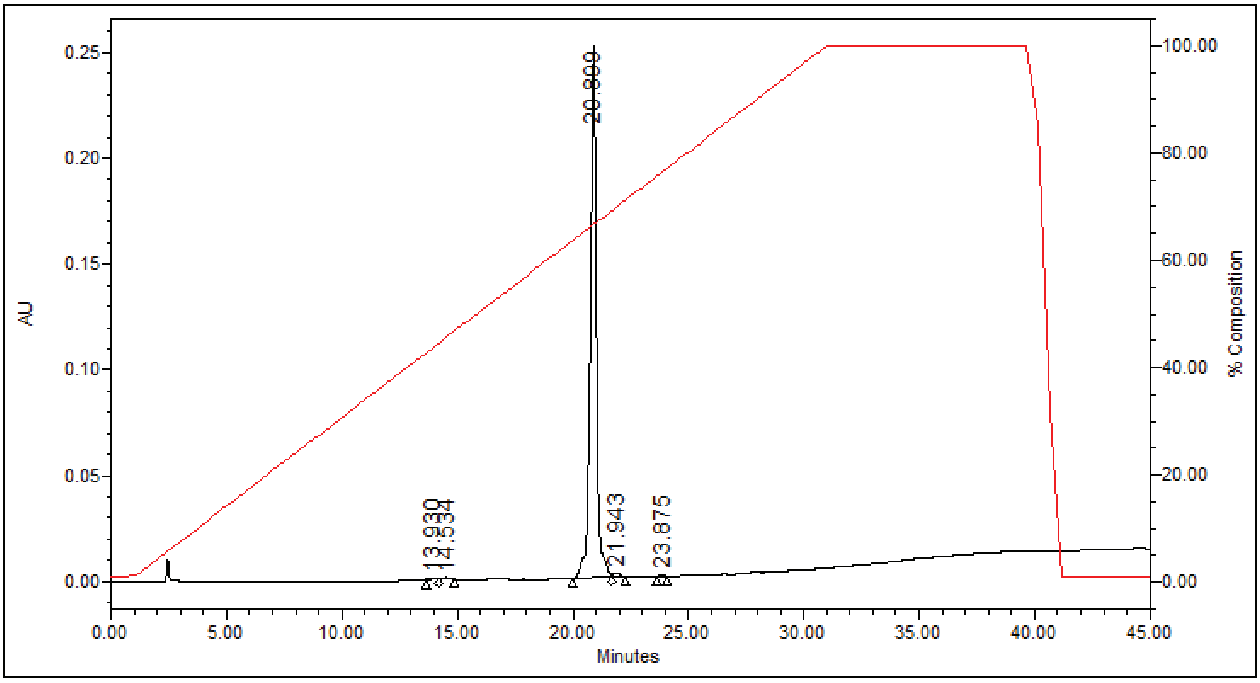

Peak information

	RT	Area	% Area
1	13.930	13960	0.30
2	14.534	16857	0.36
3	20.899	4572777	98.16
4	21.943	46697	1.00
5	23.875	8281	0.18

Figure 21: HPLC trace of compound **2d**

Project Name:	P2X7_Hybrids	Acq. Method Set:	HPLC6_default 0to100ov30 02mL
Sample Name:	NLC42 S2	Sample Set Name:	240909_NLC41_42_43
Injection Volume:	10.00 ul		
Run Time:	50.0 Minutes		

Wavelength PDA 210.0 to 400.0 nm at 4.8 nm

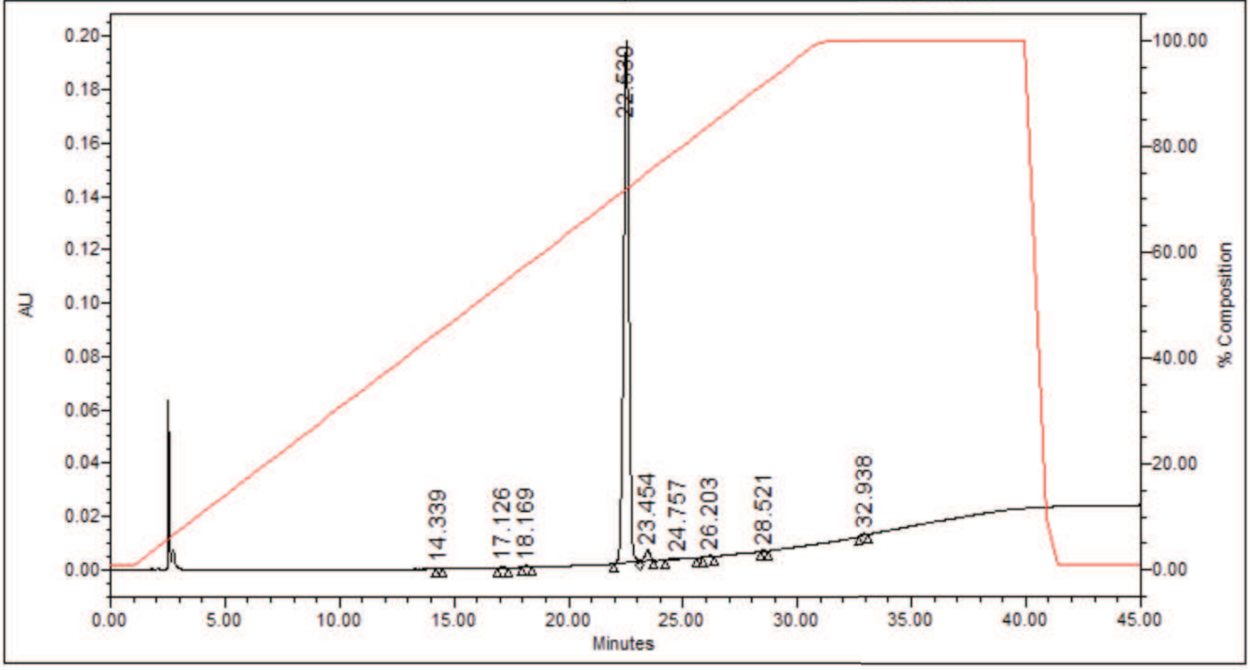

Peak information

	RT	Area	% Area
1	14.339	3629	0.11
2	17.126	8721	0.26
3	18.169	7807	0.24
4	22.530	3198127	96.29
5	23.454	62788	1.89
6	24.757	23814	0.72
7	26.203	5520	0.17
8	28.521	3061	0.09
9	32.938	7813	0.24

Figure 22: HPLC trace of compound **2e**

Project Name:	P2X7_Hybrids	Acq. Method Set:	JD_0to100ov30 HPLC4
Sample Name:	NLC43c_F5-7	Sample Set Name:	240912_NLC43c
Injection Volume:	10.00 ul		
Run Time:	50.0 Minutes		

Wavelength W2489 ChA 254nm

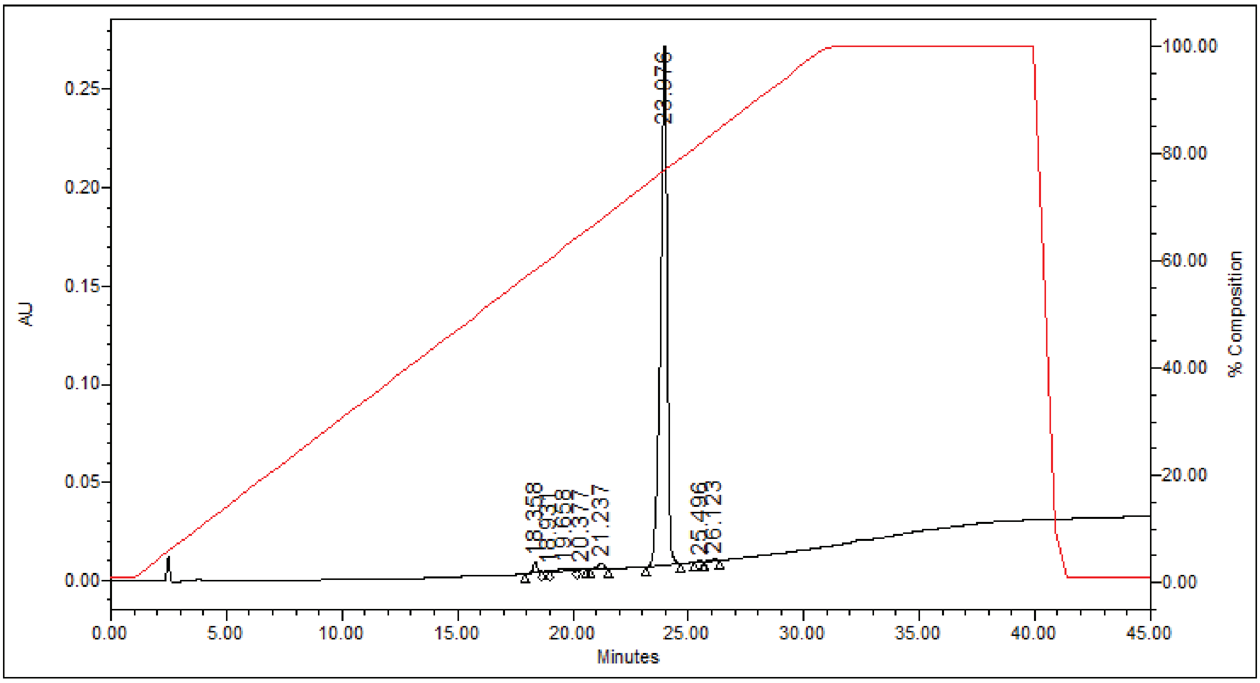

Peak information

	RT	Area	% Area
1	18.358	78482	1.44
2	18.931	14528	0.27
3	19.658	71143	1.30
4	20.377	13212	0.24
5	21.237	59995	1.10
6	23.976	5195025	95.23
7	25.496	7154	0.13
8	26.123	15632	0.29

Figure 23: HPLC trace of compound **2f**

Project Name:	P2X7_Hybrids	Acq. Method Set:	HPLC6_default 0to100ov30 02mL
Sample Name:	NLC41c_F11-12	Sample Set Name:	240911_NLC41NLC43
Injection Volume:	10.00 ul		
Run Time:	50.0 Minutes		

Wavelength PDA 210.0 to 400.0 nm at 4.8 nm

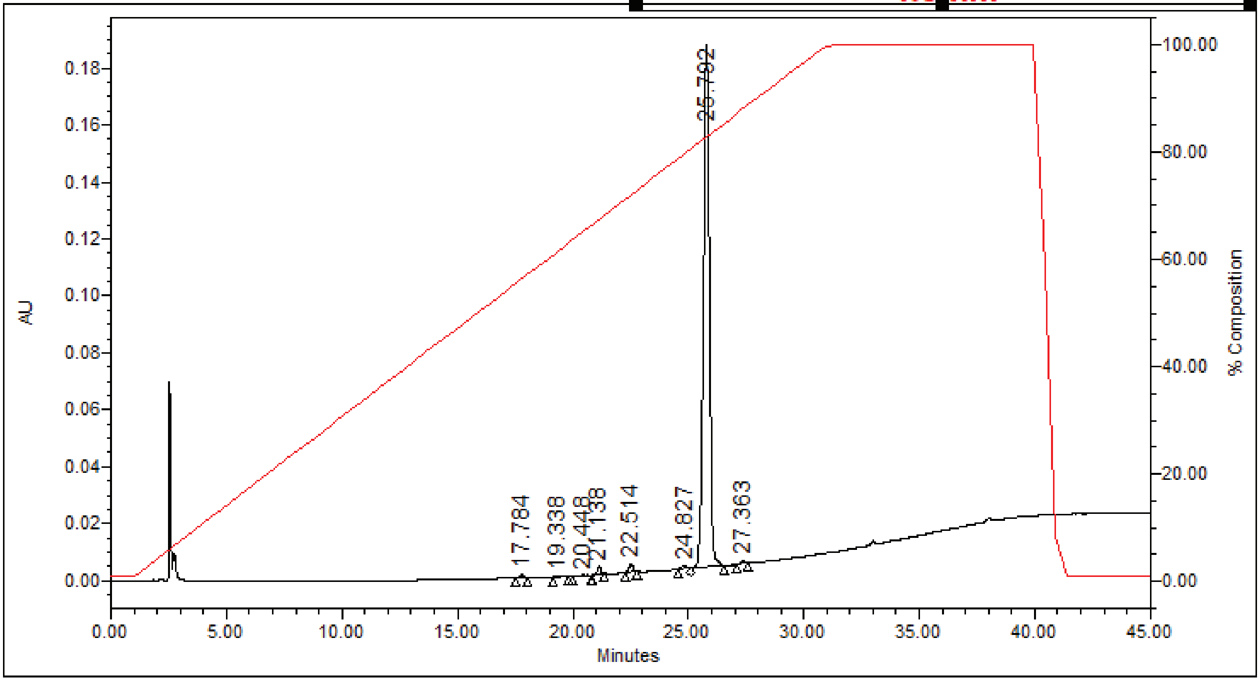

Peak information

	RT	Area	% Area
1	17.784	16100	0.49
2	19.338	5678	0.17
3	20.448	6926	0.21
4	21.138	26972	0.81
5	22.514	32672	0.98
6	24.827	19831	0.60
7	25.792	3195373	96.34
8	27.363	13379	0.40

Figure 24: HPLC trace of compound **2g**

2.6 NMR Spectra of biologically tested compounds

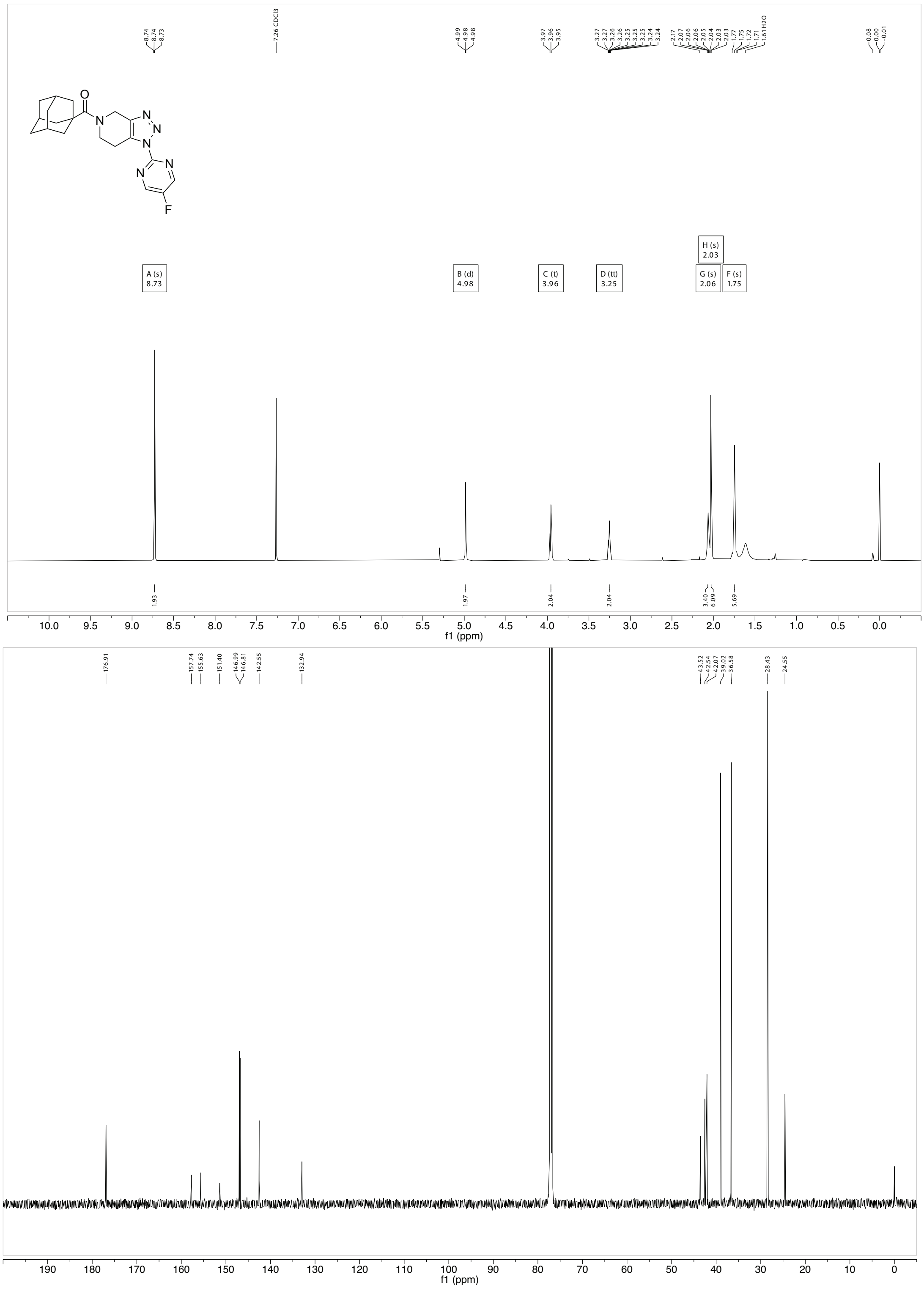

Figure 25: ^{1}H and ^{13}C of compound **1a**

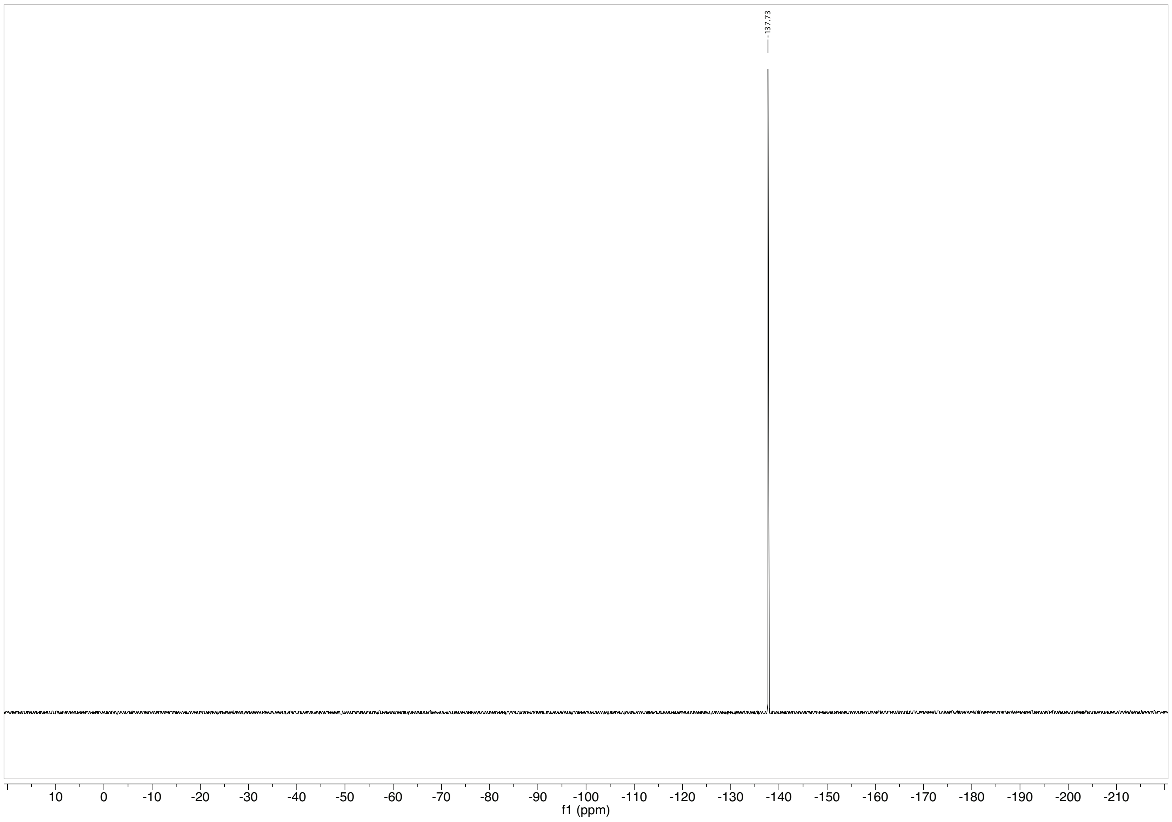

Figure 26: ^{19}F NMR of compound **1a**

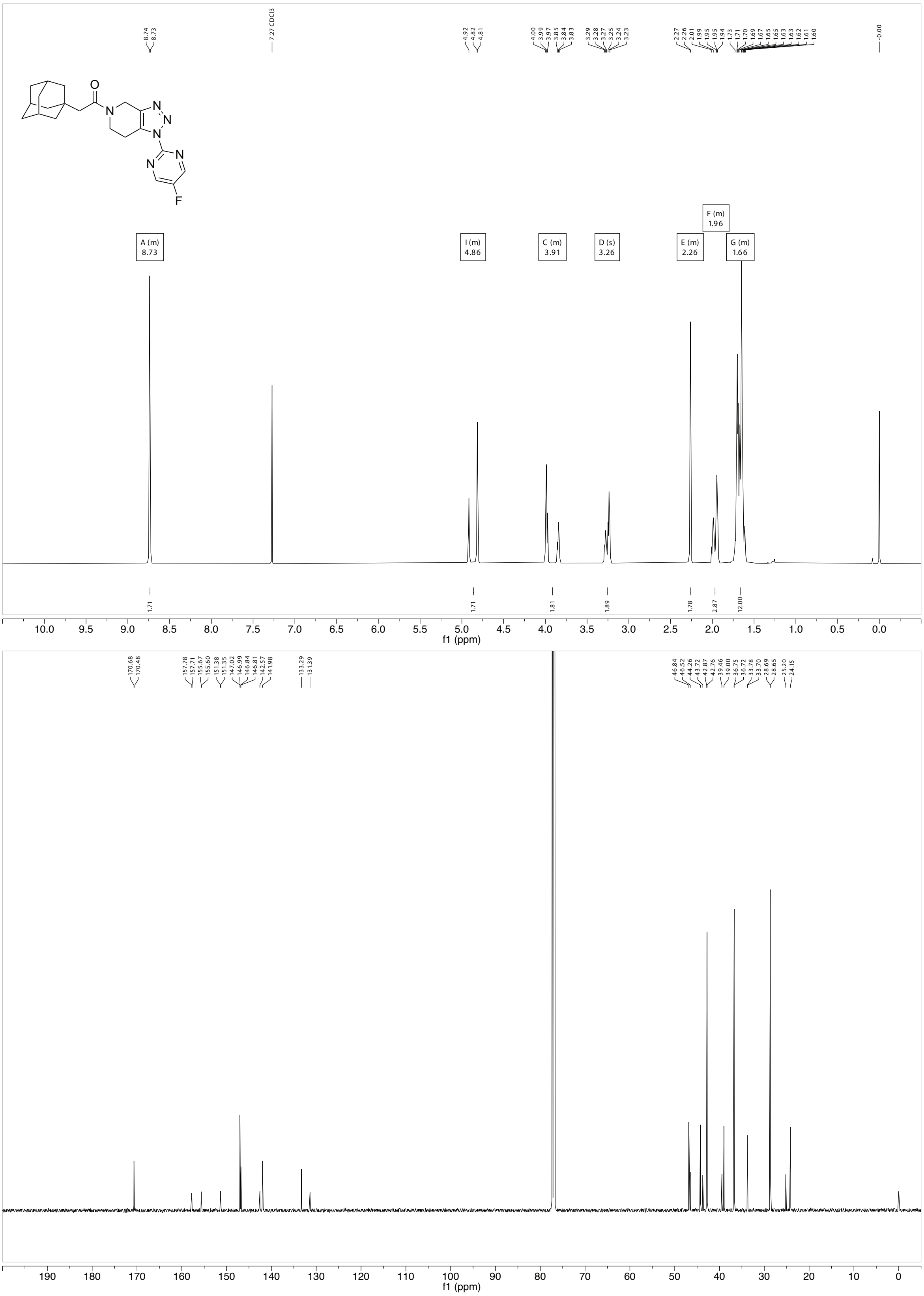

Figure 27: ^{1}H and ^{13}C of compound **1b**

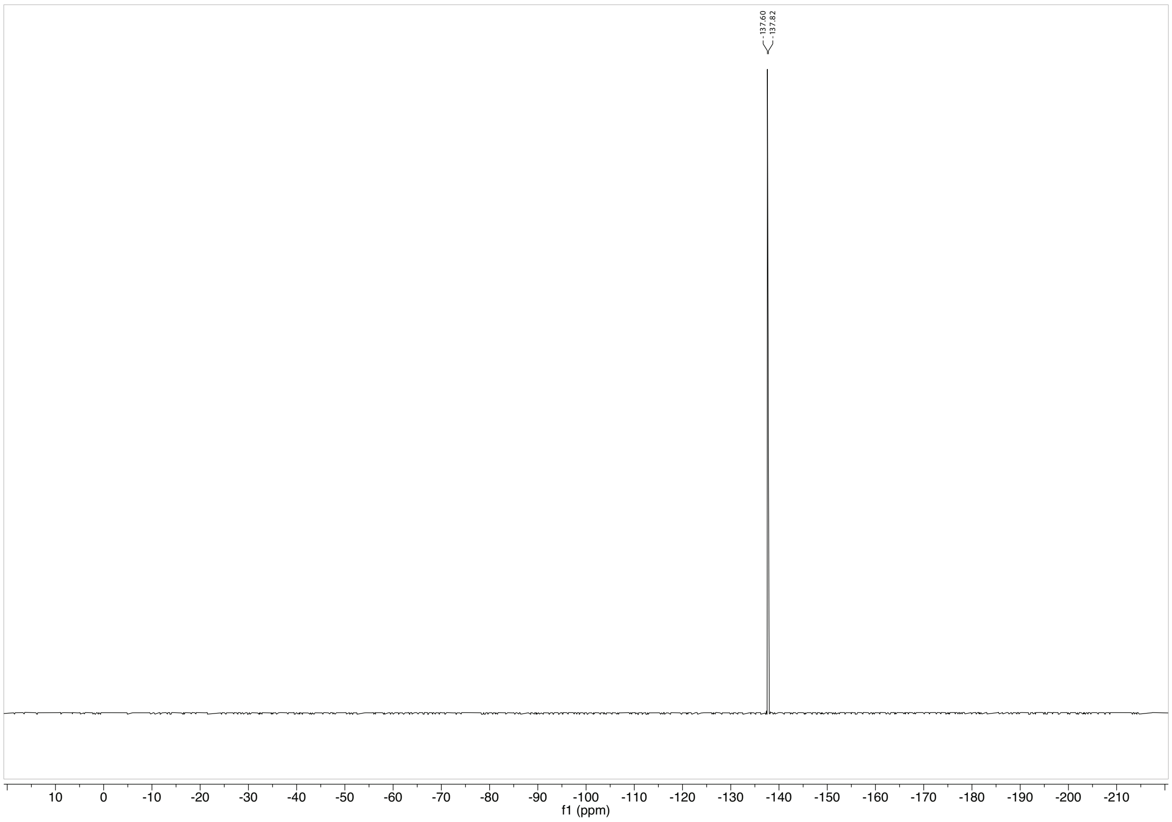

Figure 28: ^{19}F NMR of compound **1b**

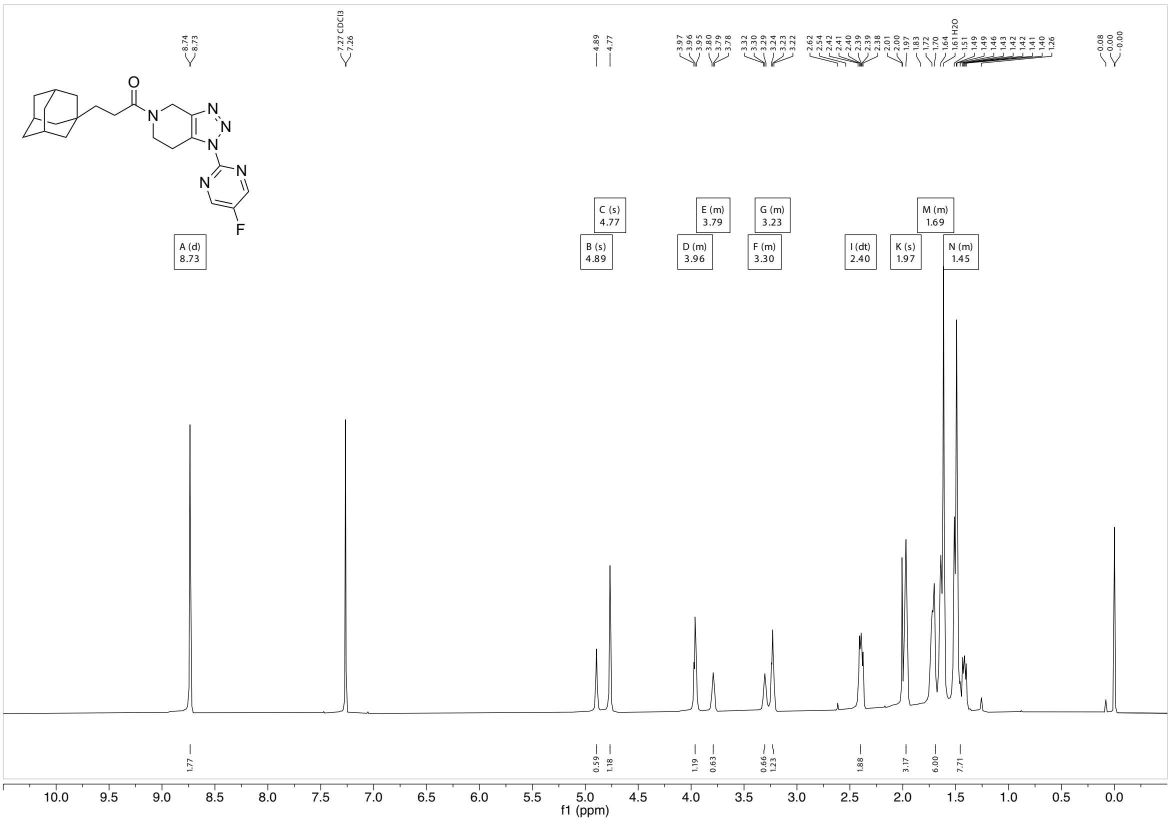

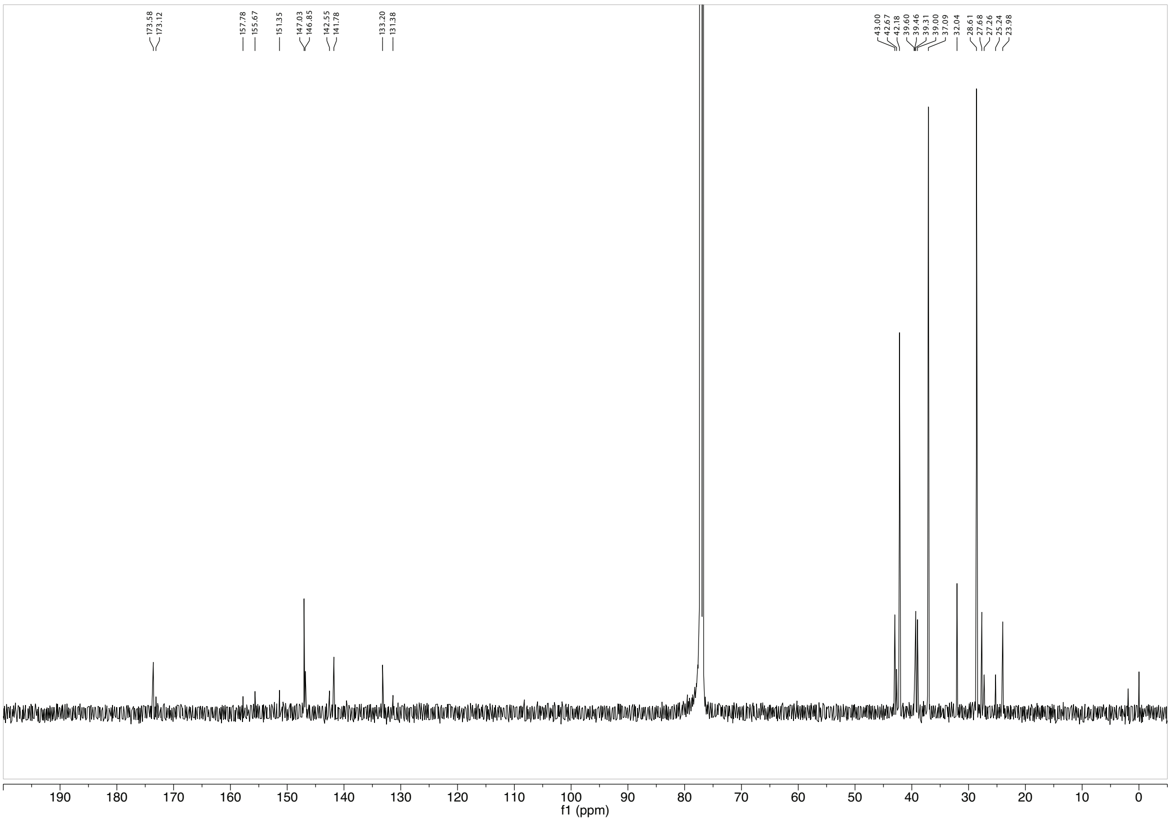

Figure 29: ^{1}H and ^{13}C of compound **1c**

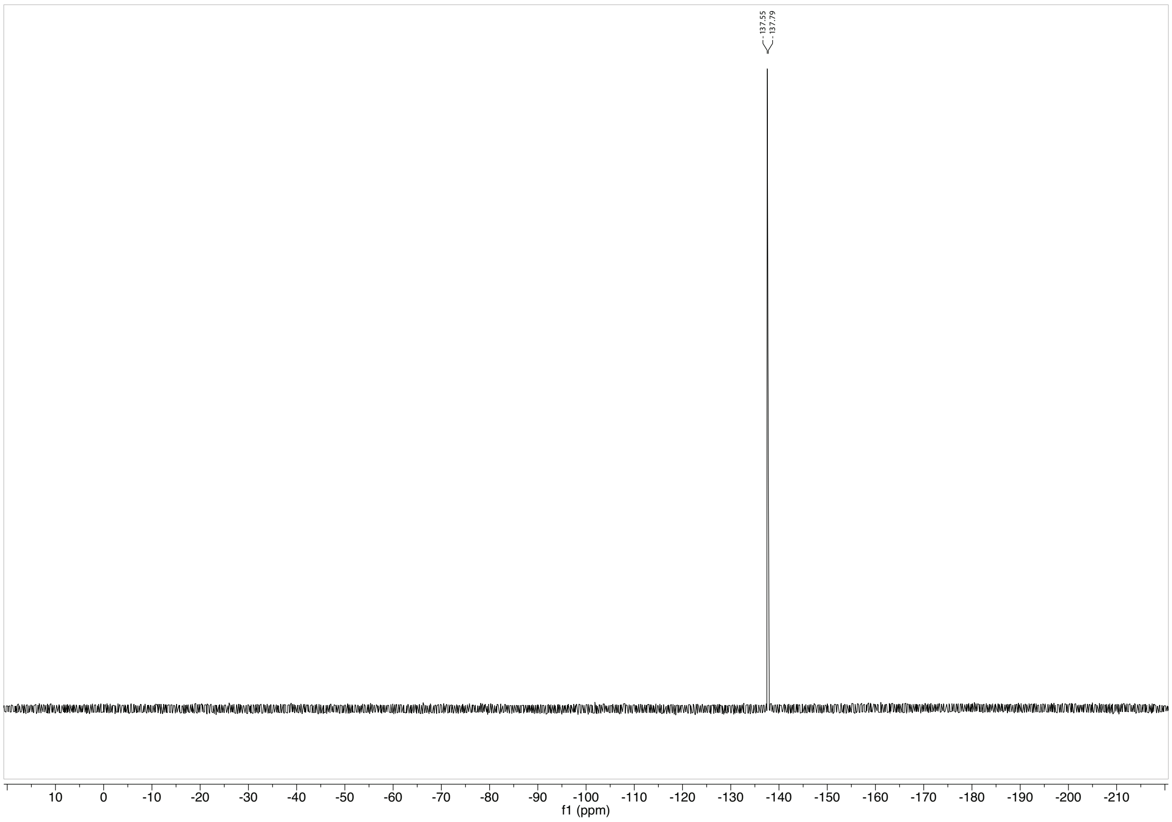

Figure 30: ^{19}F NMR of compound **1c**

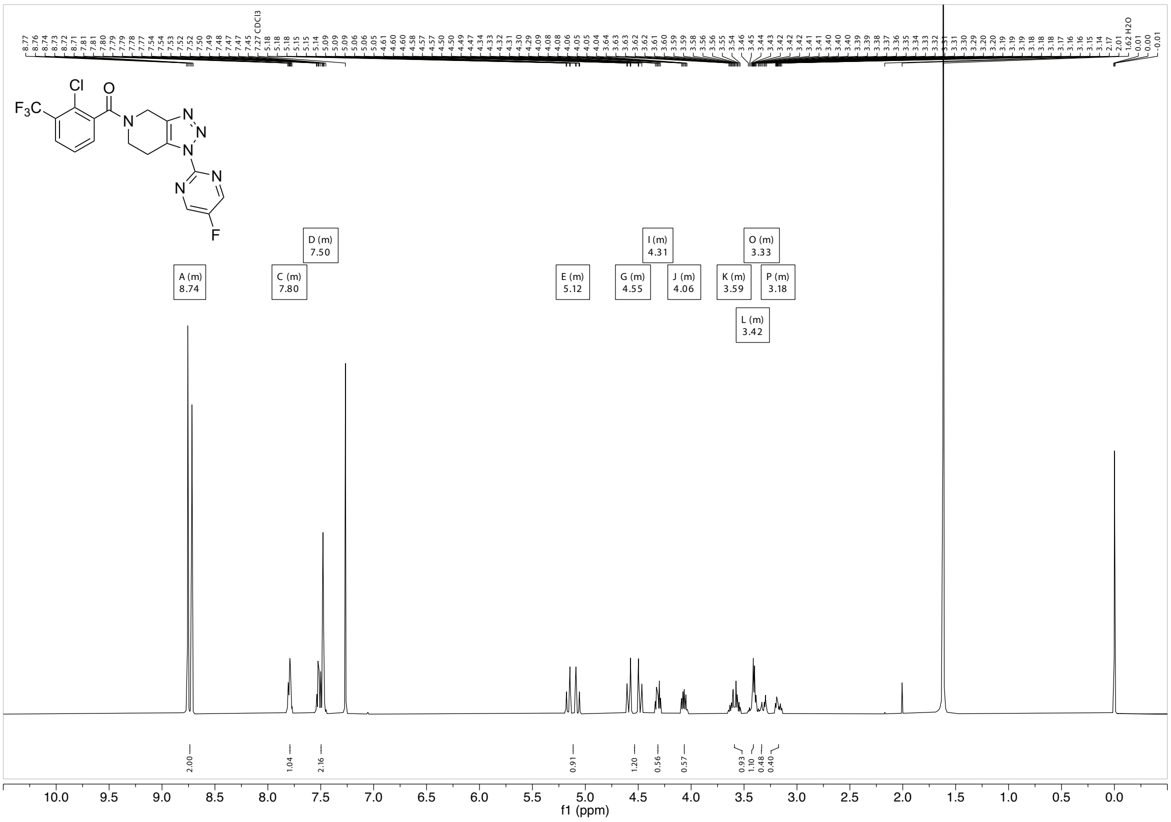

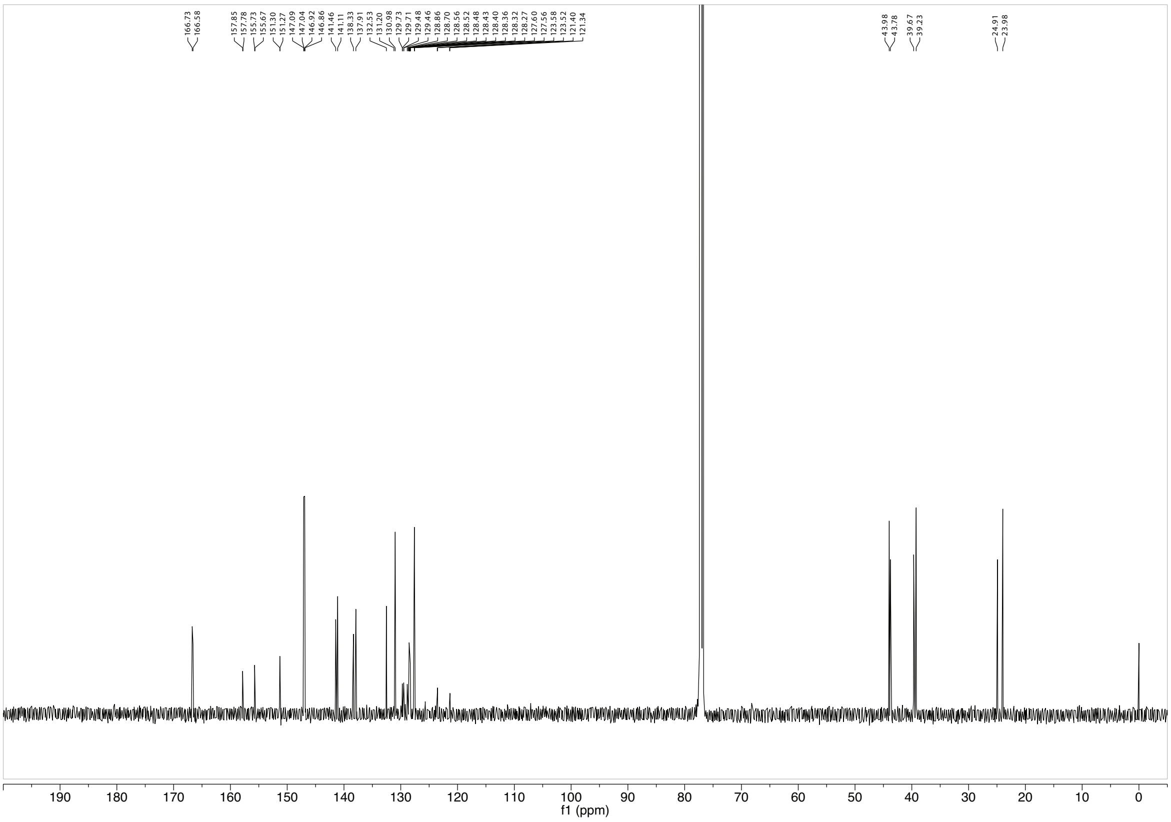

Figure 31: ^{1}H and ^{13}C of compound **1d**

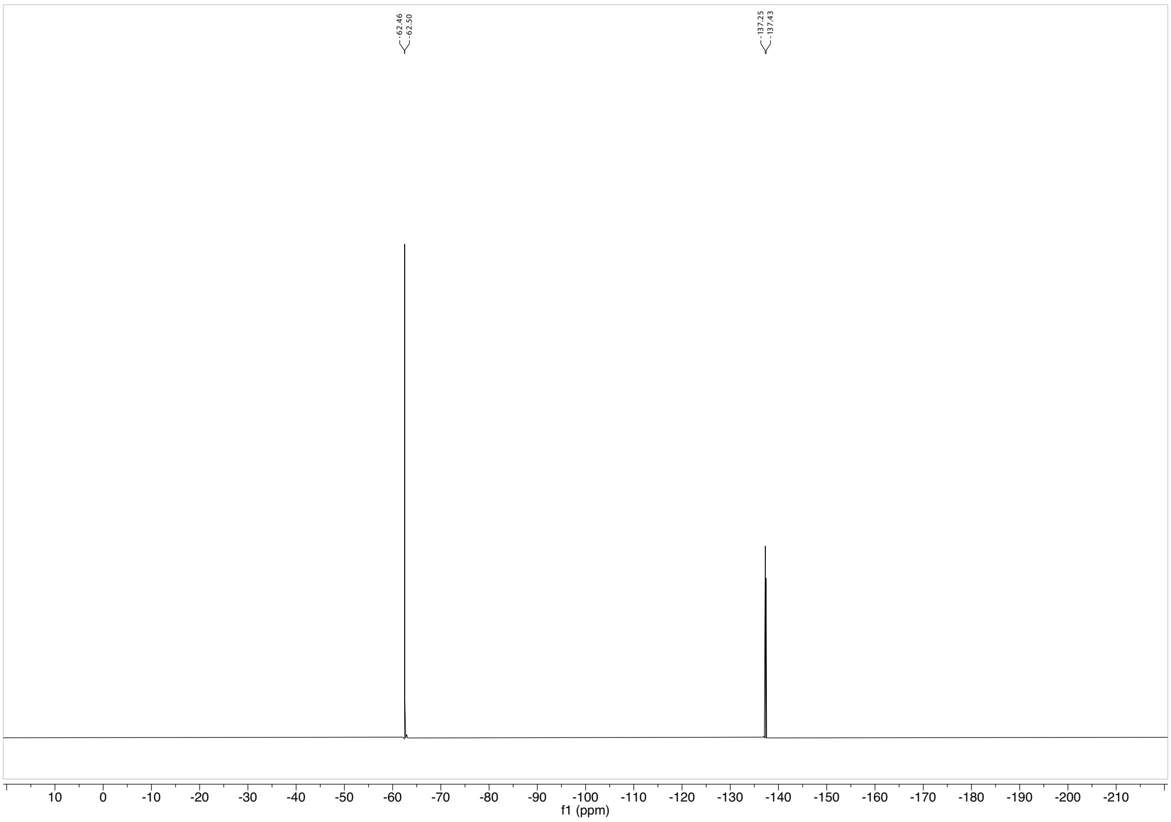

Figure 32: ^{19}F NMR of compound **1d**

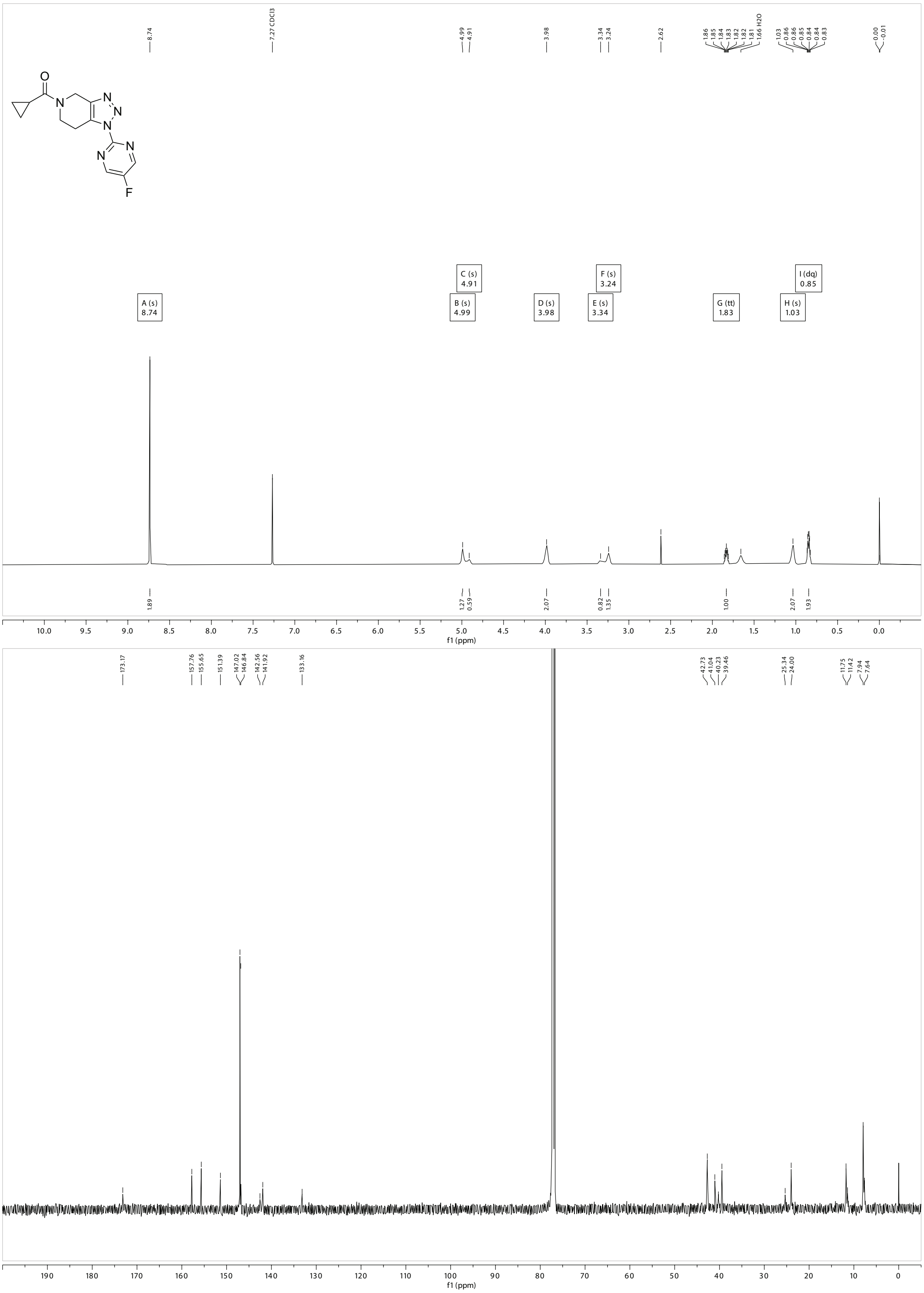

Figure 33: ^{1}H and ^{13}C of compound **1e**

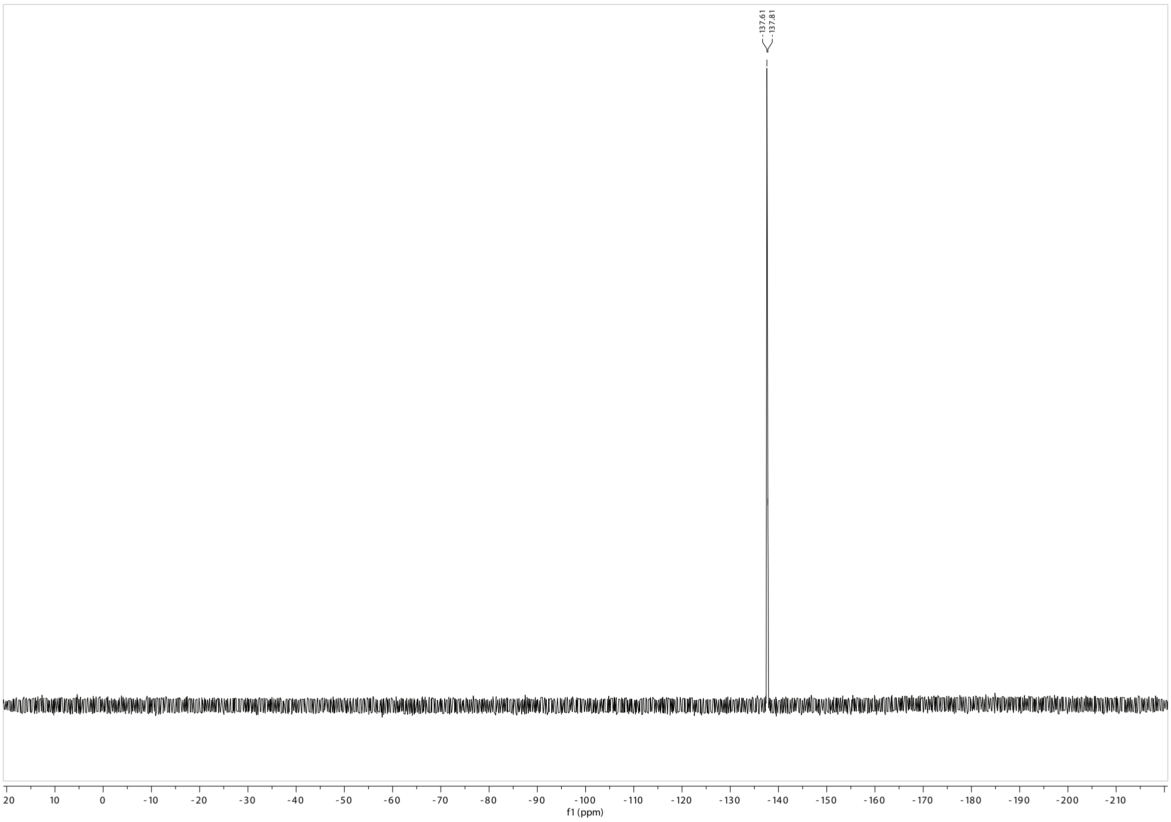

Figure 34: ^{19}F NMR of compound **1e**

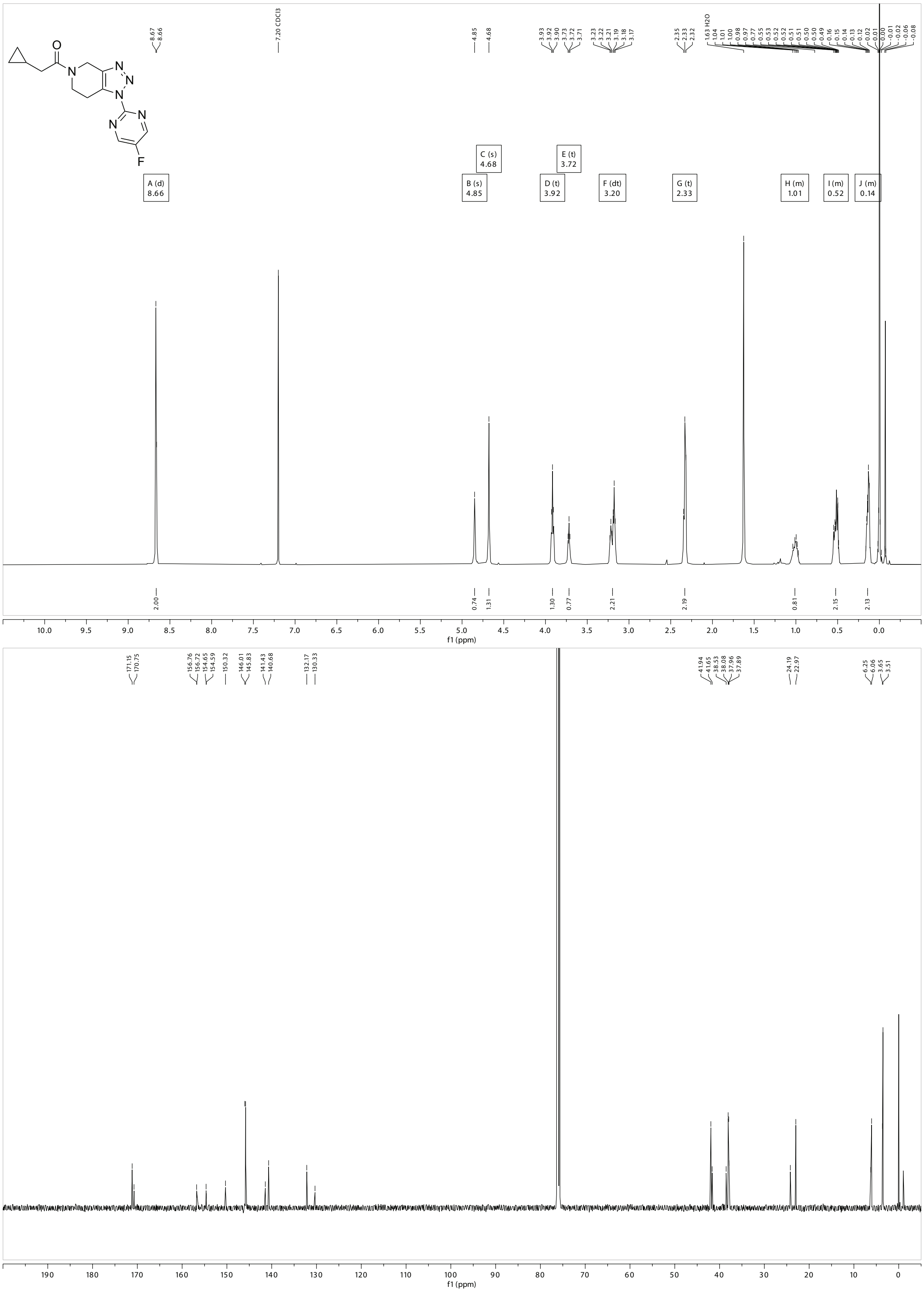

Figure 35: ^{1}H and ^{13}C of compound **1f**

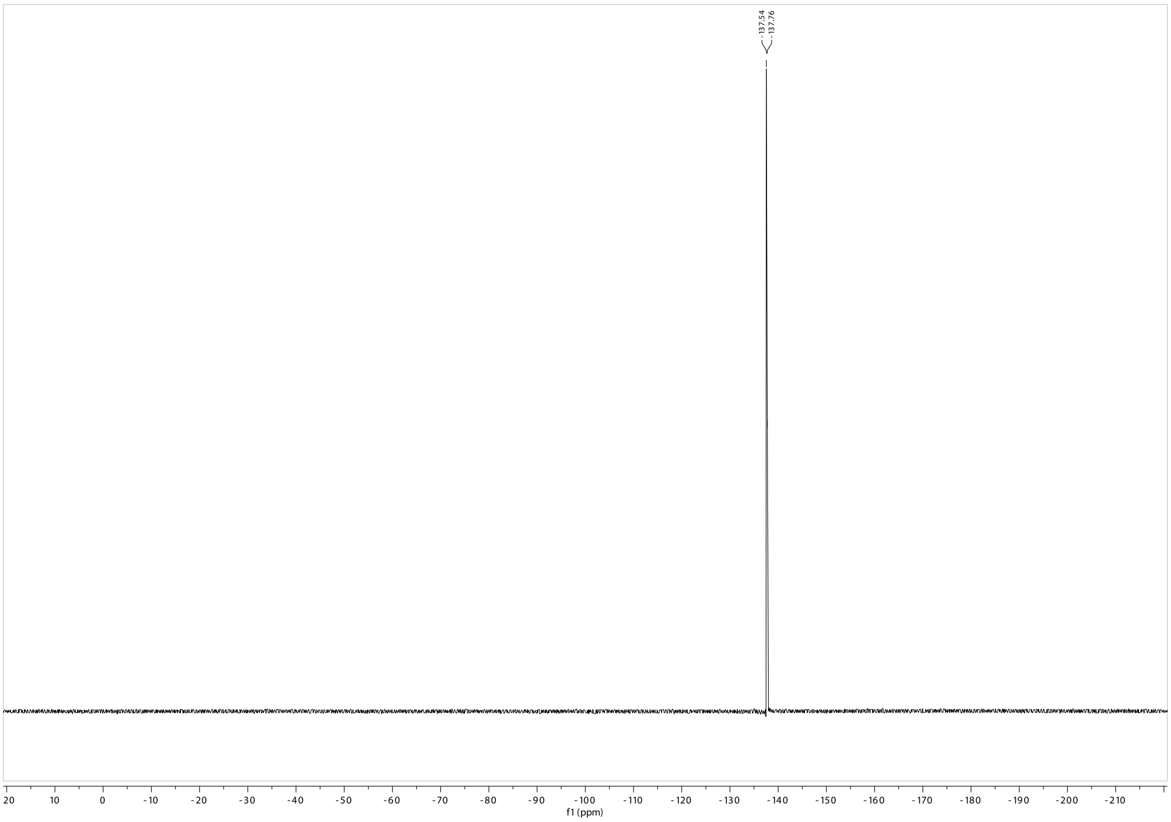

Figure 36: ^{19}F NMR of compound **1f**

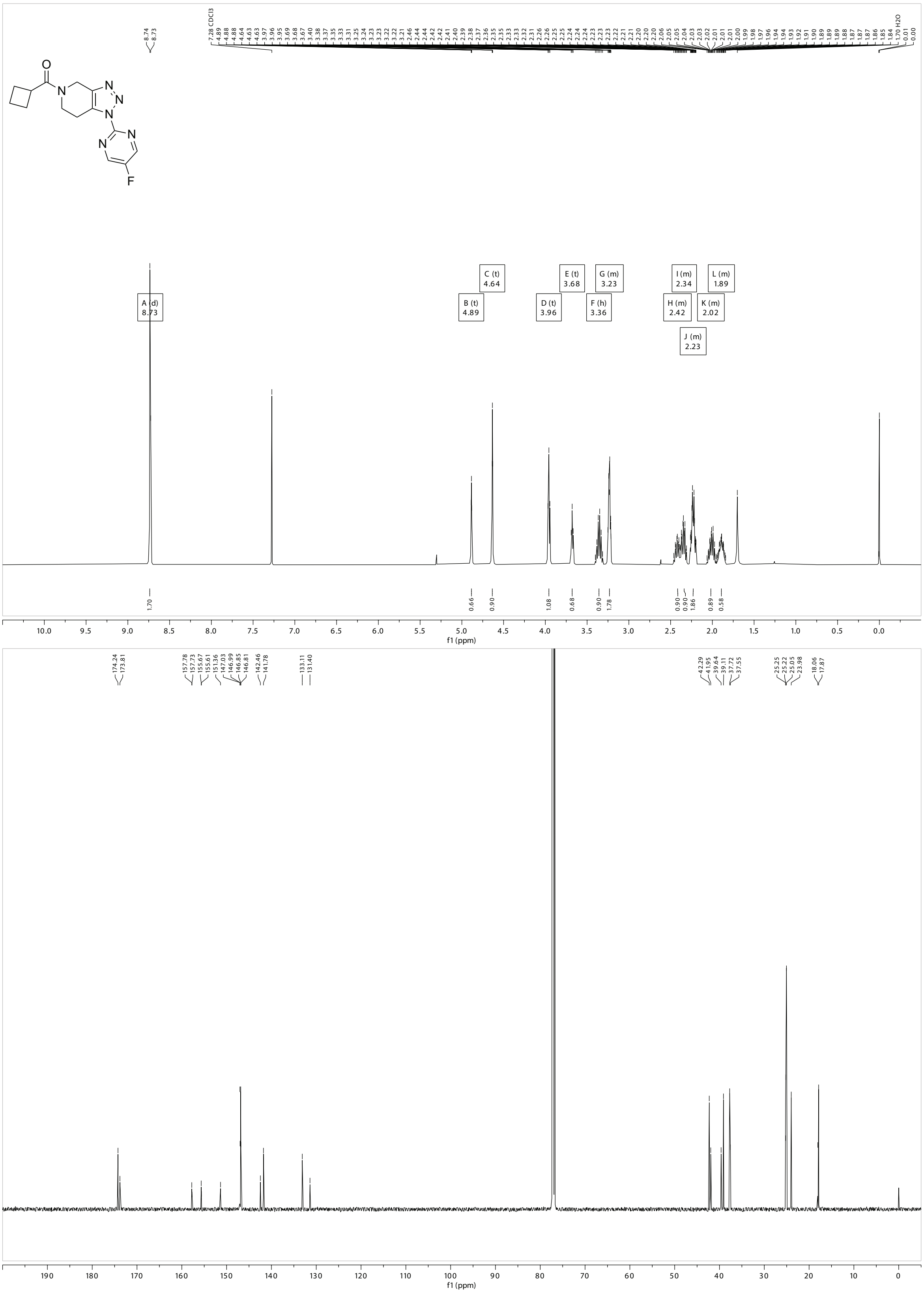

Figure 37: ^{1}H and ^{13}C of compound **1g**

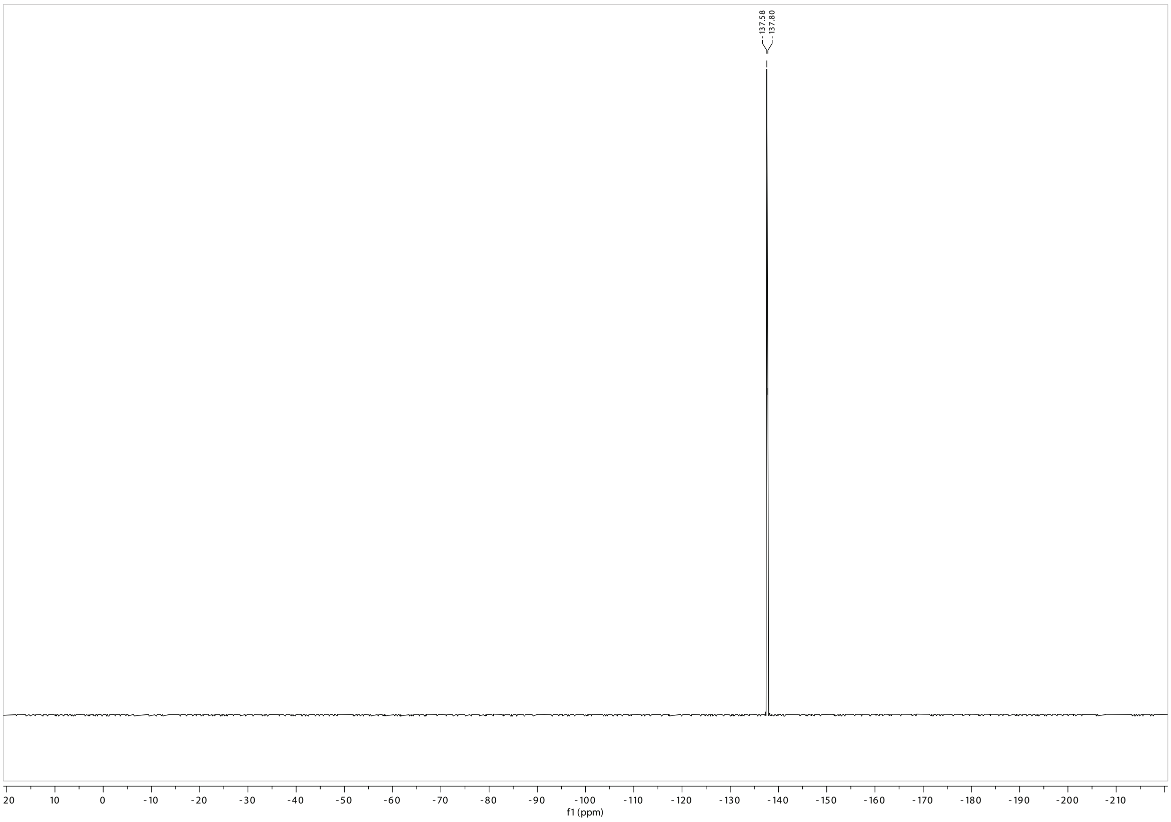

Figure 38: ^{19}F NMR of compound **1g**

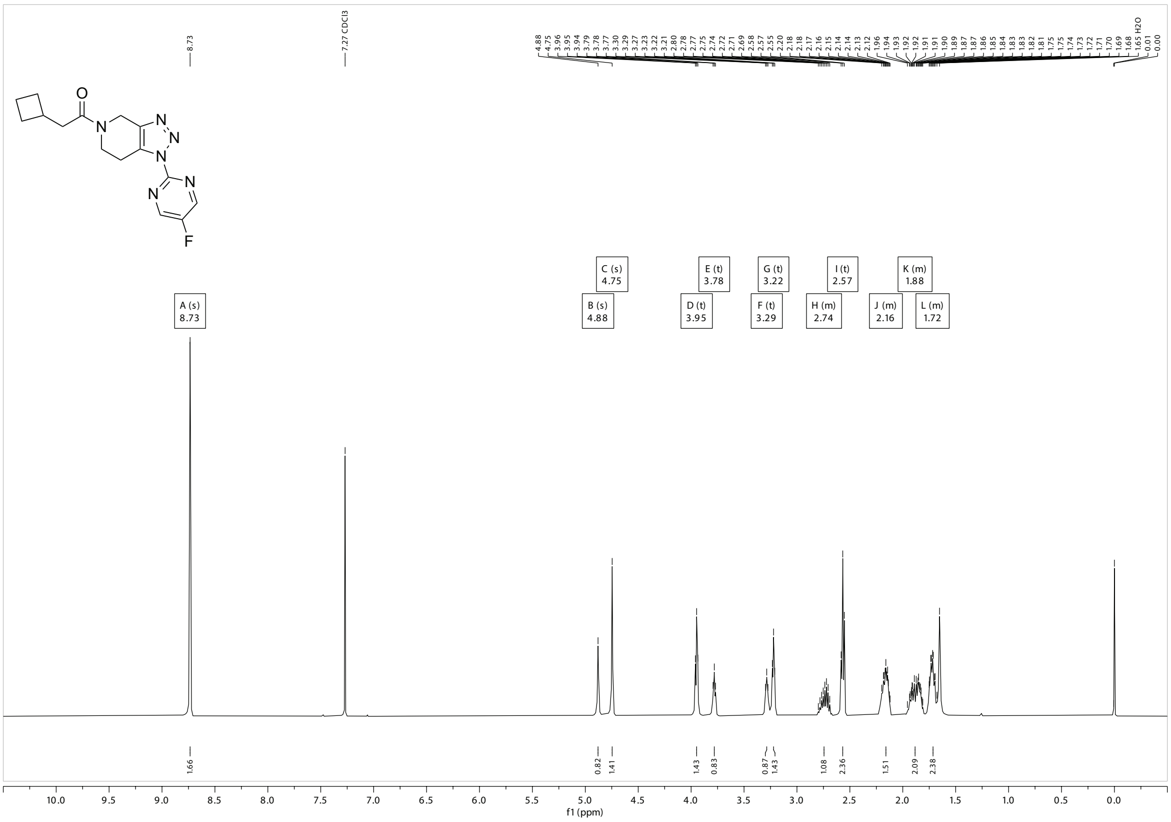

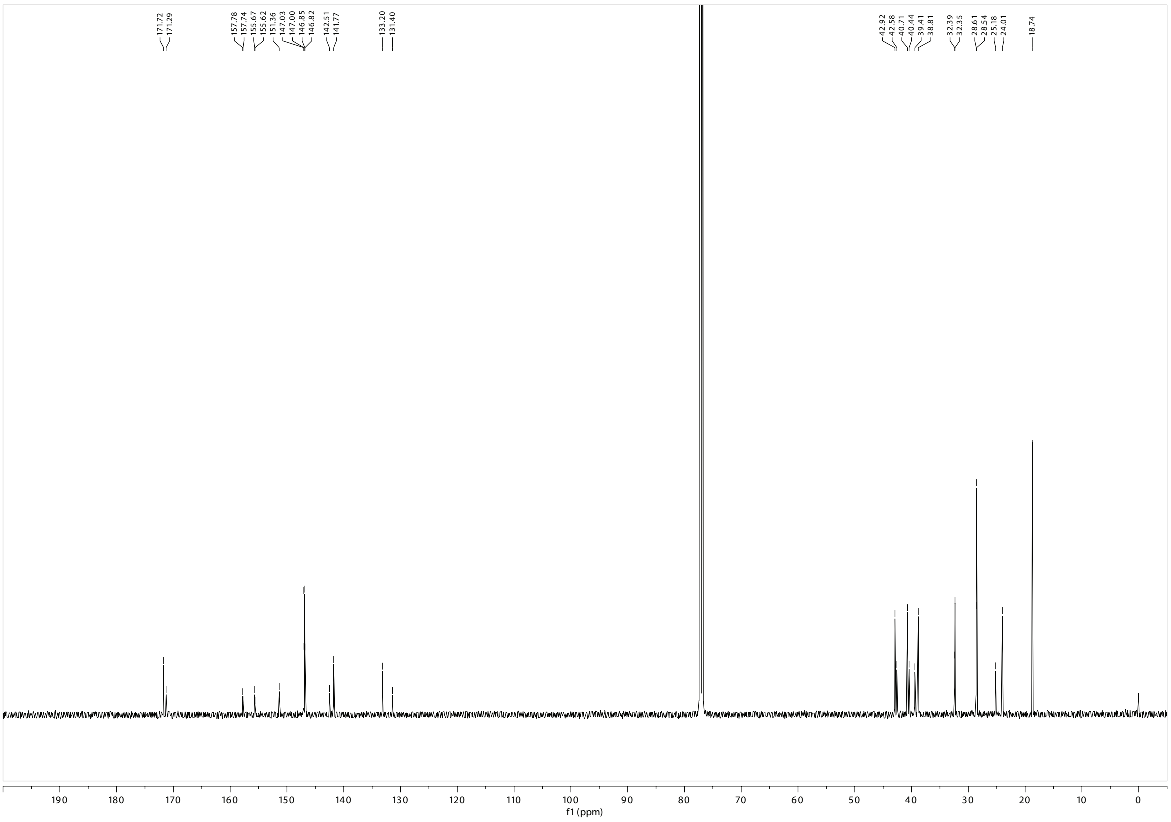

Figure 39: ^{1}H and ^{13}C of compound **1h**

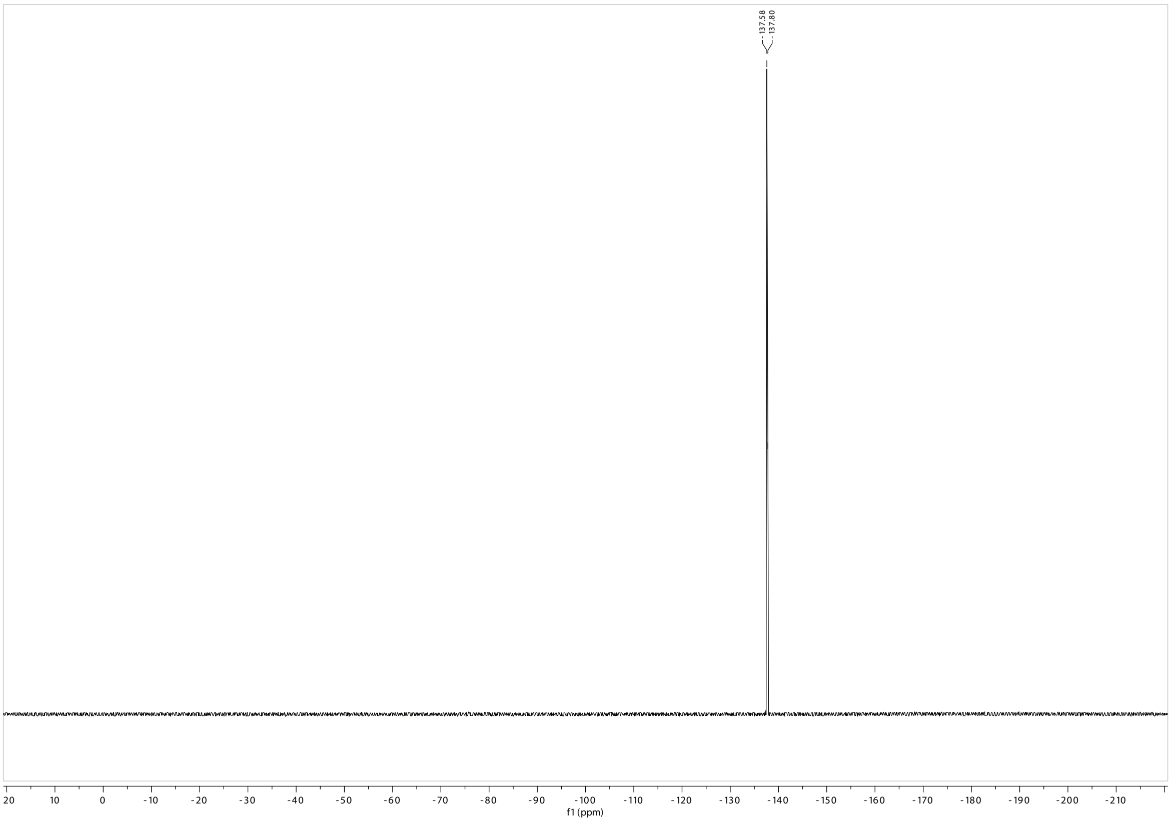

Figure 40: ^{19}F NMR of compound **1h**

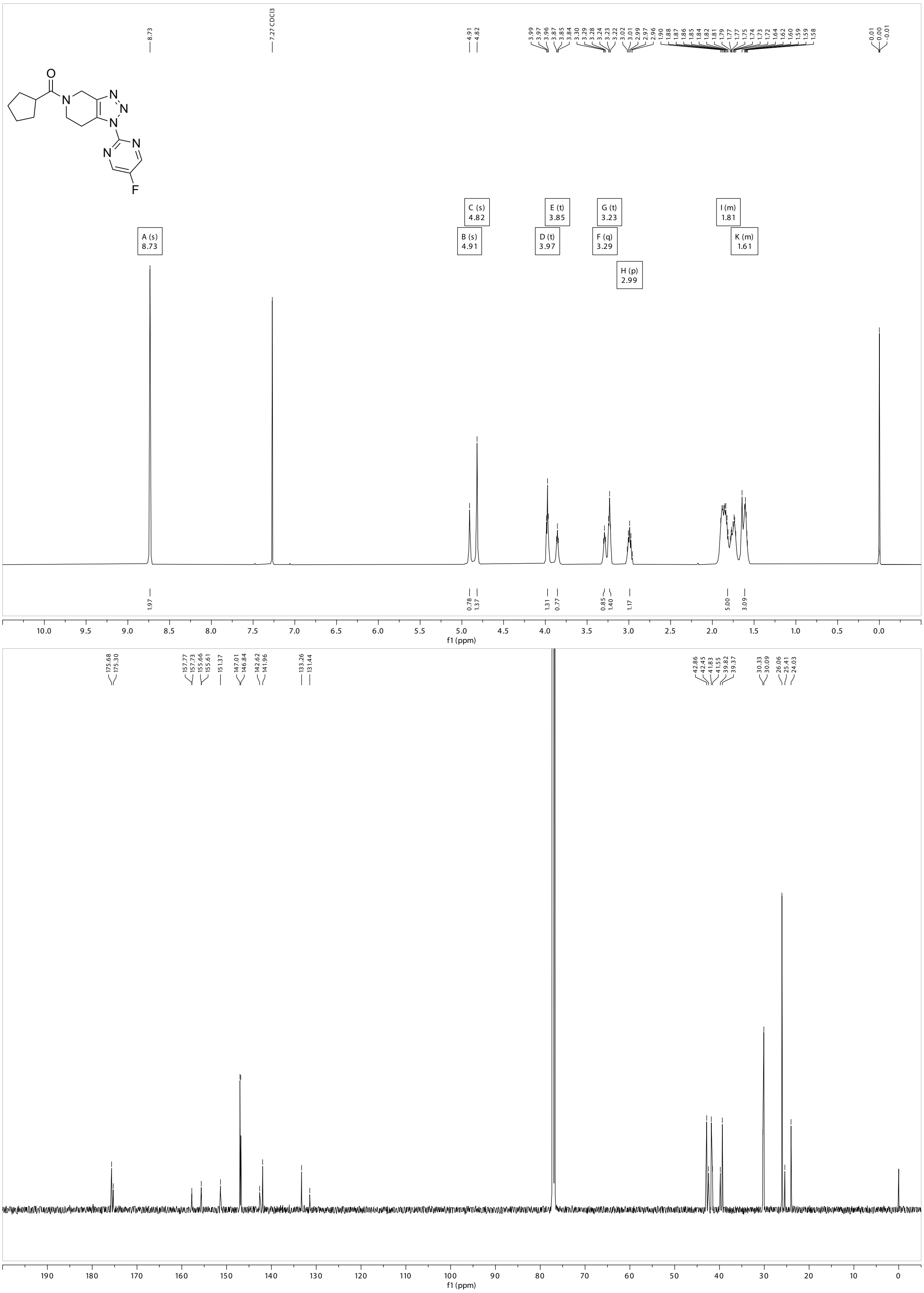

Figure 41: ^{1}H and ^{13}C of compound **1i**

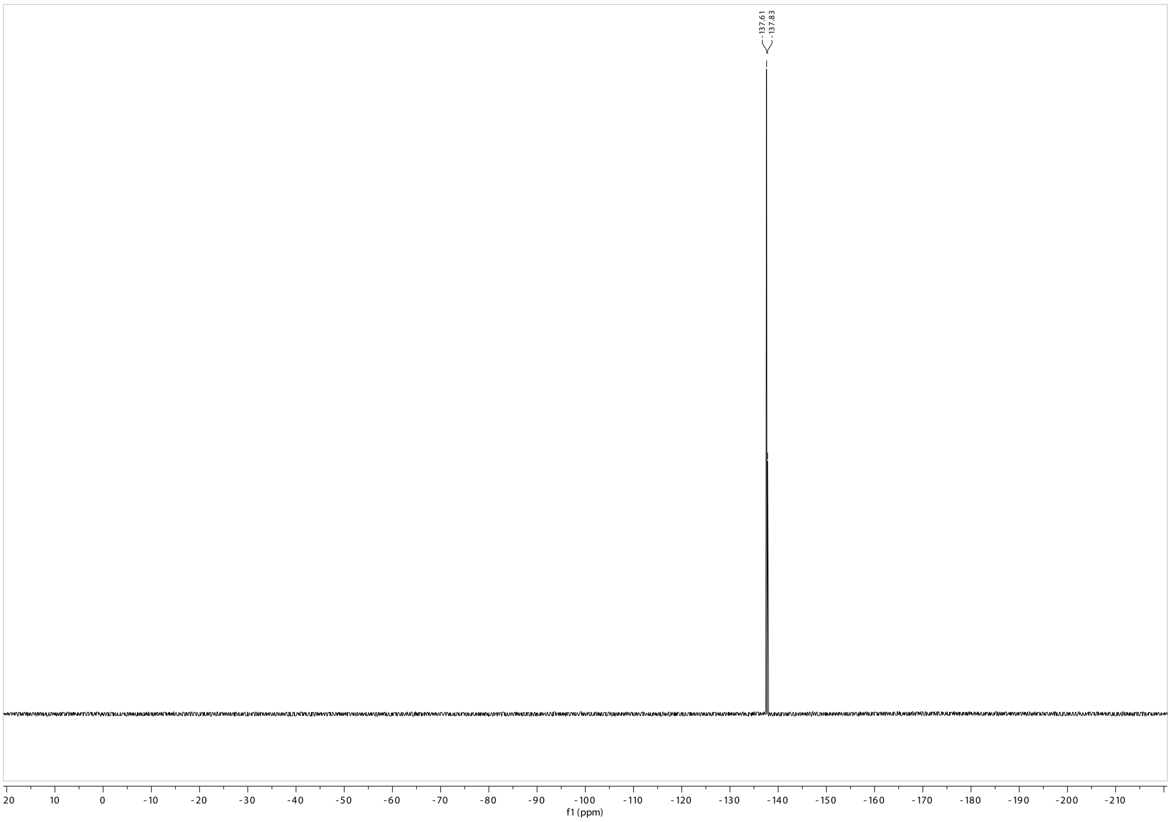

Figure 42: ^{19}F NMR of compound **1i**

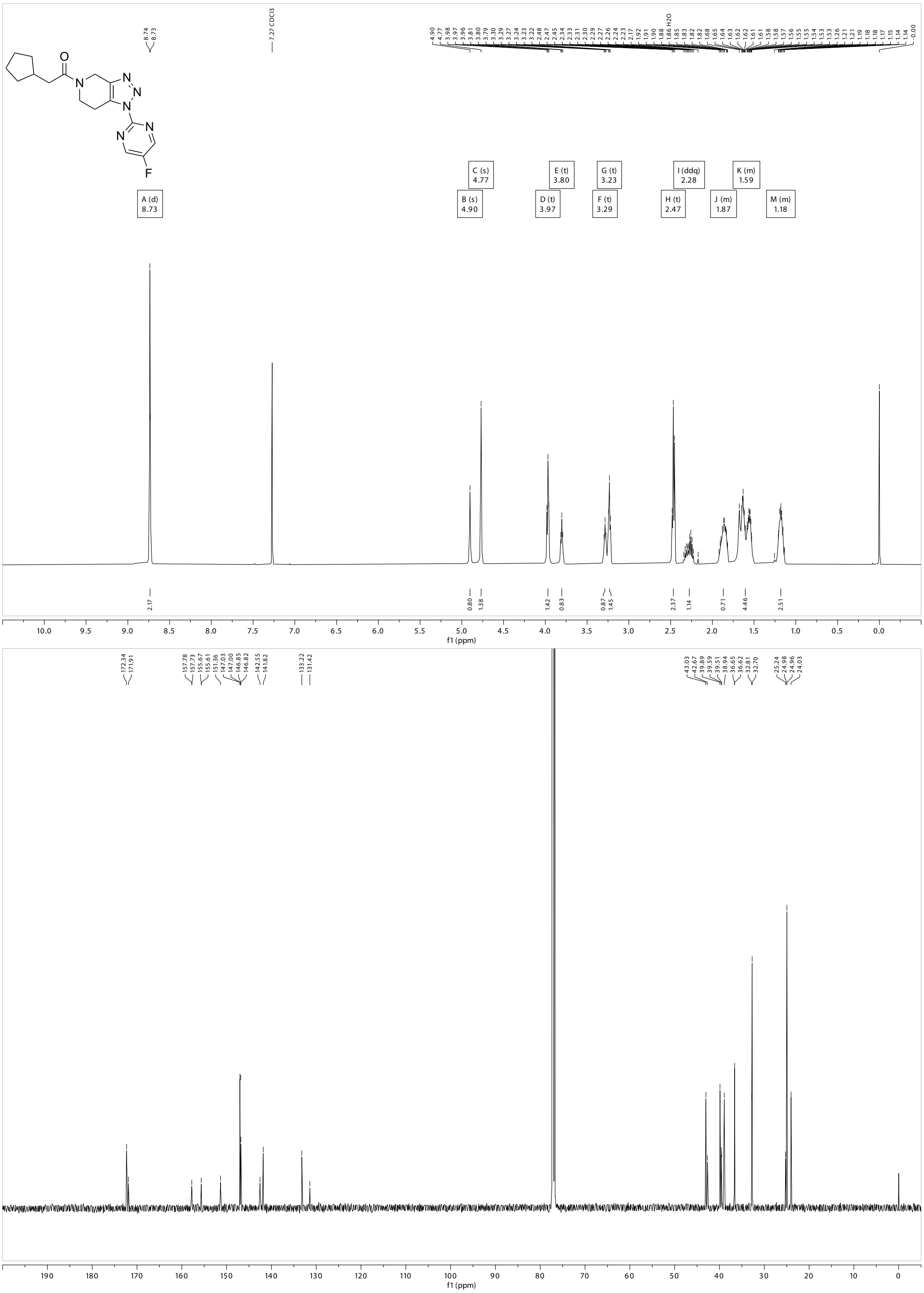

Figure 43: ^{1}H and ^{13}C of compound **1j**

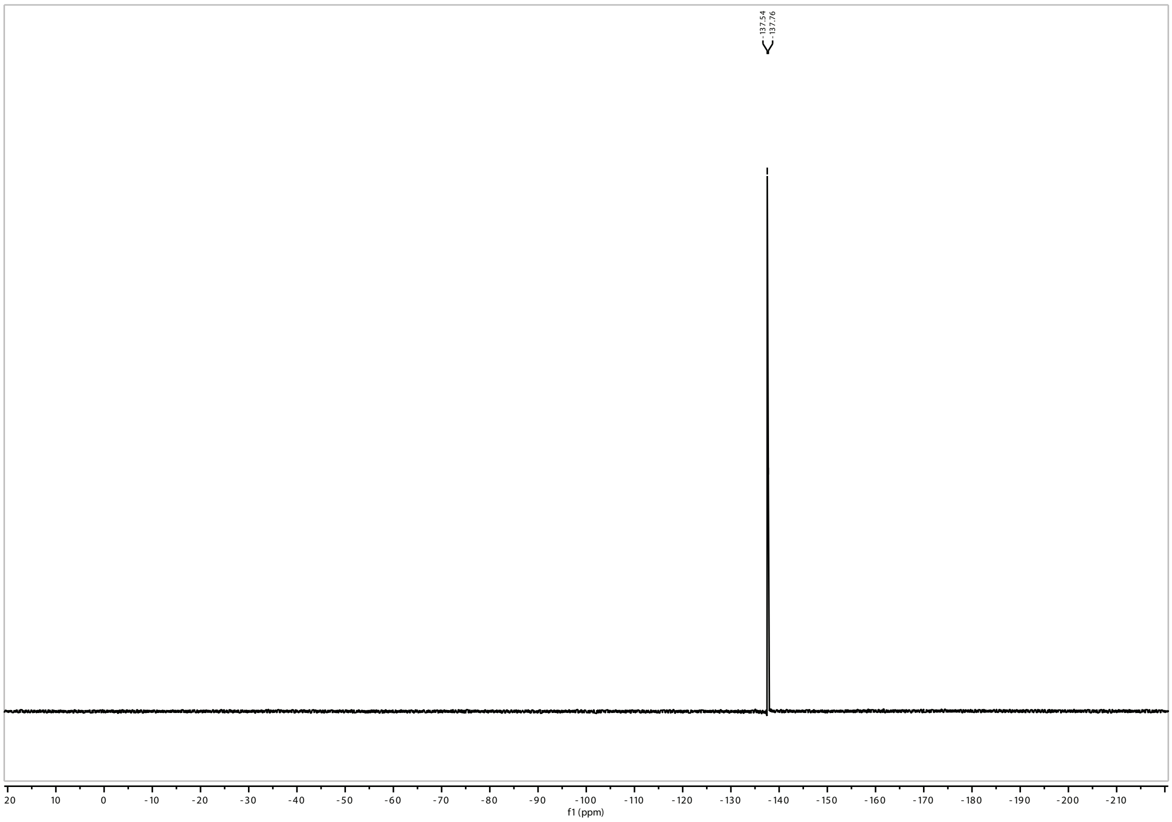

Figure 44: ^{19}F NMR of compound **1j**

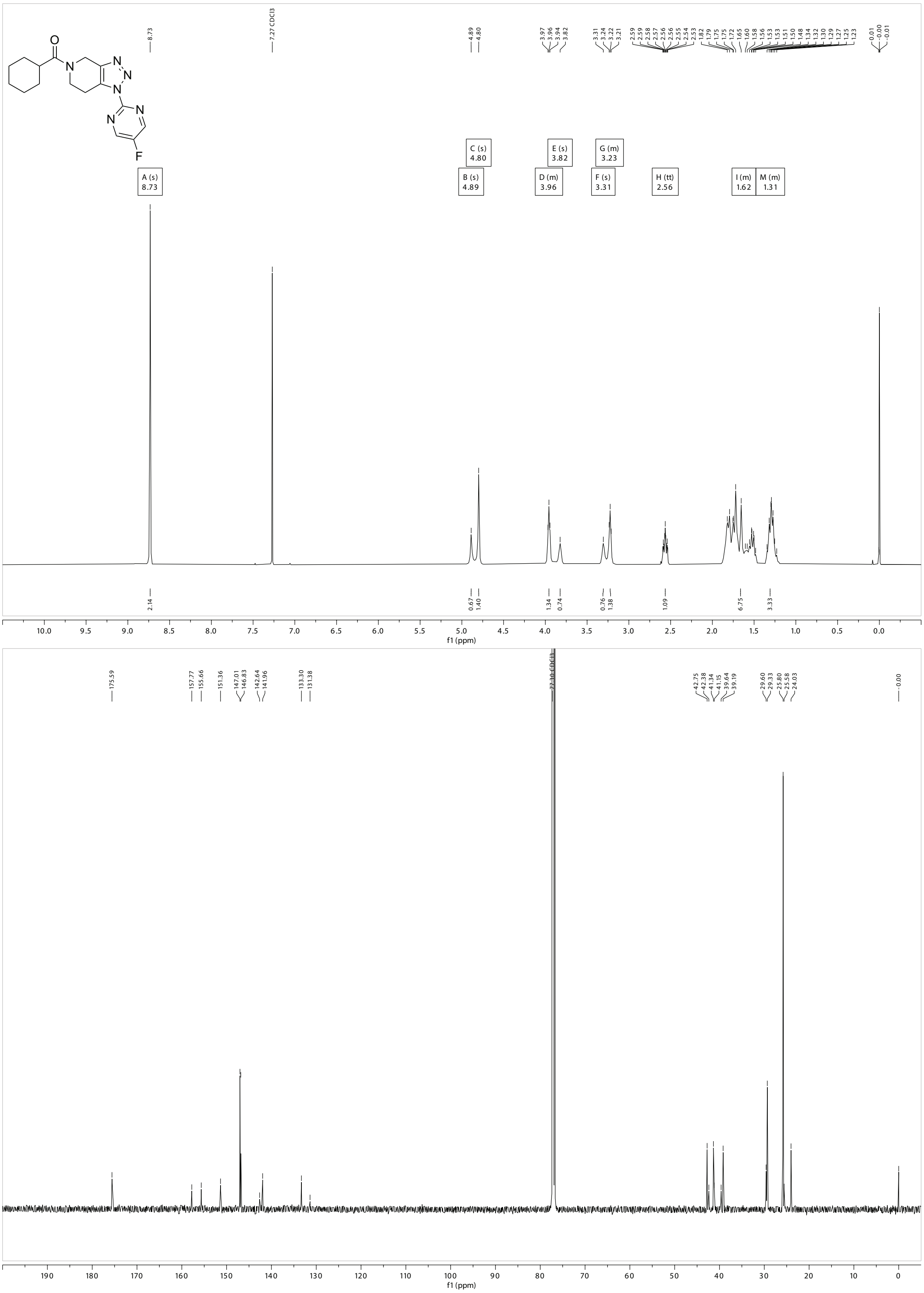

Figure 45: ^{1}H and ^{13}C of compound **1k**

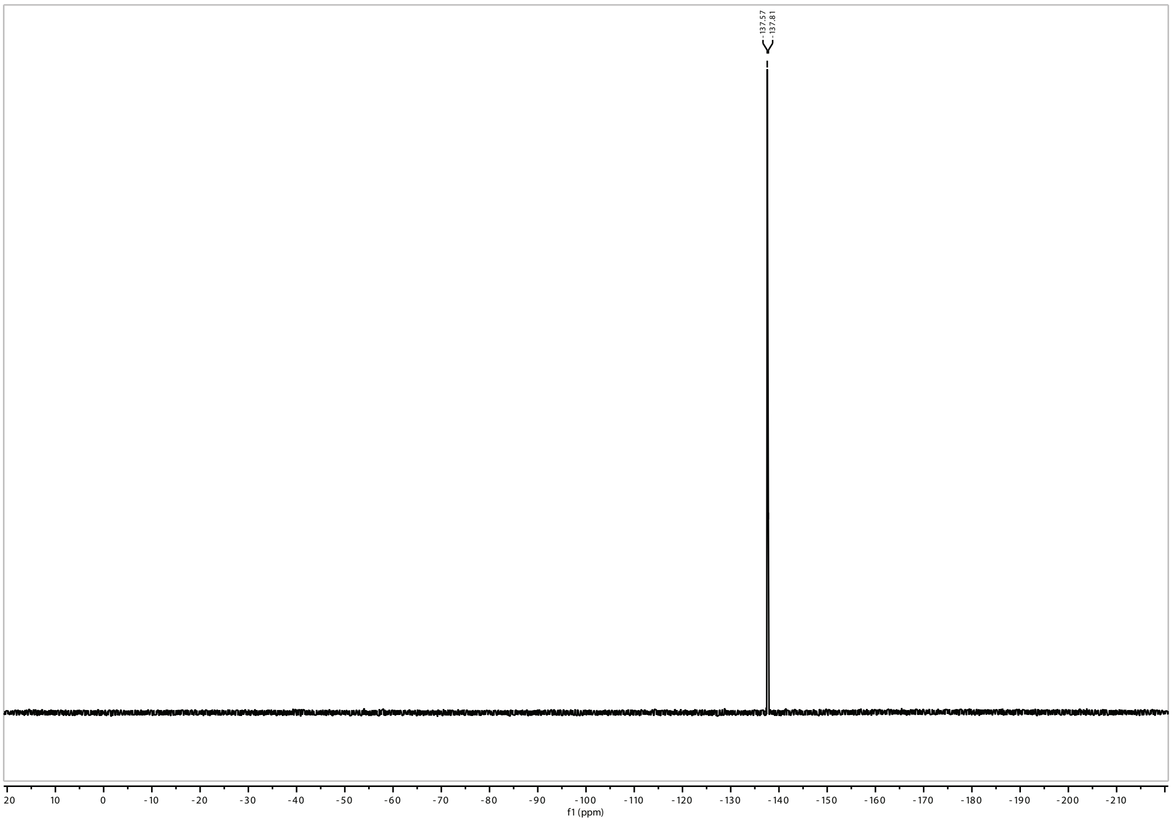

Figure 46: ^{19}F NMR of compound **1k**

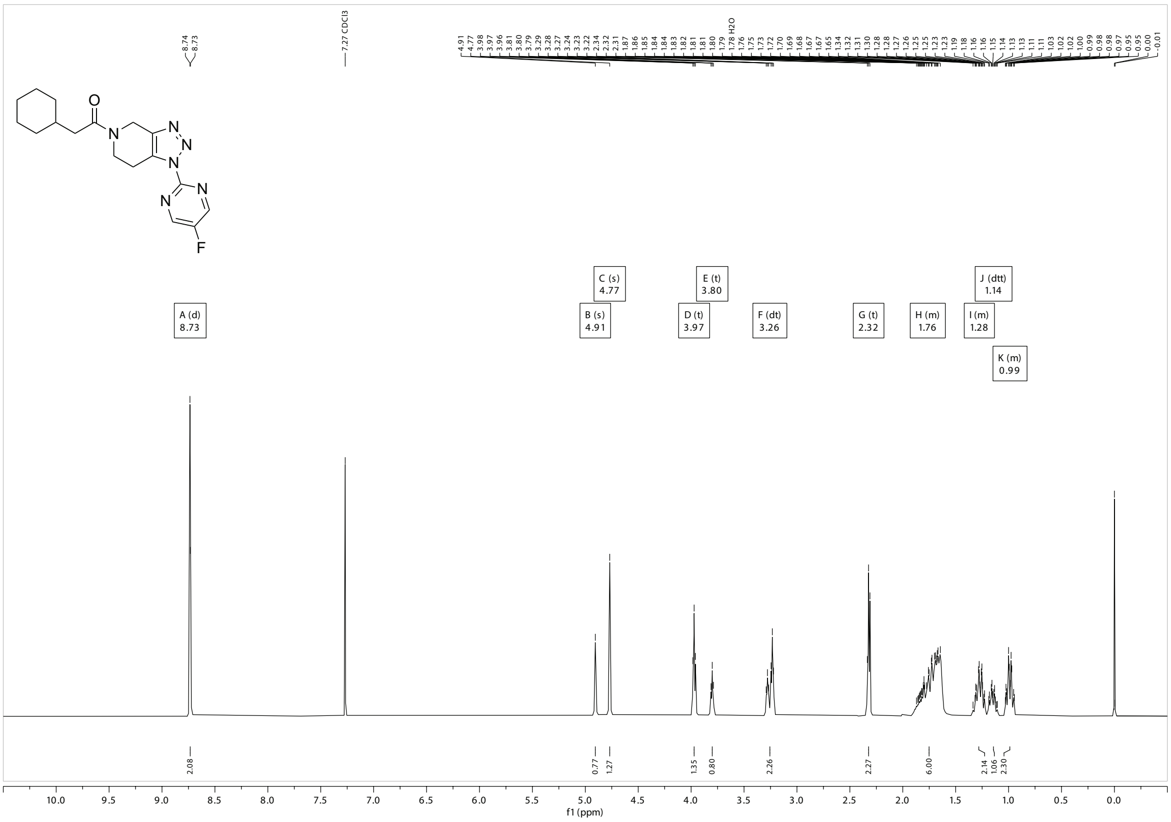

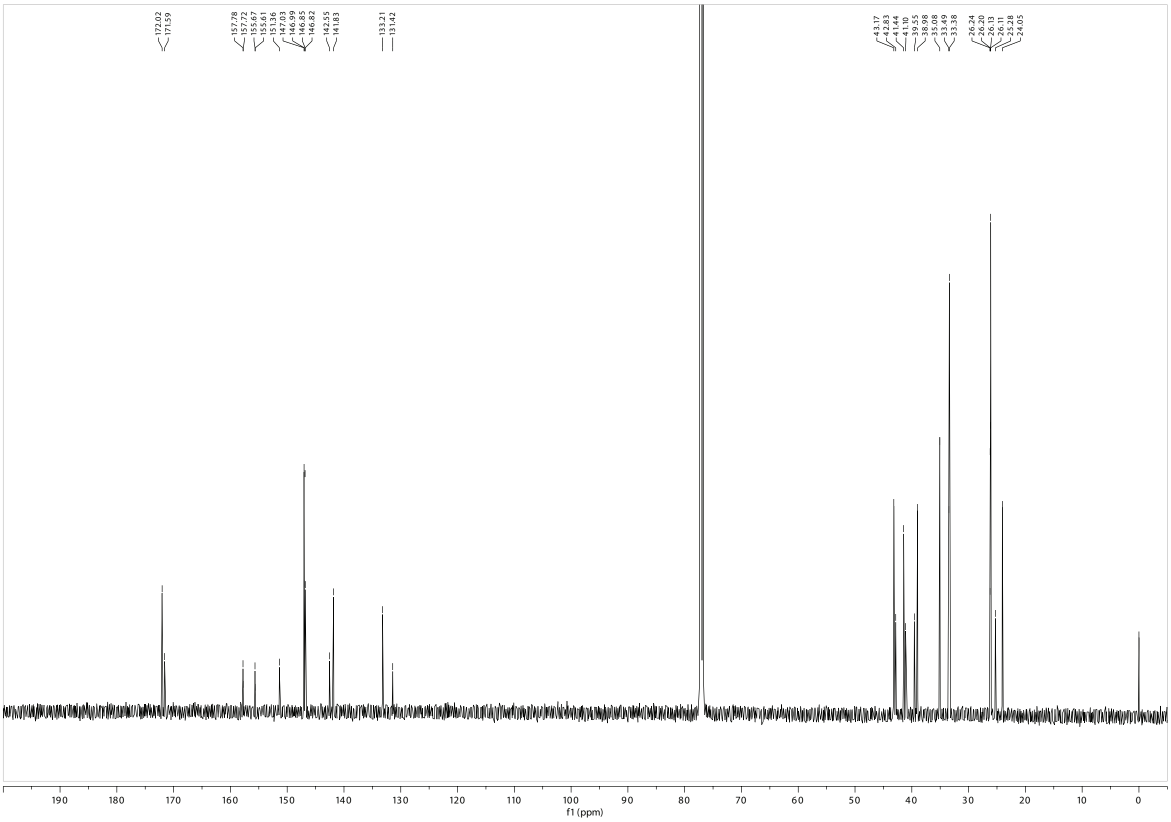

Figure 47: ^{1}H and ^{13}C of compound **1l**

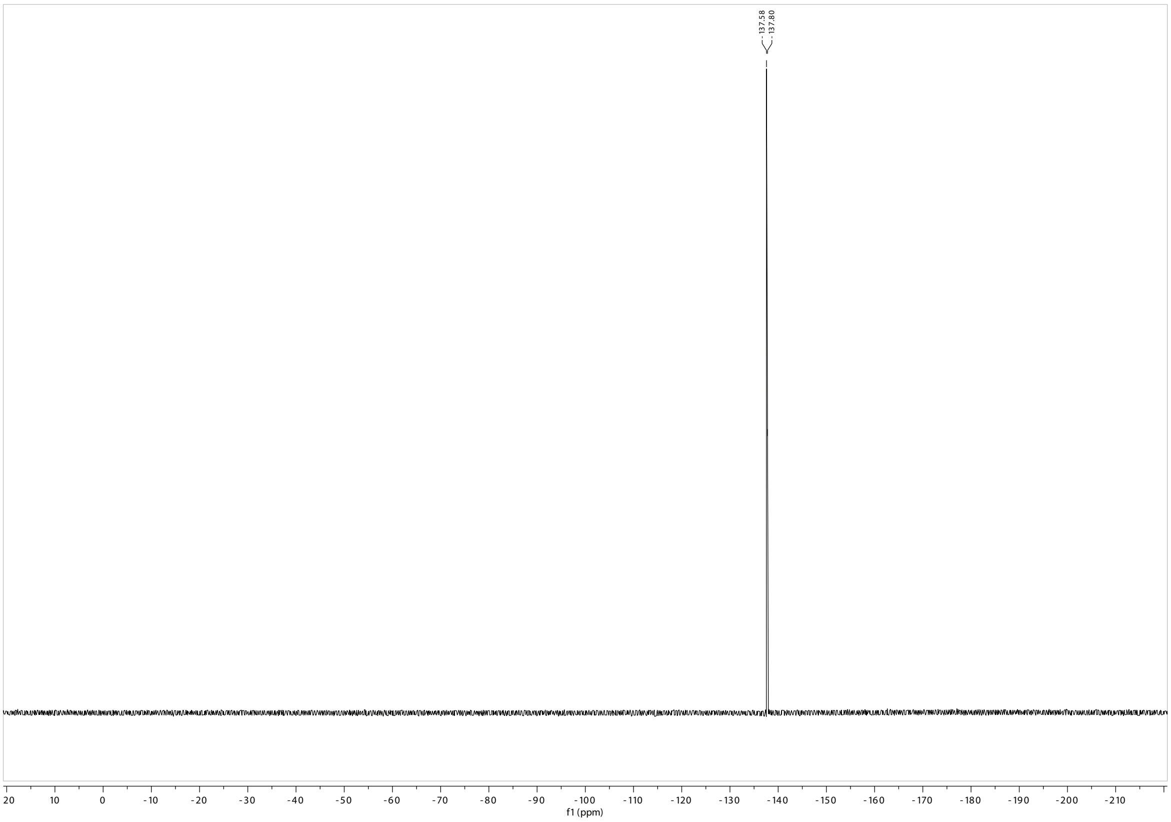

Figure 48: ^{19}F NMR of compound **1l**

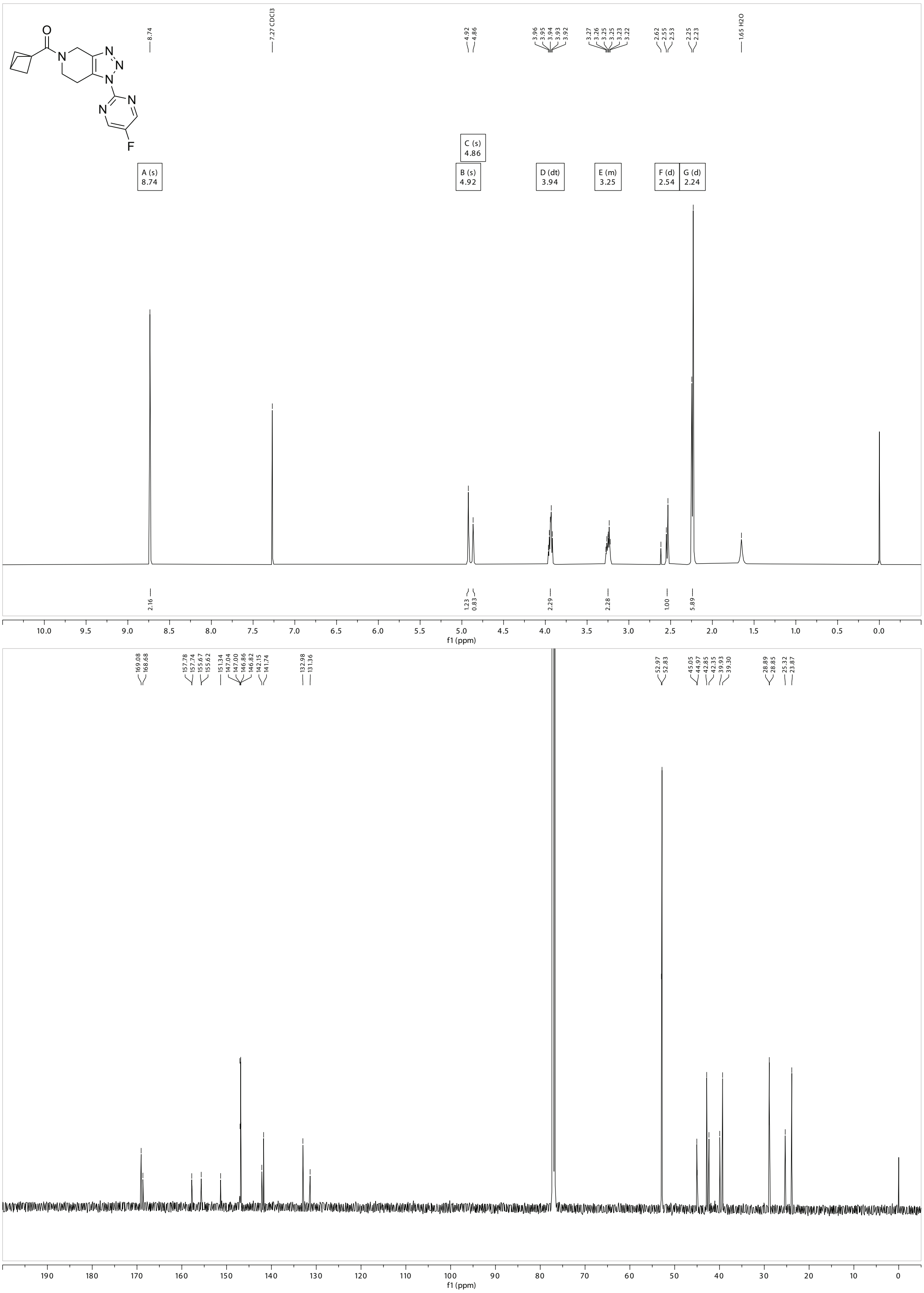

Figure 49: ^{1}H and ^{13}C of compound **1m**

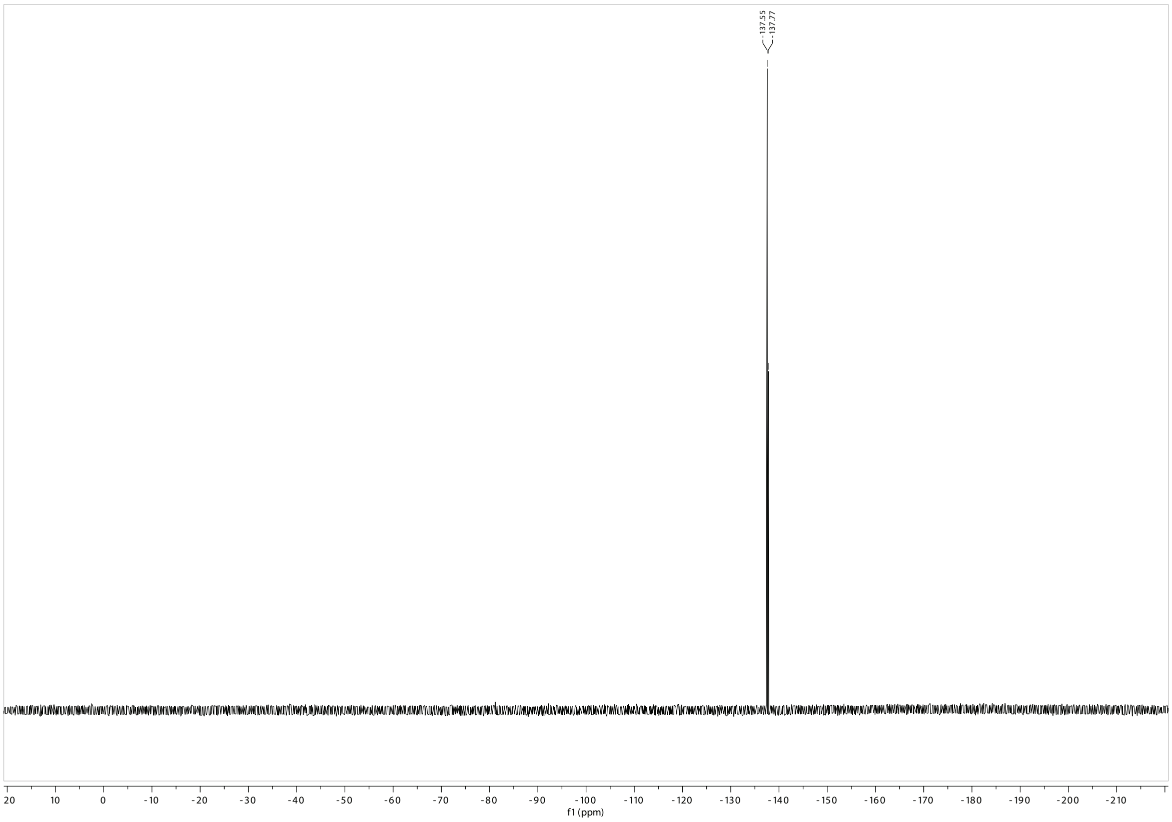

Figure 50: ^{19}F NMR of compound **1m**

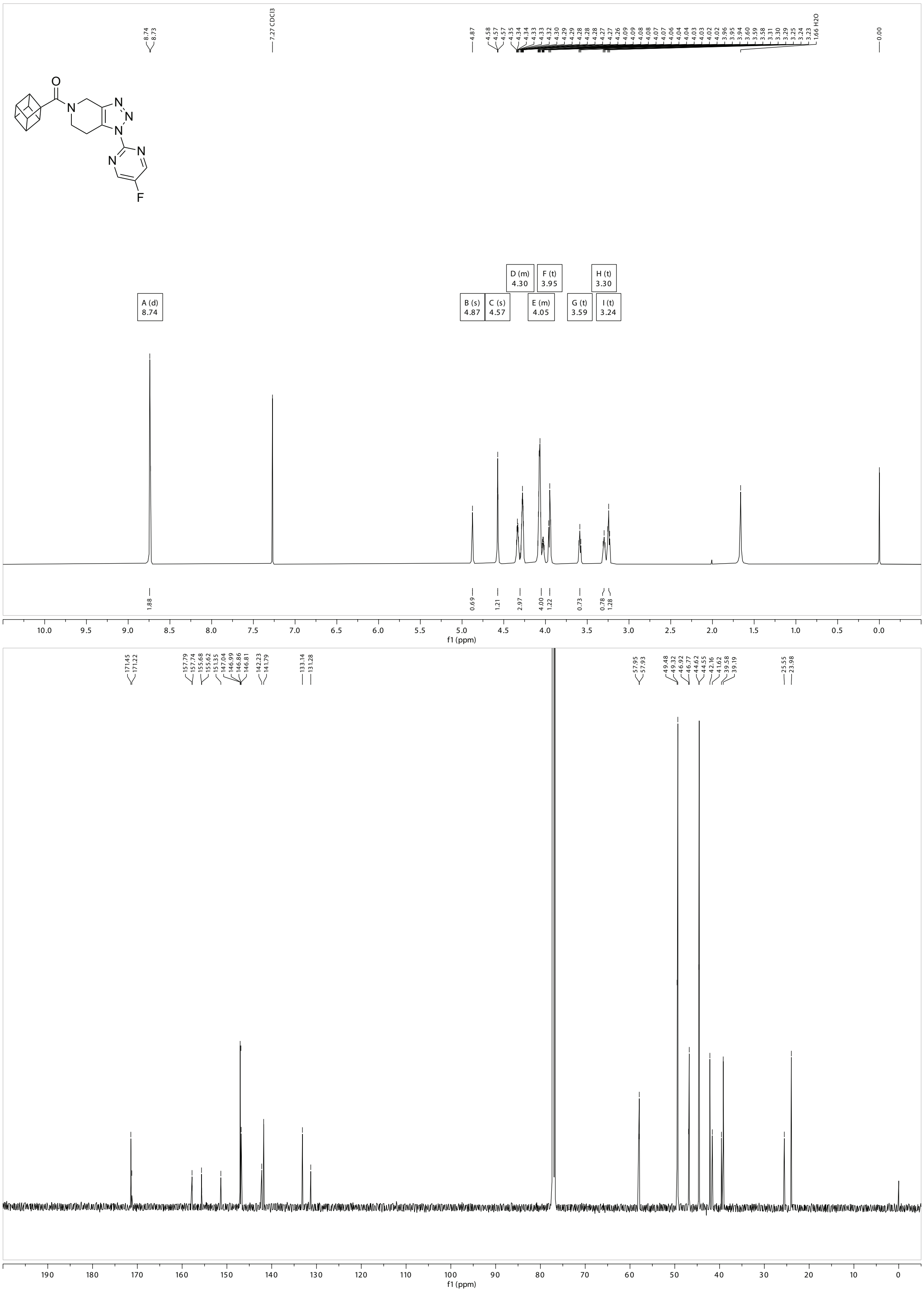

Figure 51: ^{1}H and ^{13}C of compound **1n**

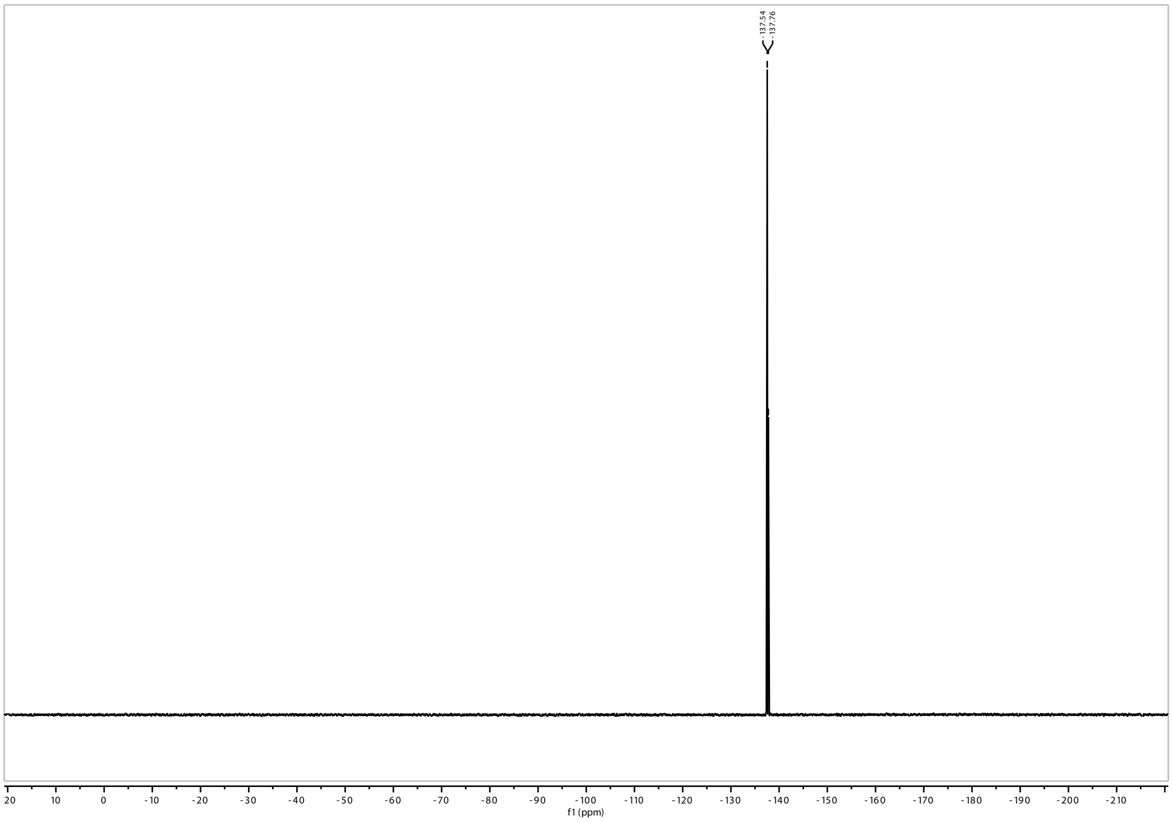

Figure 52: ^{19}F NMR of compound **1n**

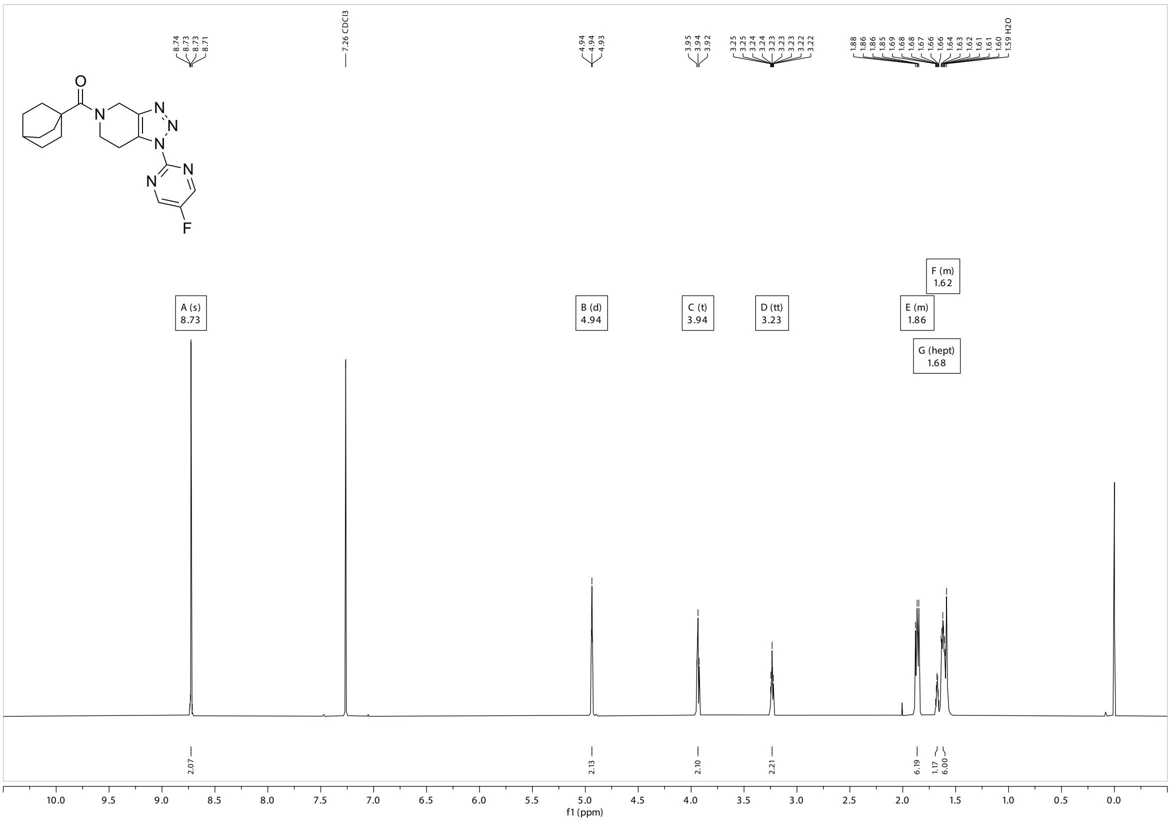

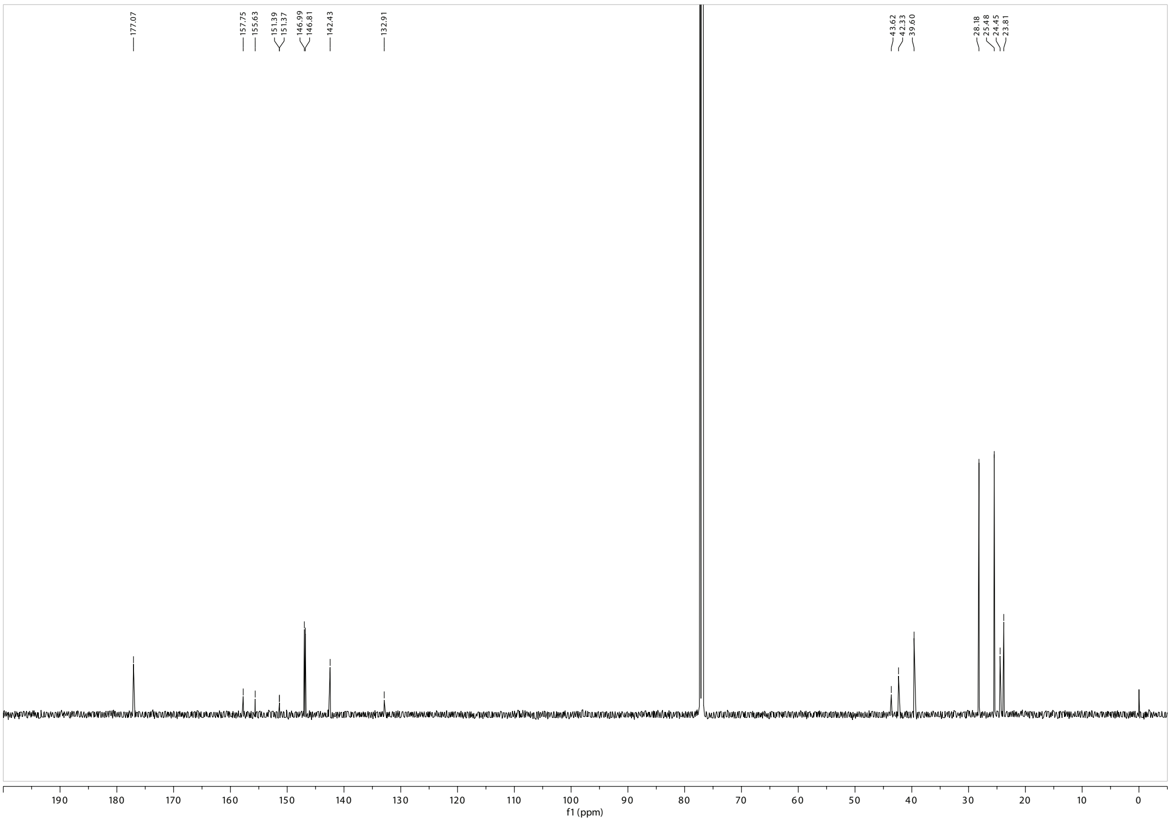

Figure 53: ^{1}H and ^{13}C of compound **1o**

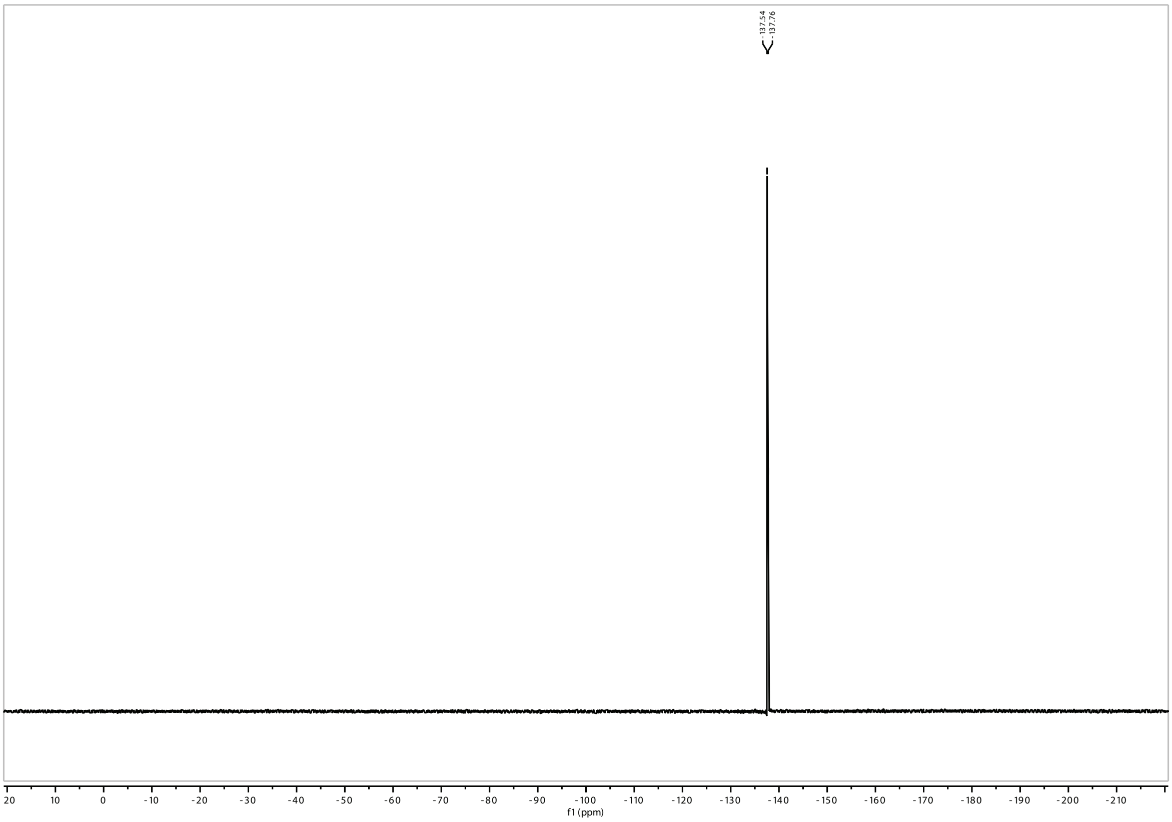

Figure 54: ^{19}F NMR of compound **1o**

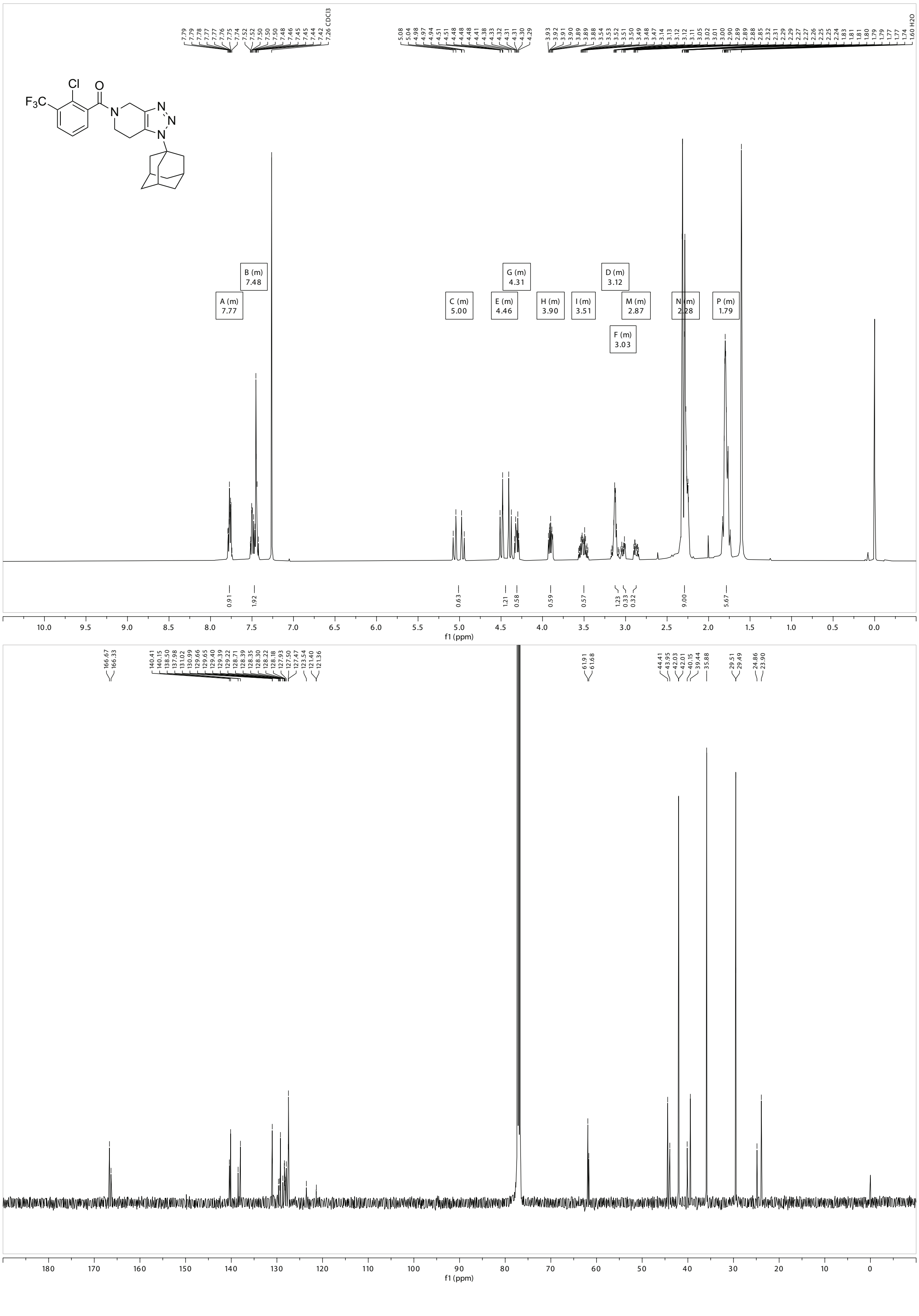

Figure 55: ^{1}H and ^{13}C of compound **2a**

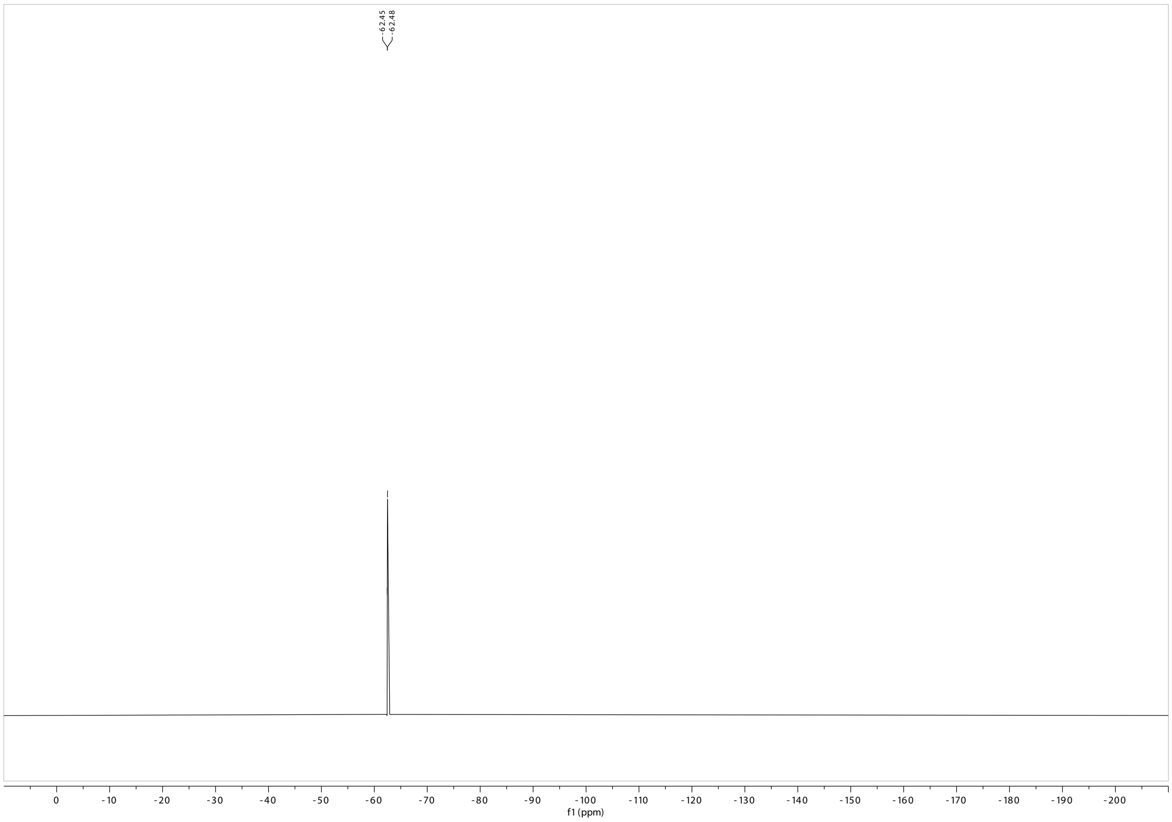

Figure 56: ^{19}F NMR of compound **2a**

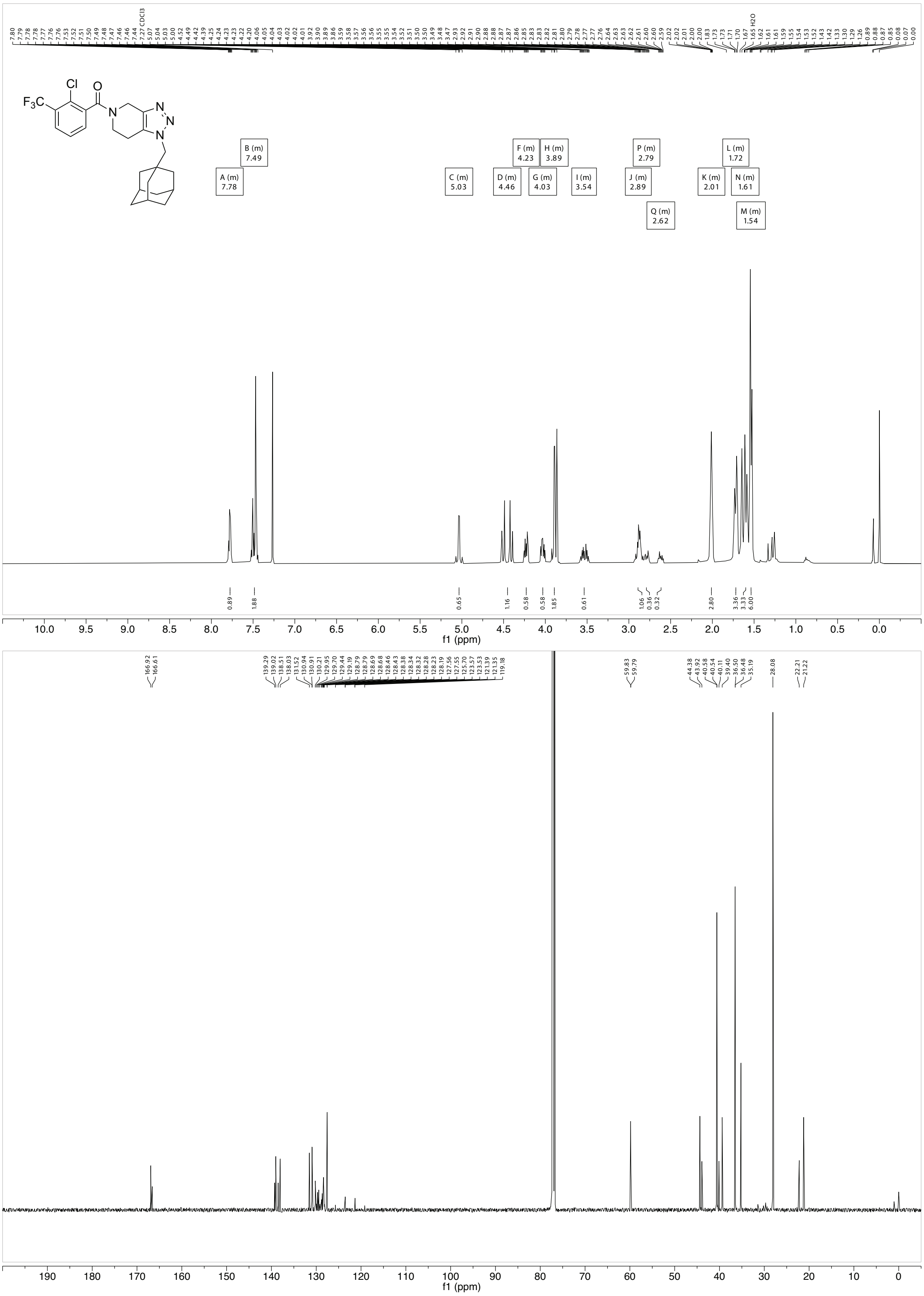

Figure 57: ^{1}H and ^{13}C of compound **2b**

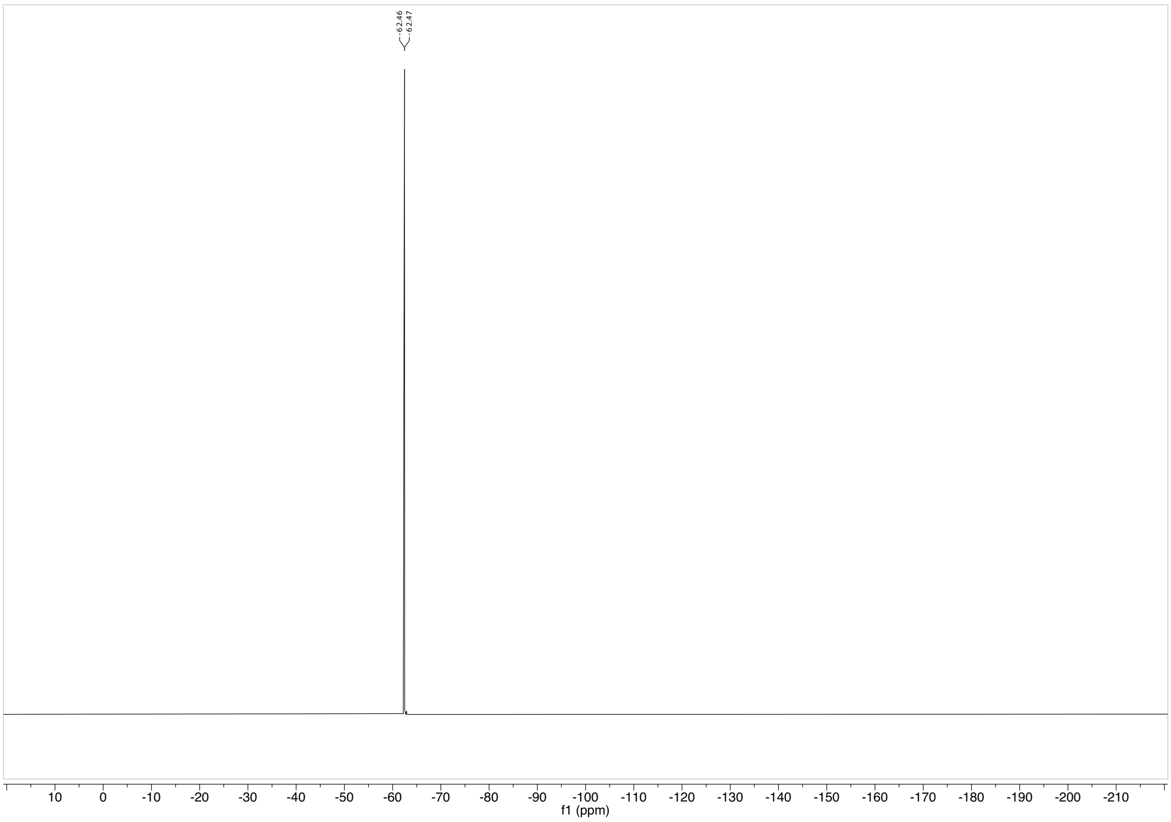

Figure 58: ^{19}F NMR of compound **2b**

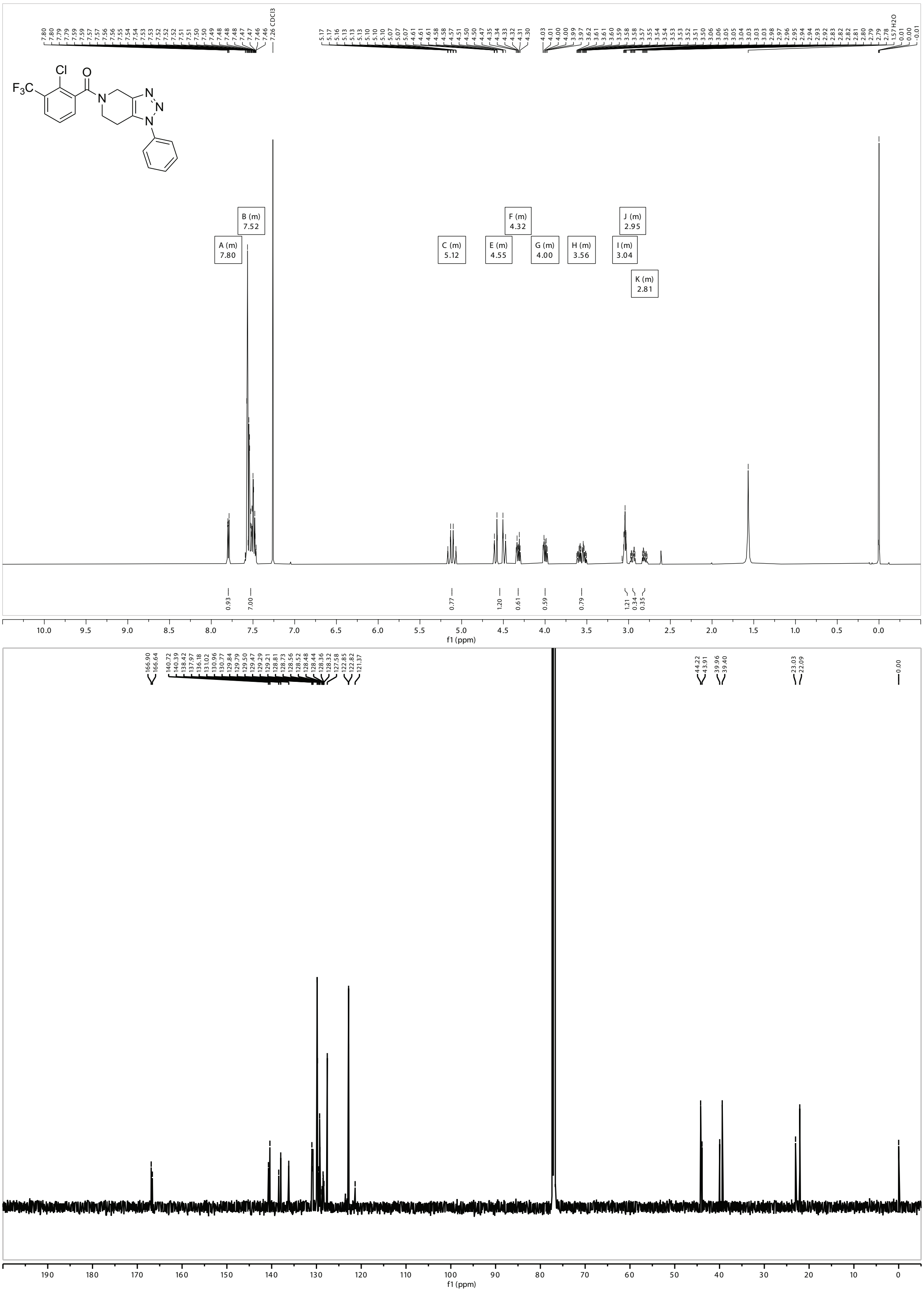

Figure 59: ^{1}H and ^{13}C of compound **2c**

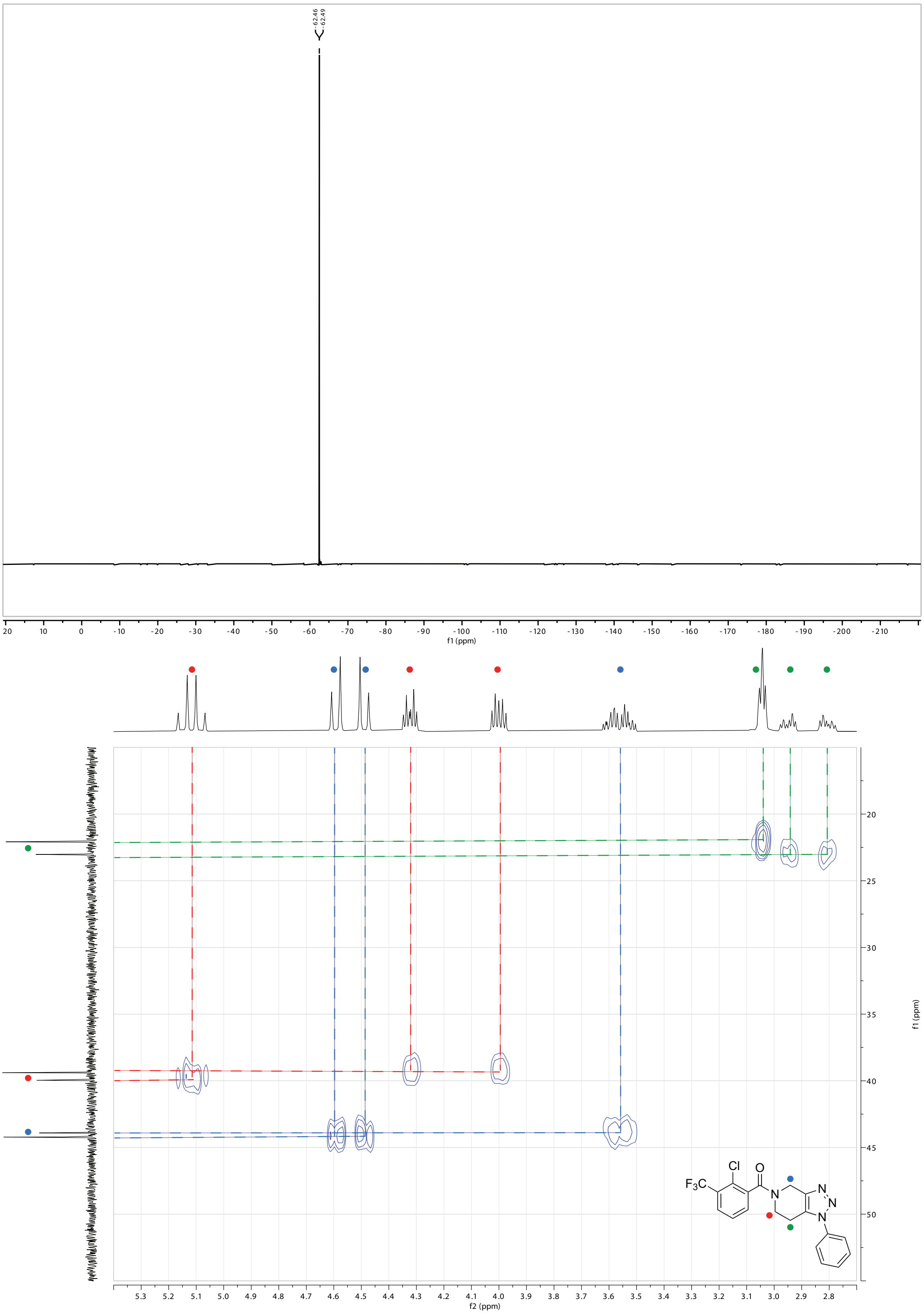

Figure 60: ^{19}F NMR and ^{1}H -^{13}C HSQC of compound **2c**

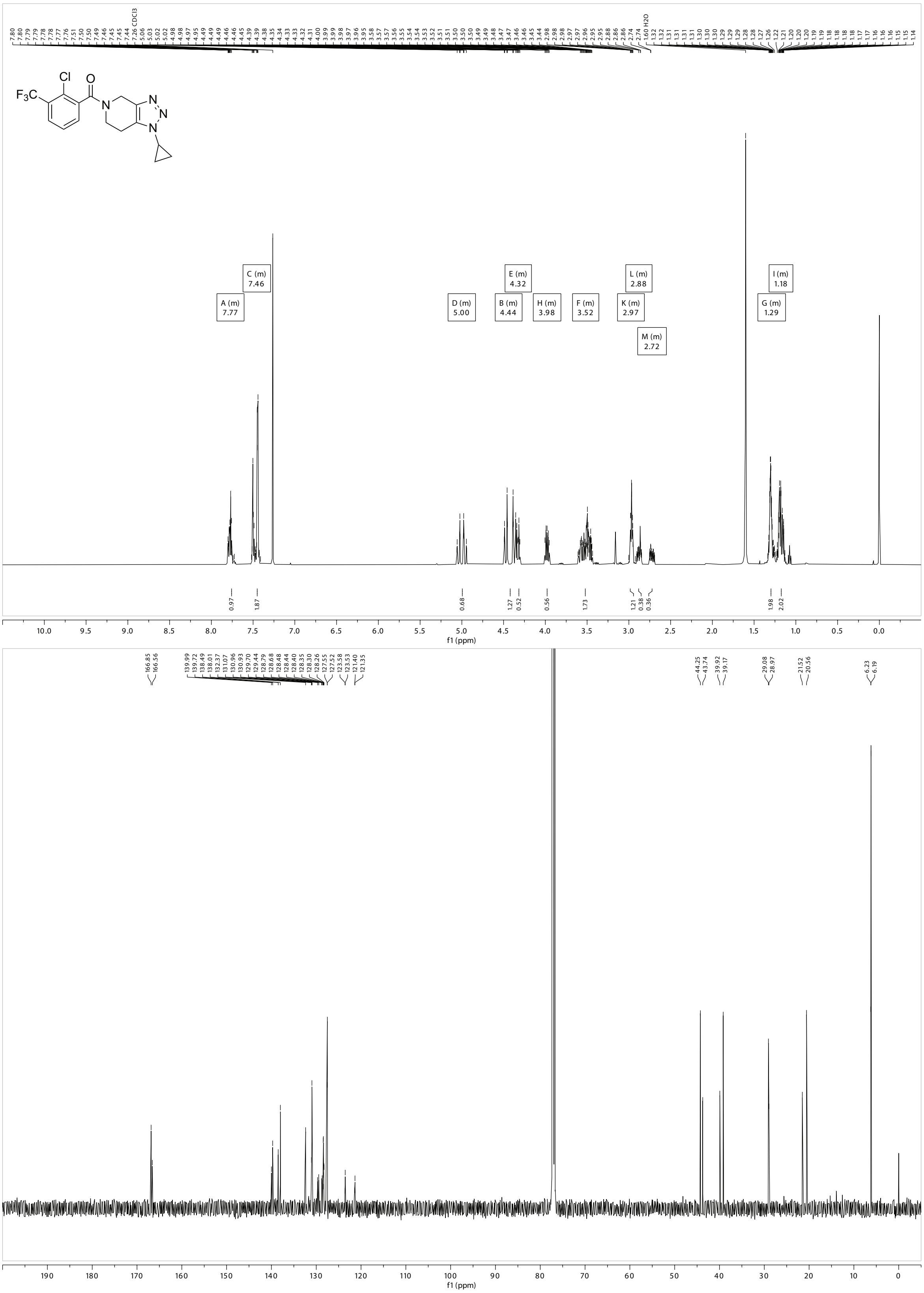

Figure 61: ^{1}H and ^{13}C of compound **2d**

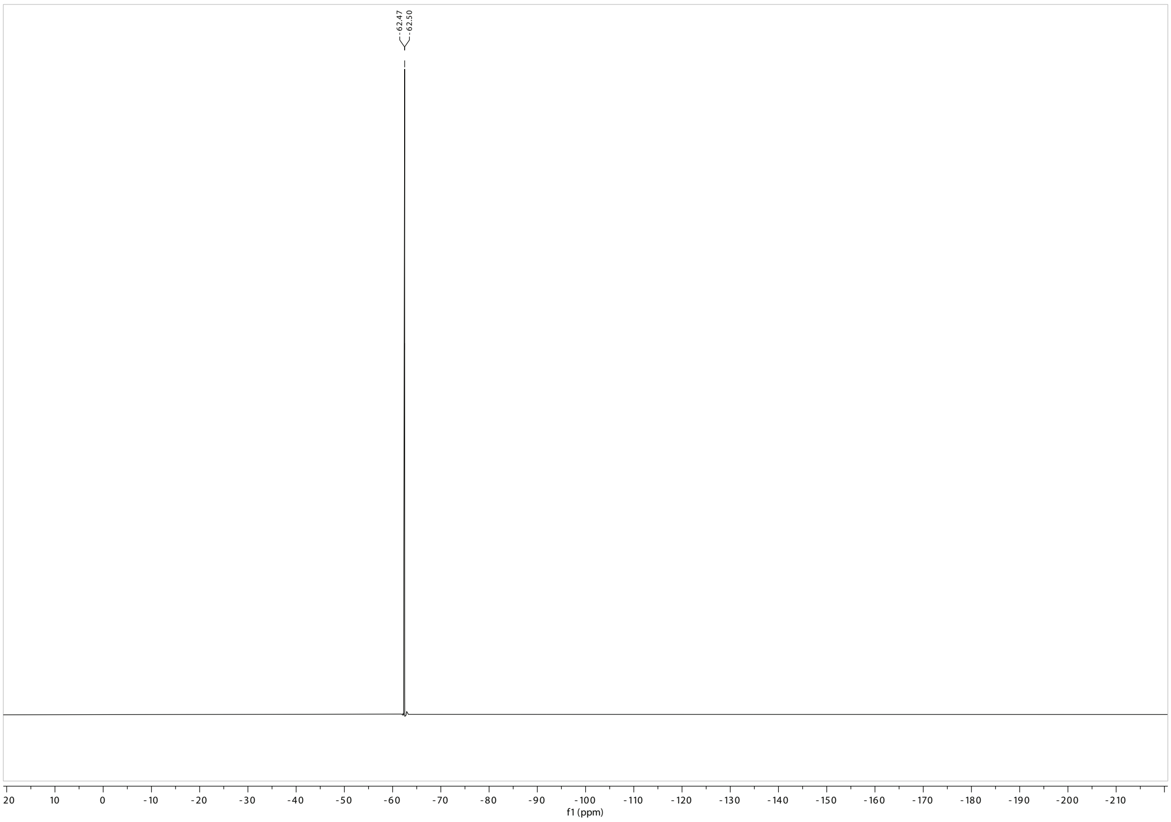

Figure 62: ^{19}F NMR of compound **2d**

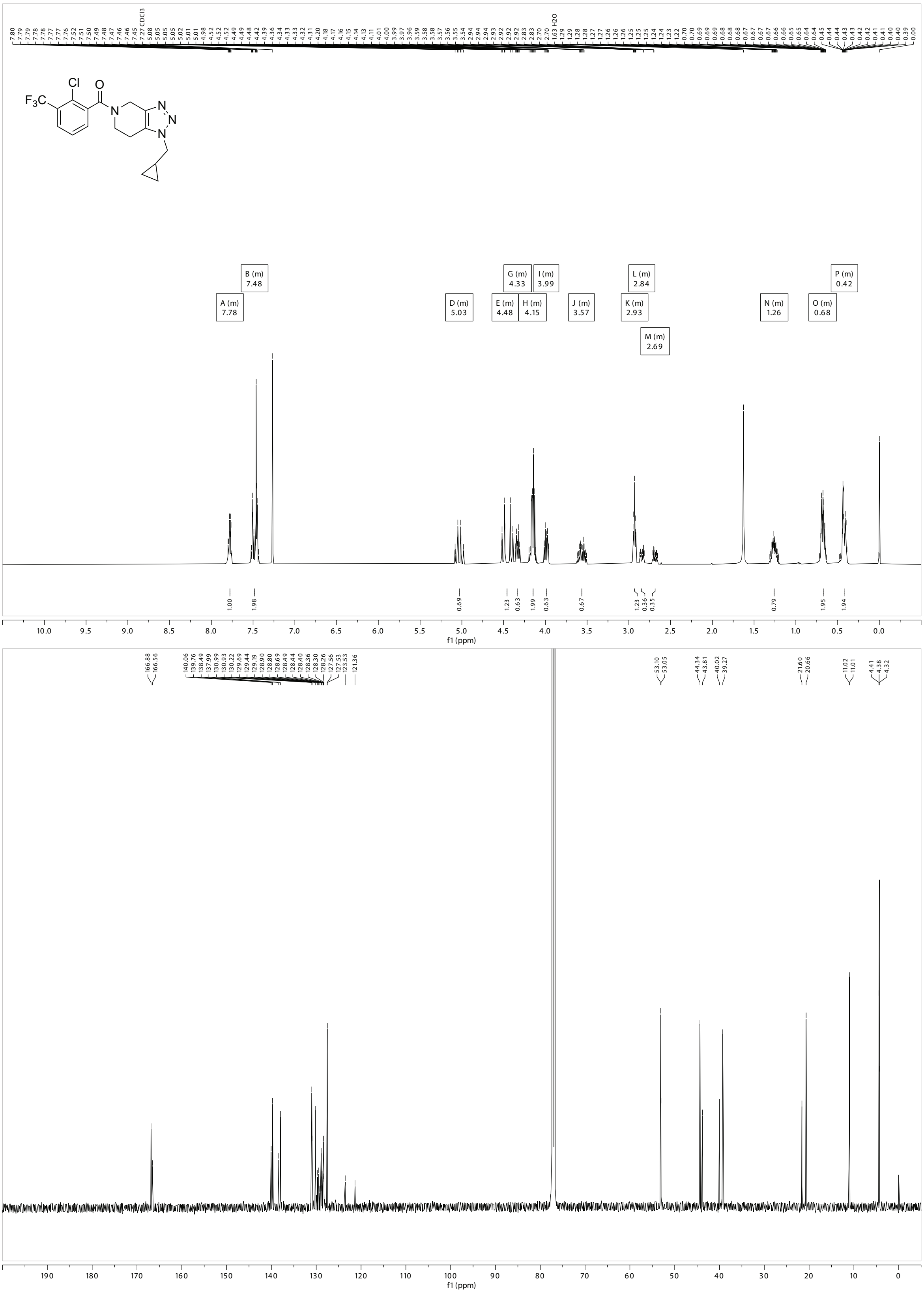

Figure 63: ^{1}H and ^{13}C of compound **2e**

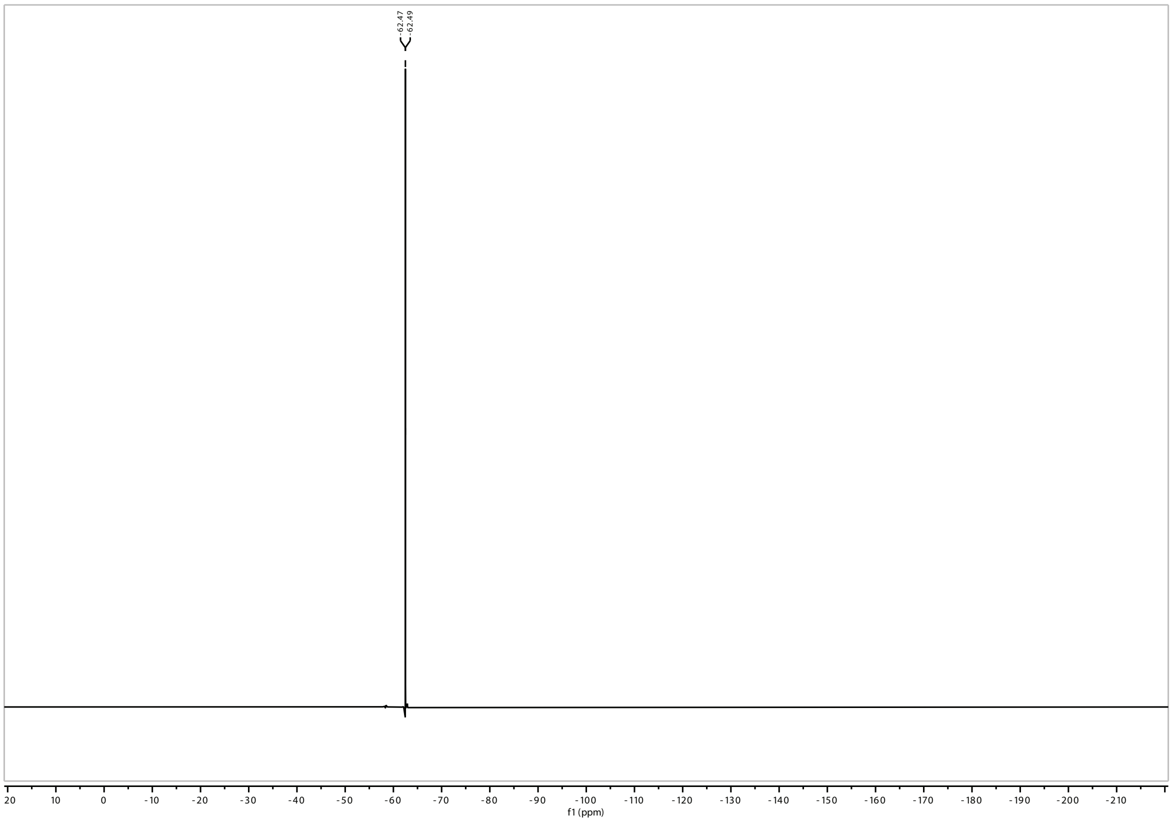

Figure 64: ^{19}F NMR of compound **2e**

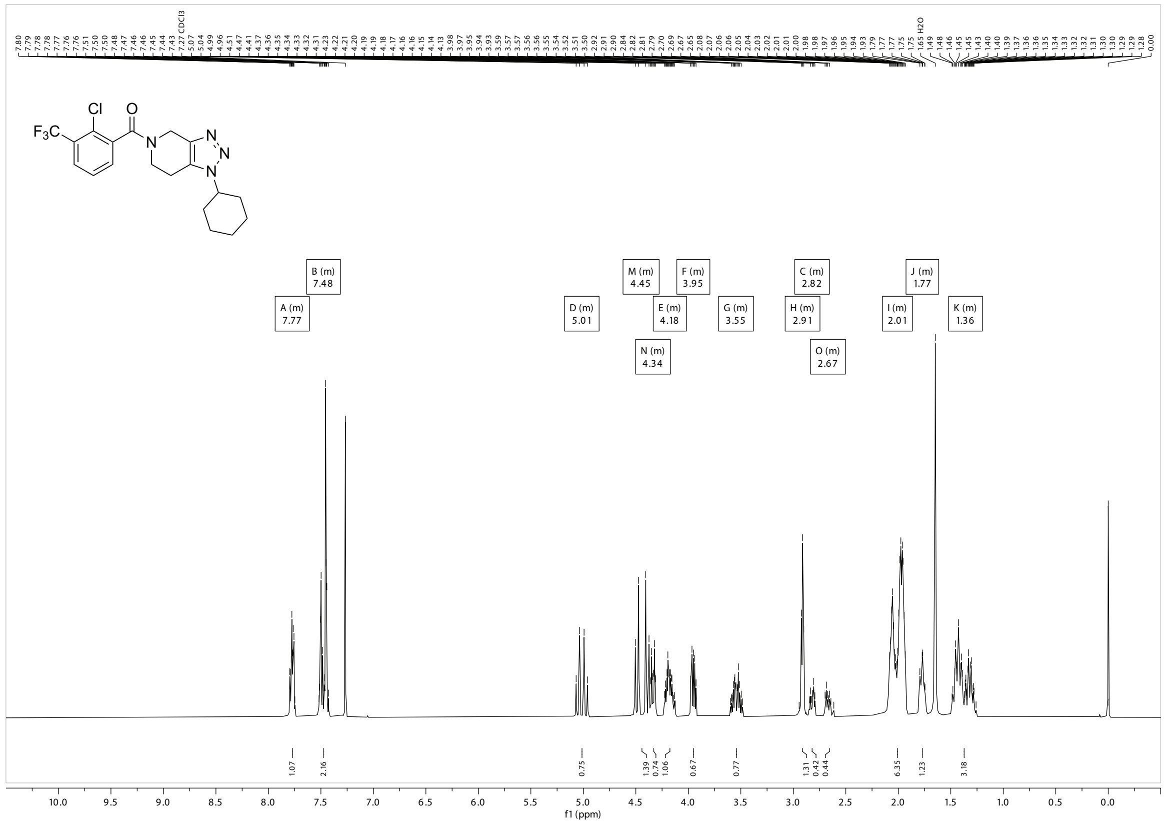

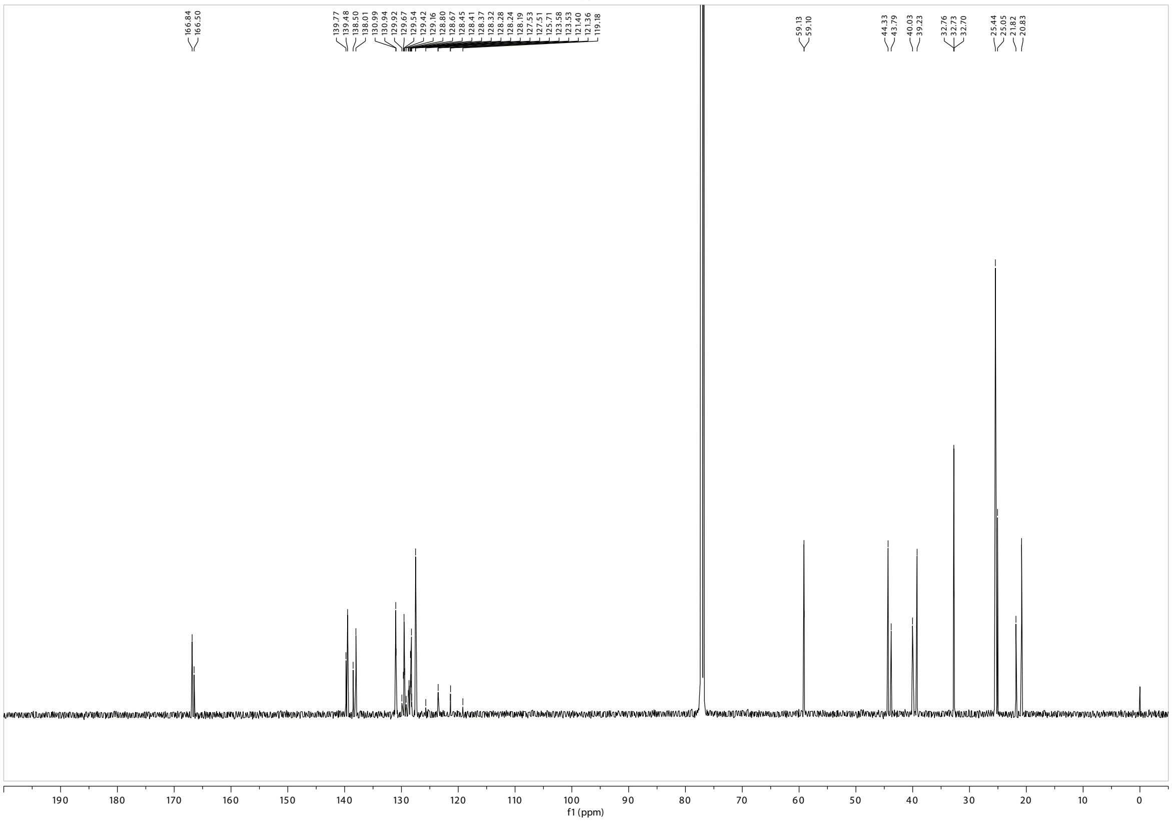

Figure 65: ^{1}H and ^{13}C of compound **2f**

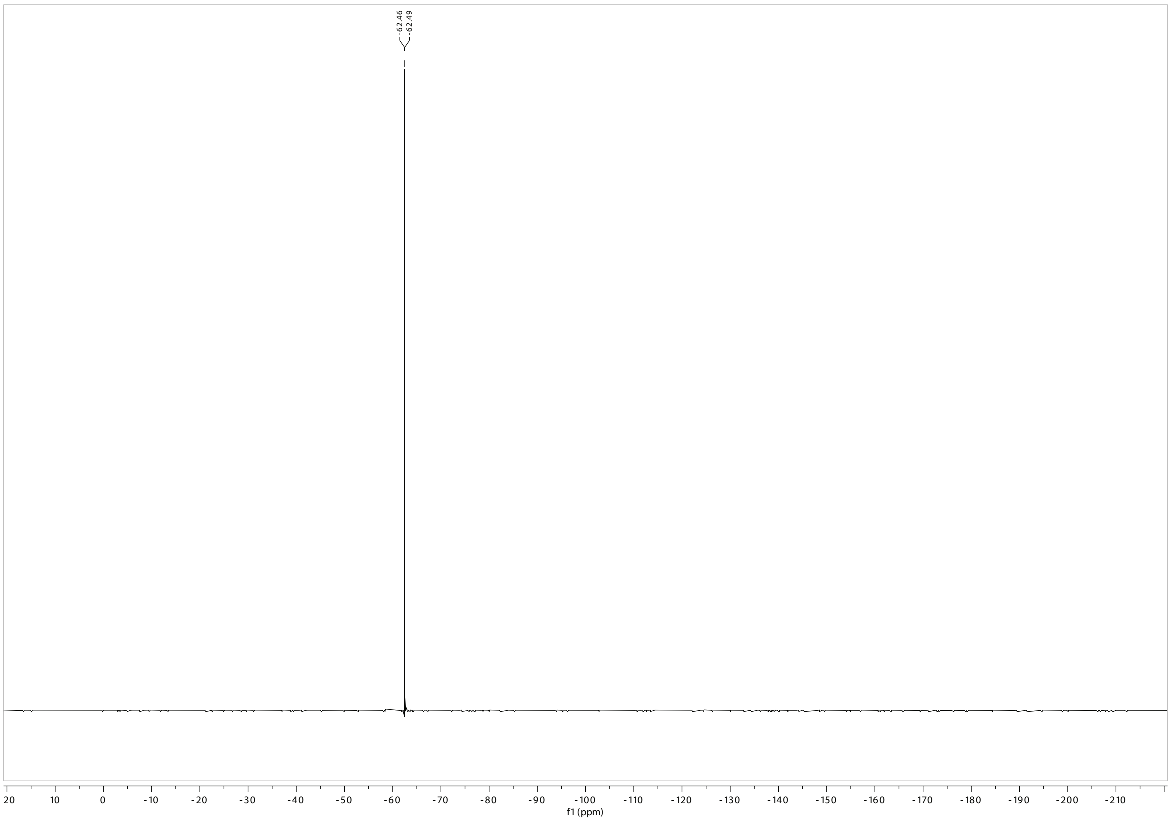

Figure 66: ^{19}F NMR of compound **2f**

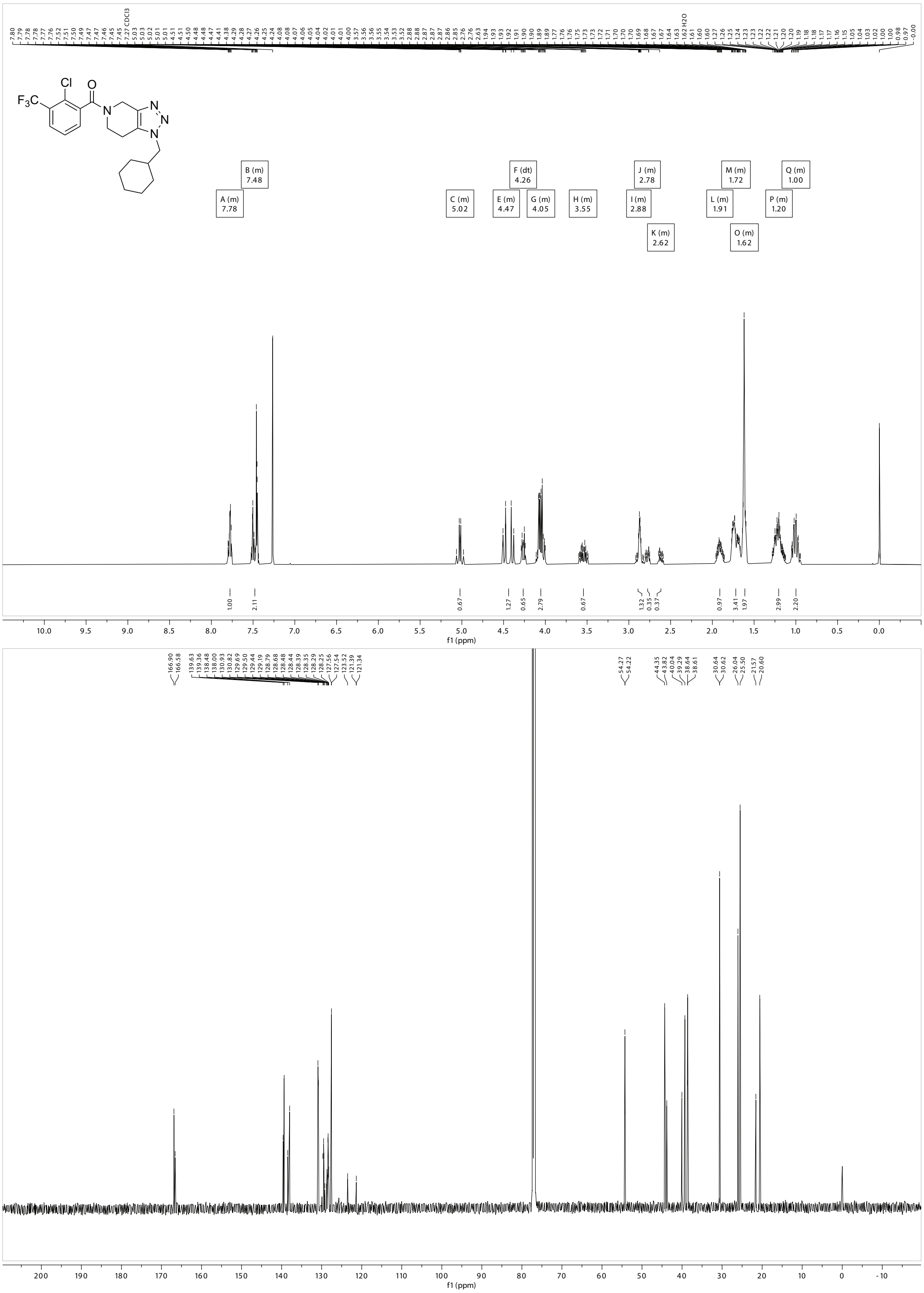

Figure 67: ^{1}H and ^{13}C of compound **2g**

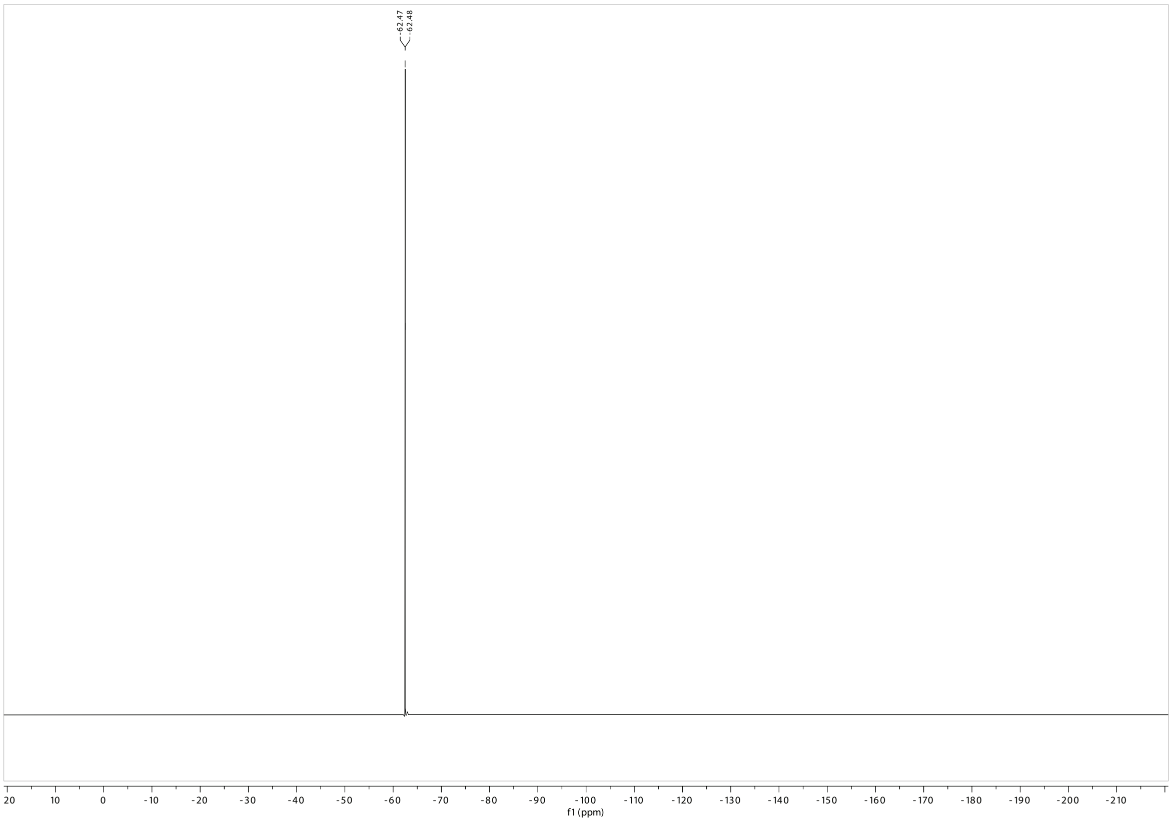

Figure 68: ^{19}F NMR of compound **2g**

Chapter 3

Bibliography

[1] Jackson, A.; Werry, E. L.; O'Brien-Brown, J.; Schiavini, P.; Wilkinson, S.; Wong, E. C. N.; McKenzie, A. D. J.; Maximova, A.; Kassiou, M. Pharmacological characterization of a structural hybrid P2X7R antagonist using ATP and LL-37. *European Journal of Pharmacology* **2022**, *914*, 174667.

[2] Chrovian, C. C. *et al.* A Dipolar Cycloaddition Reaction To Access 6-Methyl-4,5,6,7-tetrahydro-1 H -[1,2,3]triazolo[4,5- c]pyridines Enables the Discovery Synthesis and Preclinical Profiling of a P2X7 Antagonist Clinical Candidate. *Journal of Medicinal Chemistry* **2017**, *61*, 207–223.

[3] Vaca, A.; Ignacio, J.; Gil, A.; Chrovian, C. C.; Jolla, L. P2X7 Modulators. 2014; ISBN: US 2014/0275015 Issue: 73 Volume: 1.

[4] Savall, B. M.; Wu, D.; De Angelis, M.; Carruthers, N. I.; Ao, H.; Wang, Q.; Lord, B.; Bhattacharya, A.; Letavic, M. A. Synthesis, SAR, and Pharmacological Characterization of Brain Penetrant P2X7 Receptor Antagonists. *ACS Medicinal Chemistry Letters* **2015**, *6*, 671–676, Publisher: American Chemical Society.
